# Immunoproteasome Inhibition Positively Impacts the Gut‐Muscle Axis in Duchenne Muscular Dystrophy

**DOI:** 10.1002/jcsm.70054

**Published:** 2025-10-01

**Authors:** Andrea Farini, Francesco Strati, Monica Molinaro, Debora Mostosi, Sabrina Saccone, Luana Tripodi, Jacopo Troisi, Annamaria Landolfi, Chiara Amoroso, Barbara Cassani, Aitor Blanco‐Míguez, Emma Leonetti, Davide Bazzani, Mattia Bolzan, Francesco Fortunato, Flavio Caprioli, Federica Facciotti, Yvan Torrente

**Affiliations:** ^1^ Neurology Unit Fondazione IRCCS Ca' Granda Ospedale Maggiore Policlinico Milan Italy; ^2^ Department of Biotechnology and Biosciences University of Milano‐Bicocca Milan Italy; ^3^ Stem Cell Laboratory, Dino Ferrari Center, Department of Pathophysiology and Transplantation University of Milan Milan Italy; ^4^ Department of Medicine, Surgery and Dentistry, Scuola Medica Salernitana University of Salerno Baronissi Italy; ^5^ Theoreo Srl Spinoff Company of the University of Salerno Montecorvino Pugliano Italy; ^6^ Department of Medical Biotechnologies and Translational Medicine Università Degli Studi di Milano Milan Italy; ^7^ PreBiomics S.r.l. Trento Italy; ^8^ Dino Ferrari Centre, Department of Pathophysiology and Transplantation (DEPT) University of Milan Milan Italy; ^9^ Department of Pathophysiology and Transplantation, Unit of Gastroenterology and Endoscopy Università Degli Studi di Milano, Fondazione IRCCS Ca' Granda, Ospedale Policlinico di Milano Milano Italy

**Keywords:** Duchenne muscular dystrophy, immunoproteasome, macrophages, muscle metabolism

## Abstract

**Background:**

Duchenne Muscular Dystrophy (DMD) features immune‐muscle crosstalk, where muscle fibre degeneration enhances pro‐inflammatory macrophage infiltration, worsening inflammation and impairing regeneration.

**Methods:**

We investigated the impact of immunoproteasome (IP) inhibition on the gut‐muscle axis in mdx mice, a well‐established model of DMD. We employed microbiota perturbation models, including broad‐spectrum antibiotic treatment (ABX) and faecal microbiota transplantation (FMT) from IP‐inhibited mdx mice. IP inhibition effects were assessed by analysing gut microbiota composition, intestinal inflammation, muscle integrity and associated metabolic and inflammatory pathways.

**Results:**

IP inhibitor ONX‐0914 significantly impacted the intestinal inflammatory microenvironment and gut microbiota of mdx mice. ONX‐0914 treatment increased gastrointestinal transit (increased wet/dry faecal weights, *p* = 0.0486 and *p* = 0.0112, respectively) and partially restored intestinal barrier integrity (reduced FITC‐dextran leakage, *p* = 0.0449). JAM‐A was significantly upregulated (*p* < 0.0001). Colonic CD206+ M2 macrophages increased, while CD68 + M1 cells partially decreased. ONX‐0914 downregulated IP isoforms in macrophages (PSMB8: *p* = 0.0022; PSMB9: *p* = 0.0186) as well as FOXO‐1 (*p* = 0.0380) and TNF‐α (*p* = 0.0487). Antibiotic‐induced microbiota depletion abrogated these effects. Metagenomic analysis revealed significant differences in microbiota composition between C57Bl controls and mdx mice (PERMANOVA *p* < 0.001), with ONX‐0914 inducing enrichment of stachyose degradation pathways. Metabolomic analysis showed enrichment of bacterial metabolites, fatty acid and sugar metabolism pathways, with increased glutathione, galactose, glycerol, glyceraldehyde and TCA cycle intermediates. ONX‐0914 improved mitochondrial activity in skeletal muscle, as increased expression of ETC complexes (mdx vs. mdx+ONX: Complex II, *p* = 0.0338; Complex IV, *p* = 0.0023) and TCA enzymes (mdx vs. FTMmdx+ONX: IDH *p* = 0.0258; FH *p* = 0.0366). This led to a shift towards oxidative muscle fibres and improved muscle morphology (increased fibre size, *p* < 0.0001 mdx vs. mdx+ONX and mdx vs. FTMmdx+ONX). Muscle performance was enhanced with reduced CPK levels (*p* = 0.0015 mdx vs. mdx+ONX) and fibrosis (decreased TGFβ: mdx vs. mdx+ONX, *p* = 0.0248; mdx vs. FTMmdx+ONX, *p* = 0.0279). ONX‐0914 reduced CD68+ (mdx vs. mdx+ONX, *p* = 0.0024; mdx vs. FTMmdx+ONX, *p* < 0.0001) and increased CD206+ (mdx vs. FTMmdx+ONX: *p* = 0.0083) macrophages in muscle, downregulated inflammatory genes (mdx vs. mdx+ONX: *ccl2 p* = 0.0327, *vcam‐1p* = 0.0378) and reduced pro‐inflammatory proteins (MCP1, mdx vs. mdx+ONX, *p* = 0.0442). Inflammatory cytokines and endothelial vessel density in ONX‐0914 treated mdx were restored to wild type mice. These data demonstrate that ONX‐0914 enhances muscle function through microbiota‐dependent mechanisms.

**Conclusions:**

Our study advances the understanding of the role of dysbiosis in DMD disease and identifies IP inhibition as a potential therapeutic strategy to modulate the dystrophic gut–muscle axis, offering new perspectives for microbiota‐targeted therapies.

## 1 Introduction

Growing evidence demonstrates crosstalk between the immune system and the skeletal muscle in inflammatory muscle diseases and dystrophic conditions, such as Duchenne Muscular Dystrophy (DMD) as well as during normal muscle regeneration. Most studies in DMD have been conducted using the Dmd^mdx^ (mdx) mouse model [1], which exhibits characteristic features of the human disease, including active myofiber necrosis, cellular infiltration, fat accumulation and fibrosis, leading to significant muscle weakness [2]. Specifically, mdx mice exhibit significant skeletal muscle pathology early in life, characterized by extensive muscle fibre degeneration and a robust regenerative response. These repeated degeneration/regeneration cycles lead to muscle mass replacement, mild endomysial fibrosis and a marked increase in muscle fibres with centralized nuclei [3].

The asynchronous cycles of muscle fibre degeneration in DMD exacerbate muscle infiltration of macrophages and lymphocytes and their secretion of pro‐inflammatory cytokines. Macrophages modulate the activity of satellite cells, the functions of extracellular matrix, and the proliferation of other immune cells [4]: the transition from pro‐inflammatory M1 to anti‐inflammatory M2 types is severely disrupted in DMD, impairing their regulatory role in muscle repair and regeneration and exacerbating disease progression. The severity of muscle injury and inflammation determines the extent of impaired muscle regeneration and the subsequent replacement of myofibers with connective and adipose tissue [5]. Secondary non‐muscle complications can exacerbate the progression of DMD. Specifically, dysfunctions in dystrophin and dystrophin glycoprotein complex (DGC) significantly compromise the structural integrity of the intestinal barrier; as a result, individuals with DMD experience gastrointestinal dysfunctions, such as constipation, pseudo‐obstruction and acute dilatation [6]. Similarly, mdx mice exhibit impaired intestinal contractility, associated with significant abnormalities in mucosal epithelial morphology, typically linked to inflammatory states [7]. The gastrointestinal tract is home to the microbiota, a diverse community of commensal microorganisms that play a crucial role in regulating various essential functions, including immune function, circadian rhythm, nutritional metabolism, behavioural and neurological responses and overall metabolism [8]. Gut microbiota maintain tissue homeostasis by mediating interactions between epithelial cells and the immune system, regulating responses to various antigens and pathogens [9]. The intricately coordinated activation of immune cell subpopulations results in the secretion of specialized metabolites and cytokines, which simultaneously bolster intestinal immunity and cater to the specific functional requirements of each tissue [10]. Given that limiting microorganism‐epithelial interaction is key to gut homeostasis, the inflammatory conditions and membrane alterations in DMD may allow systemic spread of microbial components, which could trigger harmful immune responses and worsen tissue inflammation. Studies have demonstrated that changes in the abundance of intestinal microorganisms can modulate local immune responses [9], inducing proliferation of immune cells and the subsequent inflammatory signals in organs distant from the intestine [11], that could be reversed by antibiotic‐driven microbiota modifications [12].

Consistent with these findings, we recently showcased dysbiosis in mdx mice, elucidating its correlation with alterations in both the peripheral and local immune profiles of mdx mice, alongside muscle integrity. These findings imply that dysfunctions in immune metabolism orchestrated by gut microbiota communities could play an active role in shaping the clinical and phenotypic variability observed in DMD patients [13]. Moreover, Kalkan reported similar alterations of mdx microbiota together with modulations of circulating short‐chain fatty acids (SCFAs), leading to alteration of PPARγ‐dependent pathways in skeletal muscle [14].

To retain protein homeostasis in cells, the ubiquitin–proteasome system removes proteins that are not properly folded or damaged, providing a rapid response to various stimuli and controlling inflammatory development. In particular, the proteasome and its inducible form—the immunoproteasome (IP)—represent the first line of defence in the gut, through the regulation of the NF‐kB pathway and the balancing of the effects on its downstream targets, such as immunity, inflammation and apoptotic cell death [15]. Consistent with this evidence, patients suffering from intestinal problems had modifications in proteasome Supporting Information S1: [S1] and in IP/proteasome balancing Supporting Information S1: [S2]. Interestingly, the absence of IP reduced colonic inflammation and tissue degeneration Supporting Information S1: [S3], while IP subunits PSMB8/PSMB9 are over‐expressed in the colon of animal models of colitis Supporting Information S1: [S4, S5].

In recent years, numerous studies shed light on the association between intestinal microbiota and various aspects of skeletal muscle, including mass, function and metabolism [16, 17]. These coordinated events play a crucial role in maintaining metabolic balance, insulin sensitivity and overall inflammation throughout the body. Pre‐clinical studies involving germ‐free mice [17, 18] as well as mdx mice [13] demonstrated that the absence or imbalance of gut microbiota can induce metabolic and morphological alterations in skeletal muscles. Here, we demonstrate that mdx mice treated with the IP inhibitor ONX‐0914—a well‐known modulator of inflammation, cytokine production and mitochondrial respiration in dystrophic mice [19, 20, 21]—exhibit reduced inflammation in the intestinal barrier and enhanced intestinal motility, leading to a reshaping of gut microbiota composition. Furthermore, ONX‐0914 induced a distinct metabolic signature, including increased levels of glycerol, glyceraldehyde and TCA cycle intermediates, reflecting modulation of systemic energy metabolism.

Microbiota perturbation experiments, including FMT, confirmed that the effects of ONX‐0914 on muscle metabolism and function depend on a functional microbiota. ONX‐0914 treatment increased the proportion of oxidative/glycolytic MyHC‐IIa/IIx fibres and improved muscle fibre size and performance, with a reduction in muscle damage and fibrosis. Interestingly, microbiota‐depleted mice failed to respond to these changes, indicating that the beneficial effects of ONX‐0914 are microbiota‐dependent. Additionally, ONX‐0914 treatment modulated macrophage polarization in skeletal muscle and restored CD31+ and isolectin+ vessels to wild type levels, further supporting the therapeutic potential of ONX‐0914 in modulating inflammation and muscle function through microbiota‐dependent mechanisms.

## 2 Methods

The main reagents and animal models necessary for replicating the reported results as well as the detailed methodological tools are listed in Supporting Information.

### 2.1 Animal Experiments

Procedures involving living animals were conducted in compliance with Italian law (D.L.vo 116/92) and approved by local ethics committees, following authorization from the Ministry of Health and the Local University of Milan Committee (protocol numbers: 10/13–2014/2015 and 859/2017‐PR). 3‐month‐old C57Bl/10 mice (referred to as 3 m C57Bl) and C57BL/10ScSn‐Dmdmdx/J mice (referred to as 3 m mdx) were obtained from Charles River.

### 2.2 Shotgun Microbiota Analysis

Stool samples from 3 m C57Bl, mdx and mdx+ONX (*n* = 8 each) were processed, and the DNA was isolated by using the DNeasy 96 PowerSoil Pro QIAcube HT Kit (Qiagen, #47021). The DNA was quantified by using the Quant‐iT 1X dsDNA Assay Kits, BR (Life Technologies, #Q33267) in combination with the Varioskan LUX Microplate Reader (Thermo Fisher Scientific, #VL0000D0). Diluted DNA was used for library preparation and sequencing; pre‐processing and quality control; taxonomic and functional profiling [22] as detailed in Supplementary Methods.

### 2.3 Histological and Immunofluorescence Analysis of Tissue Sections

To quantify slow/Type I, fast fatigue resistant/Type IIa and fast fatigable/Type IIb muscle fibres, we performed staining for either myosin ATPases or oxidative enzyme capacity (succinate dehydrogenase, SDH). Picro Sirius Red staining was performed to assess the percentage of fibrosis. For immunofluorescence staining, muscle and colon sections were fixed with 4% paraformaldehyde for 10 min, permeabilized with 0.1% Triton X‐100 for 10 min and incubated with 10% donkey serum to block non‐specific binding for 1 h. For image processing and quantification of the fluorescence, we used both the ImageJ Software Supporting Information S1: [S6] and NIS‐Elements v.5.30 (Nikon Instruments); we analysed the data obtained through GraphPad PrismTM. Ad‐hoc established workflows of image processing, binarisation and segmentation were employed for semi‐automatic unbiased analysis of immunofluorescence performed on muscular tissues. All these techniques are described in detail in Supplementary Methods.

## 3 Statistics

To allocate the animals to different experimental procedures, we used the randomization within blocks. To avoid that the efficacy of ONX‐0914 treatment on mdx mice was not correctly interpreted worsening the reliability of our results, animal handlers were blinded regarding the treatment that the mice received throughout all the experimental procedures. Animals that suffered from clinical complications as enhancement of stress or motor impairments were excluded from the experimental plan and eventually sacrificed. Sample‐size calculator freely available on internet was used to assess sample size. To determine significance when comparing multiple groups' means, we used One‐way ANOVA followed by Tukey's multiple comparison test, while Student's *t*‐test was used to compare two groups assuming equal variances. In the case of a non‐parametric test, we performed the Kruskal–Wallis test. For repeated measures, statistical significance was calculated via simple linear regression by testing for differences between the slopes of best‐fit lines (a 95% confidence band of each best‐fit line is shown). Diversity analyses were carried out on samples subsampled to 35 M microbial reads. Multidimensional Scaling (MDS) on arcsine square root‐transformed abundances was performed using Jaccard and Bray–Curtis distance metrics. PERMANOVA and Mann–Whitney tests were performed using the SciKit‐bio python library. To identify associations between the metadata and the abundance of microbial species and pathways, LefSe (version 1.0) Supporting Information S1: [S7] was applied on the relative abundance data. Alpha and beta diversity analyses were performed on the taxonomic and functional profiles using the scikit‐bio (version 0.5.6), scikit‐learn (version 1.2.2) and SciPy (version 1.10.1) Python libraries.

## 4 Results

### 4.1 ONX‐0914 Treatment of Mdx Mice Affects Intestinal Inflammatory Microenvironment and Results in Microbiota Modulation

**FIGURE 1:**
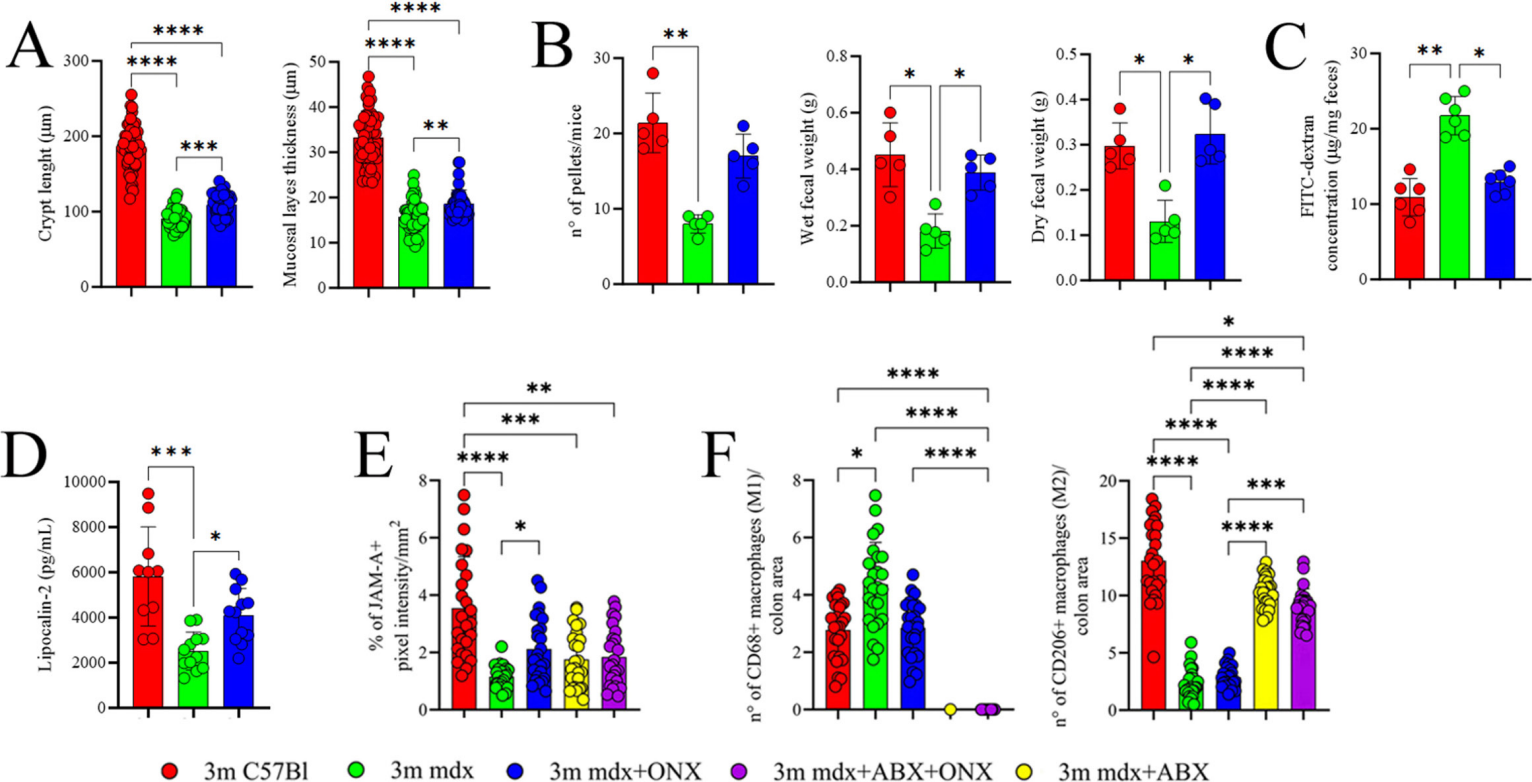
Colonic features of 3 m mdx+ONX‐0914 mice. (**A**) Mucus layer thickness and crypt length were quantified for *n* = 5 mice per group (with pooled samples of *n* = 10 each). (**B**) The number of pellets and the weight of faeces from 3 m C57Bl, mdx and mdx+ONX mice (*n* = 5 each). (**C**) Evaluation of FITC‐dextran concentration in the faecal stools of 3 m C57Bl, mdx and mdx+ONX mice (*n* = 6 each) (two independent experiments). (**D**) Evaluation of Lipocalin‐2 concentration in the stool of 3 m C57Bl (*n* = 5), mdx and mdx+ONX mice (*n* = 6 each) (two independent experiments). (**E**) The data are showed in graph as the percentage of JAM‐A+ pixel intensity per mm^2^. (**F**) The data are showed in graphs as the number of CD68+ and CD206 + macrophages by the area of colonic sections. Data information: data are presented as mean ± SD (*p < 0.05; **p < 0.01, ***p < 0.001, ****p < 0.0001; One‐Way ANOVA Kruskal–Wallis test for evaluation of images and One‐Way ANOVA with Tukey's multiple comparisons test for WB experiments).

Following up studies on our previous work resolving IP biology in mdx mice [20], this mouse strain was treated with ONX‐0914 and characterized for modifications of the intestinal inflammatory microenvironment. Thereby, histological features of 3 m colon mdx—as the submucosal layer's thickness and crypt length—were mildly affected by ONX‐0914 (Figures 1A and S1A). Moreover, ONX‐0914‐treated mdx mice exhibited increased gastrointestinal transit compared to untreated mdx mice: both wet and dry faecal weights were higher in ONX‐0914‐treated mice (*p* = 0.0486 and *p* = 0.0112, respectively), indicating an enhancement in motor activity within the large intestine and an improvement in fluid reabsorption in the treated group (Figure 1B). To further investigate the integrity of the gut barrier, we injected the mice with fluorescein‐isothiocyanate‐labelled dextran (FITC‐dextran) and evaluated the concentration of FITC‐dextran in the faecal stools as a direct measure of intestinal permeability. FITC‐dextran was up‐regulated in mdx mice and partially rescued in mdx+ONX‐0914 mice (*p* = 0.0449) (Figure 1C). Faecal lipocalin 2 (Lcn2) and *IL‐10* level—multifunctional innate immune proteins linked to intestinal inflammation and gut dysbiosis—were significantly reduced in mdx compared to age‐matched C57Bl (mdx vs. C57Bl *p* = 0.0001) (Figure 1D). Interestingly, treatment with ONX‐0914 led to a significant increase in Lcn2 (mdx vs. mdx+ONX *p* = 0.0217) and IL‐10 levels in mdx mice (Figures 1D and S1C). Moreover, Junctional Adhesion Molecule‐A (JAM‐A), a protein crucial for intestinal homeostasis and implicated in IBD Supporting Information S1: [S8, S9], was down‐regulated in mdx mice compared to age‐matched C57Bl and significantly increased following ONX‐0914 treatment (Figures 1E and S1B).

In the colonic tissues of mdx mice, the extent of anti‐inflammatory CD206+ M2 macrophages by the area of colon sections, did not change following ONX‐0914 treatment, while the amount of pro‐inflammatory CD68+ M1 cells was elevated in mdx mice and partially rescued in treated animals (Figures 1F and S1B). Macrophages isolated from ONX‐0914‐treated mdx mice showed down‐regulation of the expression of IP isoforms PSMB8 and PSMB9 (Figure S1C). TLR‐dependent pathways are critical mediators that regulate proliferation and polarization of macrophages during DMD Supporting Information S1: [S10, S11]. However, the expression of AKT and TLR2 was similar to untreated mdx (Figure S1C). Next, we investigated the expression of FOXO‐1, which serves as an activator of macrophage inflammasome, leading to IL‐1β transcriptional upregulation and ROS over‐production Supporting Information S1: [S12]. In line, we found an over‐expression of this protein together with other pro‐inflammatory mediators, such as TNF‐α, IL‐6 and TGF‐β (Figure S1C).

Depletion of the gut microbiota with antibiotics (ABX) abrogated the effects of ONX‐0914 on its intestinal targets in mdx mice. Specifically, ONX‐0914 failed to significantly reduce PSMB8 and PSMB9 expression or enhance JAM‐A levels and M2 macrophage polarization in mdx mice treated with ABX (mdx+ABX+ONX), mirroring the levels observed in mdx mice receiving only ABX (mdx+ABX) (Figure S1D). These findings demonstrate that ONX‐0914's efficacy on intestinal inflammation is dependent on the presence of a functional gut microbiota.

**FIGURE 2:**
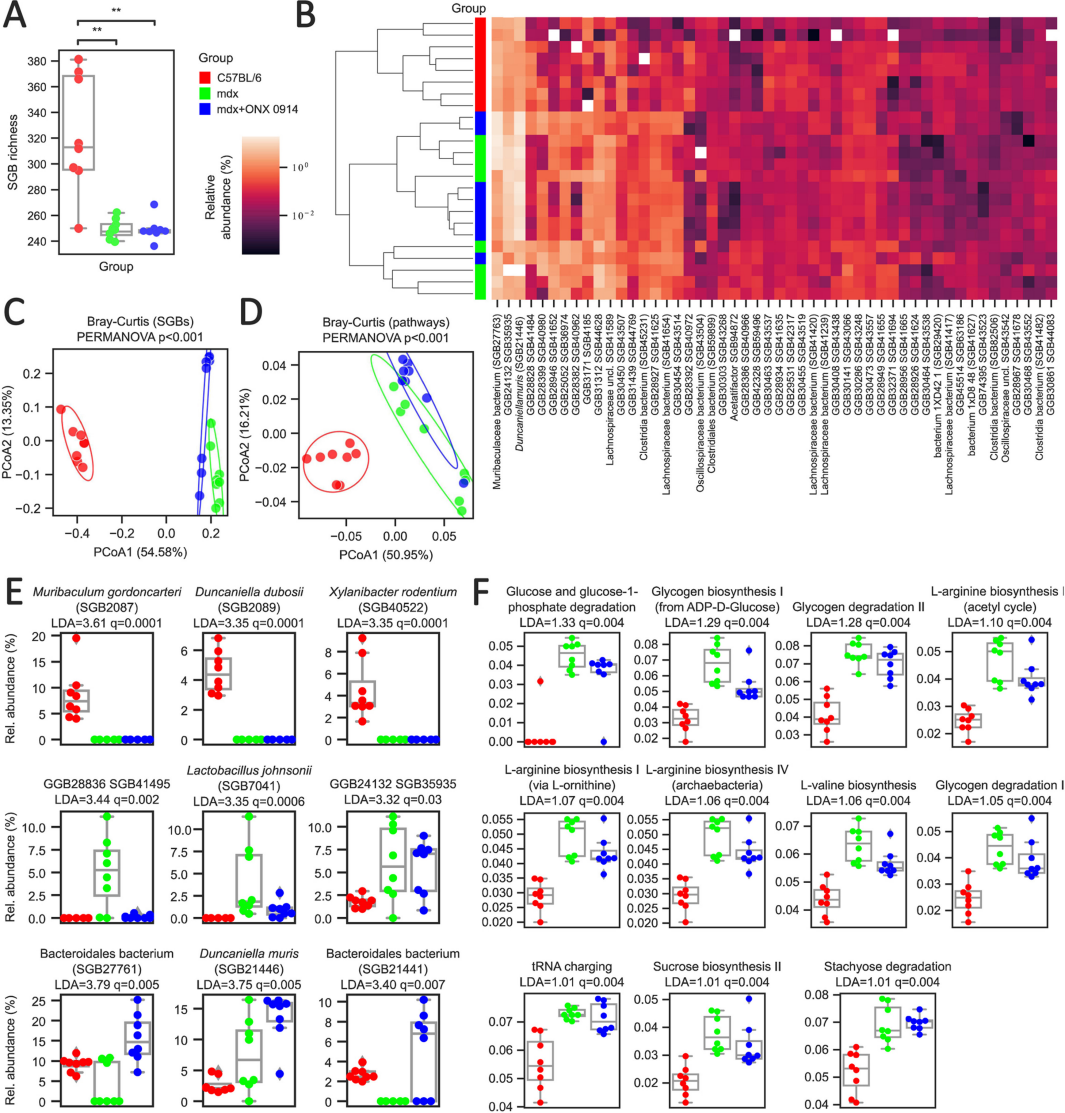
Dysbiotic microbiota of 3 m mdx mice is modulated by ONX‐0914. **(A)** Species‐level genome bin (SGB) richness in 3 m C57Bl (*n* = 8), mdx (*n* = 8) and mdx + ONX (*n* = 8). Mann–Whithey tests were performed (** = *p* < 0.01) (**B**) Heatmap of the top 50 SGBs based on their prevalence in 3 m C57Bl (*n* = 8), mdx (*n* = 8) and mdx + ONX (*n* = 8). Samples were clustered based on Bray–Curtis dissimilarity on the SGB relative abundans. **(C)** MDS of beta‐diversity of 3 m C57Bl (*n* = 8), mdx (*n* = 8) and mdx+ONX (*n* = 8) as measured by Bray–Curtis dissimilarity on the SGB‐level relative abundances. PERMANOVA tests (1000 iterations) were performed. **(D)** MDS of beta‐diversity of 3 m C57Bl (*n* = 8), mdx (*n* = 8) and mdx+ONX (*n* = 8) as measured by Bray–Curtis dissimilarity on the microbial pathway relative abundances. (**E**) Relative abundance of the SGBs significantly associated with 3 m C57Bl (*n* = 8)—upper panel –; mdx (*n* = 8)—middle panel –; mdx+ONX (*n* = 8)—lower panel—in the LefSe analysis (FDR < 0.05). Linear Discriminant Analysis (LDA) score and FRD (*q* = Benjamini/Hochberg correction) are reported. **(F)** Relative abundance of the microbial pathways significantly associated with mdx (*n* = 8) and mdx + ONX (*n* = 8)—stachyose degradation—in the LefSe analysis (FDR < 0.05). LDA score and FRD (*q* = Benjamini/Hochberg correction) are reported.

**FIGURE 3:**
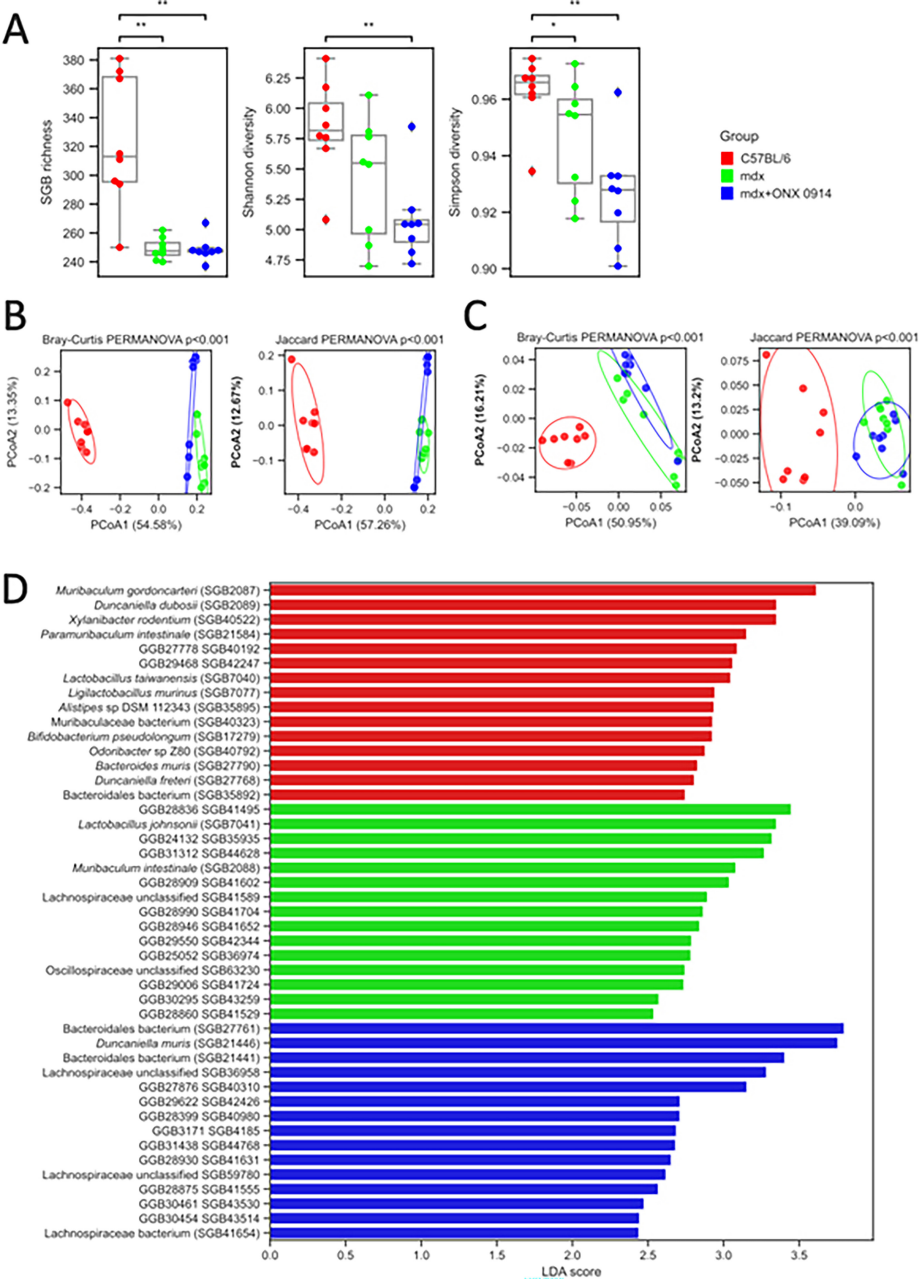
Microbial community analysis in 3‐month‐old C57Bl, mdx and mdx + ONX mice. **(A)** Alpha diversity differences between groups. SGB richness, Shannon and Simpson diversity were computed. Significance was assessed using Mann–Whitney tests. Only significant comparisons are shown. (*: 0.01 < *p* < = 0.05; **: 0.001 < *p* < = 0.01). **(B)** MDS based on beta diversity for taxonomic composition. Bray–Curtis and Jaccard distances were computed on the arcsine square root‐transformed relative abundances. Significant differences were computed using PERMANOVA tests (1000 iterations). **(C)** MDS based on beta diversity for microbial pathways. Bray–Curtis and Jaccard distances were computed on the arcsine square root‐transformed relative abundances. Significant differences were computed using PERMANOVA tests (1000 iterations). **(D)** Linear Discriminant Analysis (LDA) score of the top 15 SGBs significantly associated with 3 m C57Bl (*n* = 8), mdx (*n* = 8) and mdx+ONX (*n* = 8) in the LefSe analysis (FDR < 0.05).

Since the commensal microbiota retains a key role in the pathogenesis of intestinal inflammation [23, 24, 25] and dysbiosis has been demonstrated in mdx mice [13, 14], we next investigated gut bacterial composition using shotgun metagenomic analysis. Alpha diversity analysis—based on species‐level genome bin (SGB), [26] richness and Shannon and Simpson diversity—shows significant differences between the C57Bl group and both mdx and mdx+ONX groups, in accordance with our previous data with mdx mice [13] (with the exception of the C57Bl vs. mdx comparison for Shannon diversity; *p* < 0.05; Figures 2A and 3A). Evaluation of Bray–Curtis dissimilarity based on the SGB relative abundances assessed how the structure of microbial community among groups was significantly different (PERMANOVA *p* < 0.001; Figures 2B,C and 3B). Similar results were obtained based on Bray‐Curtis dissimilarities on the microbial pathways relative abundances (PERMANOVA *p* < 0.001; Figures 2D and 3C). We carried out a three‐group biomarker‐discovery analysis to understand the specific species that were differentially abundant between groups using LefSe Supporting Information S1: [S7] (Figures 2E and 3D). The main biomarkers of the C57Bl were mostly known microbial species, such as *Muribaculum gordocarteri*, *Duncaniella dubosi or Xylanibacter rodentium* and were found totally absent in the other groups. Instead, biomarkers from the mdx and mdx+ONX groups were (with few exceptions as *Lactobacillus johnsonii* or *Duncaniella muris* ) mostly enriched in still‐non‐cultivated members of the mice gut microbiome and still prevalent in the other groups (Figures 2E and 3D). We repeated a biomarker‐discovery analysis to understand the specific microbial pathways that were differentially abundant between groups (Figure 2F). Results at the pathway level were significantly less in comparison to those using the taxonomic profiles. We did not find any microbial pathway enriched in the C57Bl group. Instead, we found 10 pathways enriched in the mdx group, mostly related to glucose, glycogen, L‐arginine and sucrose biosynthesis and/or degradation and only 1 (stachyose degradation) enriched in the mdx+ONX group (Figure 2F). The few results found suggest that the functional potential analysis is still pretty biased by the high amounts of uncharacterized microbes and functions of the mice microbiome.

To address the potential influence of stress induced by the intraperitoneal injection procedure on the microbiota Supporting Information S1: [S13, S14], we conducted a comparison of microbiota composition between 3 m mdx mice (CTRL) and age‐matched mdx mice treated solely with the vehicle solution (VEH) (*n* = 4 per group) (Figure S2). The analysis of alpha‐ and beta‐diversity showed no significant differences between CTRL and VEH mdx mice, suggesting that the intestinal microbial community structure of mdx mice is similar to that of vehicle‐treated ones.

### 4.2 ONX‐0914 Influences Gut‐Muscle Axis Metabolism, Ameliorating Dystrophic Muscle Features

**FIGURE 4:**
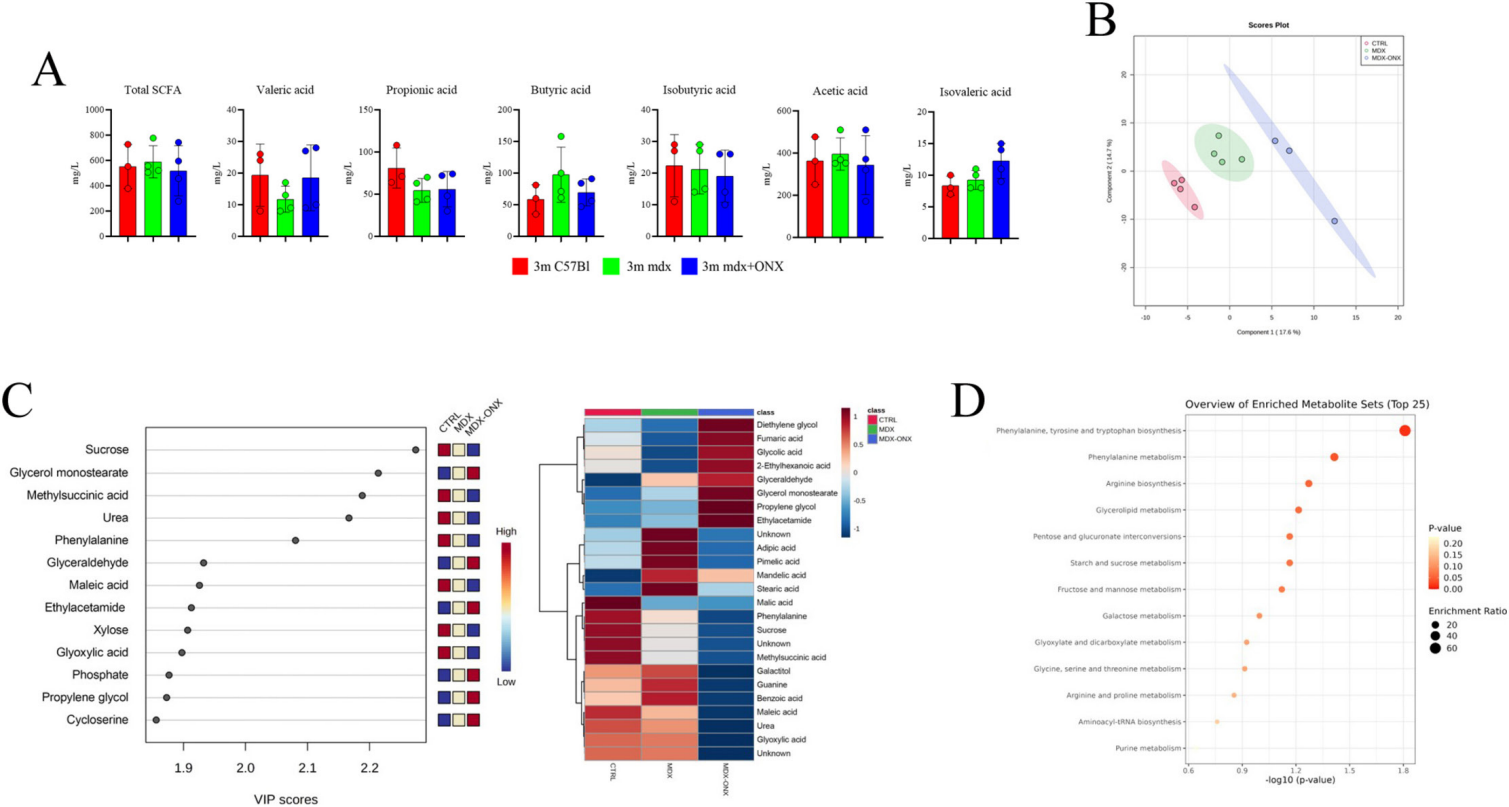
Evaluation of metabolome of 3 m mdx+ONX‐0914 serum. (**A**) SCFA faecal quantification of 3 m C57Bl (*n* = 3), mdx (*n* = 4) and mdx + ONX (*n* = 4) mice. (**B**) Partial Least Square Discriminant Analysis score plot of CTRL (red, *n* = 4), mdx (green, *n* = 4) and mdx + ONX (blue, *n* = 3) serum samples. In brackets are reported the amount of explained variance for each latent component. (**C**) Concentration of the metabolites with a variable importance in projection (VIP) score higher than 1.5 isolated from serum samples. Heatmap of the concentration of the top 25 metabolites according to ANOVA. (**D**) Metabolic pathway showing the interplay of the VIP selected metabolites.

To further investigate the impact of ONX‐0914 on host metabolism, we performed a targeted metabolomic analysis of the small intestine of 3 m C57Bl, mdx and mdx+ONX mice. Pathway reconstruction, focusing on the most discriminatory metabolites, demonstrated a significant enrichment of pathways related to bacterial metabolite production, fatty acid synthesis and degradation and simple sugar metabolism (Figure S3A). Subsequent analysis of key differential metabolites using MetPa emphasized the importance of glutathione, inositol phosphate and galactose metabolism (Figure S3B). The significance of these metabolites, glutathione and galactose, is underscored by their diverse roles in maintaining cellular homeostasis. Glutathione is crucial for mitigating oxidative stress, regulating cell proliferation and apoptosis and modulating cytokine production and immune responses Supporting Information S1: [S15]; and galactose's contribution to metabolic regulation, protein function and immune modulation via its transport throughout the bloodstream Supporting Information S1: [S16]. However, the ONX‐0914 treatment did not significantly affect overall short‐chain fatty acid (SCFA) content in mdx mice (Figure 4A). This suggests a potential fine‐tuning of SCFA production and utilization. Furthermore, ONX‐0914 treatment induced a distinct circulating metabolic signature in mdx mice compared to both C57Bl and untreated mdx controls (Figure 4B). Specifically, we observed a significant increase in serum levels of glycerol and glyceraldehyde (Figure 4C), two key intermediary metabolites derived from triglyceride and fructose breakdown, respectively. Elevated glycerol levels under fasting conditions are often associated with increased lipolysis and subsequent gluconeogenesis Supporting Information S1: [S17], while glyceraldehyde can be converted to glycerol during caloric restriction or enter the glycolytic pathway under physiological conditions Supporting Information S1: [S18]. Consistent with these observations, ONX‐0914‐treated mice also exhibited increased serum levels of tricarboxylic acid (TCA) cycle intermediates, such as glycolic and fumaric acids (Figure 4C), further supporting modulation of energy metabolism. Finally, metabolite set enrichment analysis, coupled with corresponding GO pathway analysis, revealed a significant upregulation of pathways related to the biosynthesis of arginine, phenylalanine, tyrosine and tryptophan, as well as glycerolipid metabolism (Figure 4D).

To define whether ONX‐0914‐modified serum metabolites modulate mitochondrial enzymes in skeletal muscles, we assessed the activity of the TCA cycle and Electron Transport Chain (ETC) in tibialis anterior (TA) and diaphragm (DIA) of untreated and ONX‐0914‐treated mdx mice. The ONX‐0914 treatment significantly rescued the expression in TA of NADH cytochrome C reductase (Complex III, C3) and cytochrome oxidase (Complex IV, C4), thus improving the expression of succinate cytochrome C reductase (Complex II, C2) (Figure S4), suggesting that ONX‐0194 improved the activity of skeletal muscle mitochondria.

To further validate the effect of ONX‐0914‐modified serum metabolites on muscle function, we generated two dystrophic mouse models with microbiota perturbation. Specifically, we performed faecal microbiota transplantation (FMT), transferring the microbiota from mdx and mdx+ONX mice into mdx recipients (FTM^mdx^ and FTM^mdx+ONX^ mice, respectively). Additionally, we administered ONX‐0914 to 3 m mdx mice with a depleted microbiota (mdx+ABX). While no modifications in TOMM20 and DRP1 were observed in ONX‐0914‐treated and microbiota‐perturbed mdx mice (Figure S5A,B), we found a significant upregulation of C2 and C4 OXPHOS proteins in ONX‐0914‐treated mdx mice. A similar trend, although not statistically significant, was observed in TA of FTM^mdx+ONX^ treated mice but not in mdx+ABX mice (Figure S5A,B). This suggests that ONX‐0914 improves mitochondrial activity without affecting mitochondrial dynamics and that this effect may be dependent on the gut microbiota.

Considering the mounting evidence regarding the crosstalk between mitochondria and proteasome Supporting Information S1: [S19, S20], we evaluated the levels of TCA enzymes. The Oxoglutarate Dehydrogenase (OGDH) and Isocitrate Dehydrogenase (NADP(+)) 2 (IDH2) were significantly upregulated in C57Bl muscles compared to mdx ones (*p* = 0.0062 and *p* = 0.0427, respectively). Notably, IDH2 and Fumarate Hydratase (FH) were upregulated in mdx mice treated with FTM^mdx+ONX^ (mdx vs. FTM^mdx+ONX^, *p* = 0.0258 and *p* = 0.0366, respectively) but not in mdx+ABX+ONX mice (Figure S5A,B). These findings further support the role of ONX‐0914 in modulating mitochondrial activity of dystrophic muscle through microbiota‐dependent mechanisms.

**FIGURE 5:**
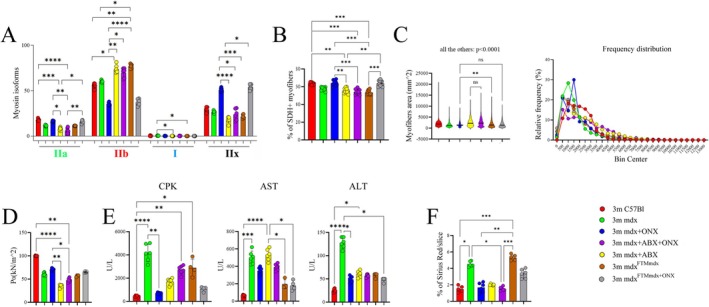
The muscle response to ONX‐0914 is dependent on the gut microbiota. (**A**) Graph portrays the percentage of myofibers expressing different MyHC isoforms in TAs of C57Bl, mdx, mdx+ABX, mdx+ONX and mdx+ABX+ONX mice and mdx^FTMmdx^ and mdx^FTMmdx+ONX^ mice (*n* = 3 each and *n* = 10 images per animal) (two independent experiments). (**B**) Quantification of the percentage of SDH+ myofibers of TAs from C57Bl, mdx, mdx+ABX, mdx+ONX and mdx+ABX+ONX mice (*n* = 5 each and *n* = 8 images per animal) and mdx^Fmdx^ and mdx^Fmdx+ONX^ mice (*n* = 4 each and *n* = 10 images per animal) (two independent experiments). (**C**) Quantification of myofiber area and relative frequency of the myofiber CSA expressed as the frequency distribution of the TA muscles of C57Bl, mdx, mdx+ABX, mdx+ONX and mdx+ABX+ONX mice and mdx^FTMmdx^ and mdx^FTMmdx+ONX^ mice (*n* = 4 each; pooled samples per group: *n* = 5971 for C57Bl, *n* = 6301 for mdx, *n* = 7386 for mdx+ONX, *n* = 2535 for mdx+ABX; *n* = 9737 for mdx+ABX+ONX; *n* = 11 575 for mdx^Fmdx^; *n* = 10 687 for mdx^Fmdx+ONX^). (**D**) Evaluation of tetanic force in TA of C57Bl, mdx, mdx+ABX, mdx+ONX and mdx+ABX+ONX mice (*n* = 6 each) and mdx^Fmdx^ and mdx^Fmdx+ONX^ mice (*n* = 4 each). (**E**) Sera CPK analysis of C57Bl, mdx, mdx+ABX, mdx+ONX and mdx+ABX+ONX mice (*n* = 6 each) and mdx^Fmdx^ and mdx^Fmdx+ONX^ mice (*n* = 4 each). (**F**) Quantification of the percentage of fibrosis of TAs from C57Bl, mdx, mdx+ABX, mdx+ONX and mdx+ABX+ONX mice (*n* = 3 each and *n* = 10 images per animal) and mdx^Fmdx^ and mdx^Fmdx + ONX^ mice (*n* = 3 each and *n* = 10 images per animal) (two independent experiments). Data information: data are presented as mean ± SD (*p < 0.05; **p < 0.01, ***p < 0.001, ****p < 0.0001; One‐Way ANOVA Kruskal–Wallis test).

**FIGURE 6:**
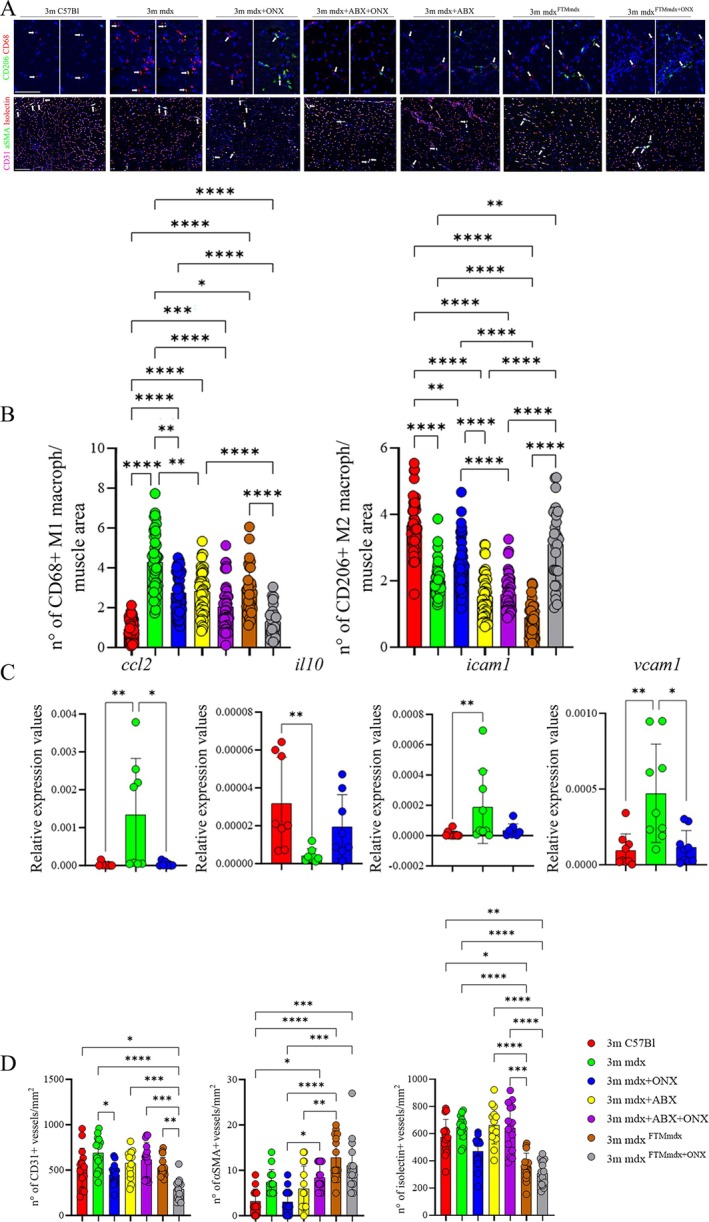
Muscle‐resident pro‐inflammatory macrophages are diminished in ONX‐0914 mdx treated mice. (**A**) CD68+ M1 and CD206+ M2 macrophages were quantified in TA of 3 m C57Bl, mdx, mdx+ABX, mdx+ONX and mdx+ABX+ONX mice (*n* = 6 each and *n* = 8 images per animal) and in mdx^FTMmdx^ and mdx^FTMmdx+ONX^ mice (*n* = 4 each and *n* = 8 images per animal). CD206 staining is shown in green, CD68 in red and DAPI in blue. CD68+ cells are indicated by broken arrow in left panel, while cells co‐expressing CD68 and CD206 are indicated in the right panel. Scale bars: 50 μm. CD31+, αSMA+ and isolectin+ vessels were quantified in TA of 3 m C57Bl, mdx, mdx+ABX, mdx+ONX and mdx+ABX+ONX mice (*n* = 3 each and *n* = 5 images per animal) and in mdx^FTMmdx^ and mdx^FTMmdx+ONX^ mice (*n* = 4 each and *n* = 4 images per animal). CD31 staining is shown in purple, αSMA in green, isolectin in red and DAPI in blue. αSMA+ vessels are indicated by broken arrow. Scale bars: 100 μm. (**B**) The data are showed in graphs as the number of CD68+ and CD206+ macrophages by the area of muscular sections. (**C**) RT‐PCR analysis of two independent experiments showed the modulation of genes normally expressed in M1‐ (*ccl2*, *icam‐1, vcam‐1*) and M2‐ (*IL‐10*) macrophages in muscles of 3 m C57Bl, mdx and mdx+ONX (*n* = 4 each) mice. (**D**) The data are showed in graphs as the number of CD31+, αSMA+ and isolectin+ vessels by mm^2^ of muscular sections. Data information: data are presented as mean ± SD (*p < 0.05; **p < 0.01, ***p < 0.001, ****p < 0.0001; One‐Way ANOVA Kruskal–Wallis test).

To assess whether the ONX‐0914‐induced modulation of mitochondrial activity influences fibre‐type switching, the immunoreactivity for adult myosin heavy chain (MyHC) isoforms was analysed and quantified by immunofluorescence. Compared to untreated mdx mice, ONX‐0914‐ and FTM^mdx+ONX^‐treated mdx mice exhibited a significant increase in the percentage of oxidative/glycolytic MyHC‐IIa/IIx fibres, while the proportion of glycolytic MyHC‐IIb fibres was markedly reduced (Figures 5A and S6). Interestingly, ONX‐0914 treatment also led to a slight but noticeable increase in the percentage of slow oxidative Type I fibres, which are typically characterized by a high mitochondrial content and rely predominantly on aerobic metabolism for ATP production (Figures 5A and S6). Furthermore, ONX‐0914 and FTM^mdx+ONX^ treatments restored the percentage of succinate dehydrogenase‐positive (SDH+) fibres in the TA of mdx mice to levels comparable to those observed in wild‐type C57Bl/6 mice (Figures 5B and S6). In contrast, both mdx+ABX+ONX and mdx+ABX mice displayed a similarly high proportion of MyHC‐IIb fibres and SDH+ myofibres, comparable to mdx mice, indicating that microbiota depletion interferes with the metabolic shift induced by ONX‐0914 (Figures 5A,B and S6). This metabolic remodelling was further supported by an increase in mTOR and its effector 4EBP1/4EBP1p in ONX‐0914 and FTM^mdx+ONX^ treated mdx mice, suggesting reduced protein catabolism and improved anabolic balance (Figure S5A,B). In terms of muscle morphology, morphometric analysis of TA cross‐sectional area (CSA) revealed a significant increase in fibre size in ONX‐0914‐ and FTM^mdx+ONX^‐treated mdx mice but not in mdx+ABX+ONX animals, compared to mdx mice, where smaller CSAs are typically associated with ongoing muscle regeneration (Figures 5C and S6). Frequency distribution analysis further confirmed a shift towards larger myofibre areas in ONX‐0914‐treated mdx and FTM^mdx+ONX^ mice relative to both untreated and mdx+ABX+ONX age‐matched dystrophic mice (Figure 5C). Of note, muscle performance was significantly enhanced in ONX‐0914‐ and FTM^mdx+ONX^‐treated mdx mice compared to untreated and ABX+ONX‐treated mdx mice, as evidenced by a substantial increase in tetanic muscle force (Figure 5D). This improvement was accompanied by a significant reduction in serum creatine kinase levels (mdx vs. mdx+ONX, *p* = 0.0015), indicating decreased muscle damage in ONX‐0914‐ and FTM^mdx+ONX^‐treated mdx mice compared to untreated mdx mice (Figure 5E). In line, we found that ONX treatment positively impacted on fibrosis development, which was diminished related to untreated mdx mice (Figures 5F and S6). Moreover, the fibrotic marker TGFβ was significantly decreased in FTM^mdx+ONX^‐treated mdx mice compared to untreated mdx mice and in ONX‐0914‐treated mdx mice compared to mdx+ABX+ONX mice (Figure S5A,B). This suggests that ONX‐0914 treatment can reduce fibrosis in dystrophic muscles and that this effect is dependent on the gut microbiota. Next, we argued whether the ONX‐0914 treatment modulated the macrophages in skeletal muscle as seen in the colon of mdx mice (Figure 1C,D). We observed a reduction in CD68+ macrophages and a concurrent upregulation of anti‐inflammatory CD206+ macrophages following ONX‐0914 and FTM^mdx+ONX^ treatments but not in mdx+ABX+ONX‐treated mice (Figure 6A,B). RT‐qPCR analysis confirmed a significant downregulation of genes commonly involved in macrophage polarization or inflammatory processes, such as *C‐C Motif Chemokine Ligand 2 (ccl2)* gene and *Vascular Cell Adhesion Molecule 1 (vcam‐1)* gene in ONX‐0914‐treated mdx mice (Figure 6C). This was further supported by proteomic analysis, which revealed a decrease in MCP1, JAK‐1 and MEK‐3 k, macrophage pro‐inflammatory markers, in skeletal muscles of ONX‐0914 and FTM^mdx+ONX^ treated mice compared to untreated mdx and mdx+ABX+ONX mice (Figure S5A,B). We observed a substantial reduction in inflammatory mediators in the TA muscle of mdx mice treated with ONX‐0914 (Figure S5A,B). FTM^mdx+ONX^ mice showed a similar, though less pronounced, effect, particularly with inconsistent NF‐κB changes (Figure S5A,B). In contrast, ONX‐0914‐treated mdx mice showed no significant differences compared to mdx+ABX+ONX mice for IL1β, TNFα and HMGB1 but exhibited a significant increase in IL6 and a significant decrease in these cytokines compared to untreated mdx mice (Figure S5A,B). These findings suggest that ONX‐0914 exerts differential effects on these inflammatory markers partially depending on the presence of the gut microbiota. Next, we assessed the endothelial compartments of treated animals in comparison to C57Bl and untreated mdx mice. ONX‐0914‐ and FTM^mdx+ONX^‐treated mice exhibited a significant downregulation of CD31+, αSMA+ and isolectin+ vessels compared to mdx+ABX+ONX and untreated mdx mice, reaching levels similar to those observed in control C57Bl mice (Figure 6A,D).

## 5 Conclusions

This study builds upon our previous works [19, 20, 27], demonstrating the therapeutic potential of targeting the IP to ameliorate the inflammatory features in mdx mice. Given the established role of the IP in modulating the intestinal defensive barrier and microbiota Supporting Information S1: S21 [28, 29, 30], we explored the effects of IP‐inhibitor ONX‐0914 on the intestinal inflammatory microenvironment and its subsequent impact on muscle pathology in dystrophic mdx mice. ONX‐0914 treatment in mdx mice led to subtle histological changes in the colon, suggesting a mild modulation of intestinal structure. Notably, we observed increased gastrointestinal transit and improved fluid reabsorption, indicating enhanced motor activity within the large intestine. Furthermore, ONX‐0914 partially restored intestinal barrier integrity, as evidenced by reduced FITC‐dextran leakage. The restoration of Lcn2, *IL‐10* and JAM‐A levels—all crucial for intestinal homeostasis—further supports ONX‐0914's positive influence on gut barrier function and reduced inflammation. The observed increase in anti‐inflammatory CD206+ M2 macrophages and decrease in pro‐inflammatory CD68+ M1 macrophages in the colon reinforces these findings. Interestingly, the effects of ONX‐0914 on intestinal targets (including IP, JAM‐A and macrophage polarization) were abrogated by antibiotic‐induced microbiota depletion. This strongly suggests that ONX‐0914 efficacy on intestinal inflammation is dependent on a functional gut microbiota.

Given the established role of microbial‐derived metabolites in regulating macrophage polarization Supporting Information S1: [S22], we hypothesized that ONX‐0914's effects on intestinal barrier integrity and anti‐inflammatory macrophages could be mediated by modulation of the gut microbiota. Metagenomic analysis revealed significant differences in microbiota composition between C57Bl controls and mdx mice, consistent with previous findings [13, 14]. Notably, most differentially abundant species in mdx and mdx+ONX groups were uncharacterized, highlighting the complexity of the mdx gut microbiome and the challenges in functional analysis. While ONX‐0914 did not fully restore the microbiota and SCFA to C57Bl levels, it induced specific changes, including an enrichment of stachyose degradation pathways. Increased stachyose degradation by gut microbiota can lead to galactose and glutathione synthesis Supporting Information S1: [S23]. Metabolomic analysis revealed that ONX‐0914 treatment increased levels of glutathione, galactose, glycerol, glyceraldehyde and TCA cycle intermediates, suggesting a modulation of energy metabolism. In skeletal muscle, ONX‐0914 treatment improved mitochondrial activity, as evidenced by increased expression of ETC complexes and TCA enzymes, which are affected in DMD patients [31] and significantly down‐regulated in DMD animal models [32, 33, 34]. While we cannot exclude the possibility that ONX‐0914 itself could directly influence mitochondrial function Supporting Information S1: [S24]; it is more likely that these effects are mediated by the changes in serum metabolites that follow the treatment. This was further confirmed in FTM^mdx+ONX^‐treated mdx mice but not in mdx+ABX mice, highlighting the microbiota‐dependent nature of ONX‐0914's impact on muscle ATP‐producing mitochondrial machinery involved in regulating the metabolism of fatty acids and glucose in mdx mice [35]. Furthermore, ONX‐0914 and FTM^mdx+ONX^ treatments led to a significant increase in oxidative/glycolytic muscle fibres, a reduction in glycolytic fibres and an increase in SDH+fibres, indicating a shift towards oxidative metabolism. The observed increase in mTOR and its downstream effector 4EBP1/4EBP1p in ONX‐0914 and FTM^mdx+ONX^ treated mice suggests a shift towards anabolic processes in the muscle. mTOR is a central regulator of protein synthesis and muscle hypertrophy, and its activation is often associated with increased muscle growth and repair [36]. This finding aligns with the observed improvement in muscle morphology, with increased fibre size in treated mdx mice.

The enhanced muscle performance, evidenced by increased tetanic muscle force, is a key finding that demonstrates the functional benefits of ONX‐0914 treatment. This improvement likely stems from the combined effects of enhanced mitochondrial activity and metabolic remodelling. The reduction in serum CKs further supports the notion of reduced muscle damage, as CK is a marker of muscle breakdown. Consistently, the modulation of macrophage polarization in skeletal muscle of ONX‐0914 and FTM^mdx+ONX^ treated mice was further supported by proteomic experiments showing a decreased trend of JAK‐1/MCP‐1 and MEK‐3 kinase, which supports the accumulation of pro‐inflammatory M1 macrophages Supporting Information S1: [S25–S27]. We observed a downregulation of *ccl2* and *vcam‐1* genes, which typically mediate a pro‐inflammatory M1 phenotype and macrophage recruitment to inflammatory tissues [37, 38], respectively. Additionally, *Icam‐1*—up‐regulated in M1 macrophages Supporting Information S1: [S28]—was inhibited, while the anti‐inflammatory cytokine *IL‐10* was upregulated, further confirming M2 polarization. ONX‐0914's modulation of the TCA cycle likely contributes to this M2 polarization [39, 40]. Moreover, ONX‐0914 treatment led to a significant decrease in several inflammatory cytokines, including IL1β, TNFα, HMGB1 and IL6, although the effects on NF‐κB were less consistent and appeared to be influenced by the presence of the gut microbiota. The diminished fibrosis observed in ONX‐0914 and FTM^mdx+ONX^ treated mice, along with the decrease in TGFβ levels, suggests that ONX‐0914 may have anti‐fibrotic effects, potentially by modulating inflammatory pathways.

ONX‐0914 and FTM^mdx+ONX^ treatments restored endothelial vessel density to wild‐type levels, indicating reduced muscle damage and diminished capillary remodelling in dystrophic muscle. The absence of ONX‐0914's positive effects on muscle metabolism, function, inflammation and fibrosis in microbiota‐depleted mdx mice (mdx+ABX+ONX) demonstrates the gut microbiota's essential role in these benefits, emphasizing the gut–muscle connection and the potential of gut‐targeted DMD therapies. Detailed investigations into the mechanisms by which ONX‐0914 affects gut microbiota and systemic metabolism are essential for developing targeted DMD therapies, particularly by identifying key microbial taxa and metabolites.

## Ethics Statement

Procedures involving living animals were conformed to Italian law (D.L.vo 116/92) and approved by local ethics committees, following the authorization of the Ministry of Health and Local University of Milan Committee—protocol number 10/13–2014/2015 and 859/2017‐PR (5247B.35, 10/07/2017).

## Conflicts of Interest

The authors declare no conflicts of interest.

## Supporting information


**Figure S 1.** Evaluation of colon morphology in 3 m mdx+ONX‐0914 mice. **(A**) Representative images of H&E staining of colon from 3 m C57Bl, mdx and mdx+ONX mice. High magnification (scale bar: 200 μm) and low magnification (scale bar: 500 μm). (**B**) JAM‐A expression was evaluated in colonic tissues of 3 m C57Bl, mdx, mdx+ABX, mdx+ONX and mdx+ABX+ONX mice (*n* = 5 each, with 5 images analysed per animal). JAM‐A staining is shown in green and DAPI in blue. CD68+ M1 and CD206 + M2 macrophages were quantified in colonic tissues of 3 m C57Bl, mdx, mdx+ABX, mdx+ONX and mdx+ABX+ONX mice (*n* = 5 each, with 5 images analysed per animal). CD206 staining is shown in green, CD68 in red and DAPI in blue. Scale bars: 100 μm. Cropped images of representative WBs show the expression of pro‐inflammatory proteins in macrophages isolated from colon tissues of 3 m C57Bl, mdx and mdx+ONX mice (*n* = 4, two independent experiments) (**C**) and from 3 m C57Bl, mdx, mdx+ABX, mdx+ONX and mdx+ABX+ONX mice (*n* = 3 each, two independent experiments) (**D**). Densitometric analyses of protein expression are shown as a ratio to actin. Data information: data are presented as mean ± SD (*p < 0.05; **p < 0.01, ***p < 0.001, ****p < 0.0001; One‐Way ANOVA Kruskal–Wallis test for evaluation of images and One‐Way ANOVA with Tukey's multiple comparisons test for WB experiments).

**Figure S 2** Microbiota richness is similar between mdx and vehicle‐treated mdx mice. (**A**) Analysis of alpha‐diversity (Wilcoxon sum rank test, *p* = 0.56) as measured by using the total number of observed amplicon sequence variants (ASV) in mdx (CTRL) and vehicle‐treated mdx (VEH) mice (*n* = 4 each). (**B–D**) Analysis of beta‐diversity as measured by using the (**B**) unweighted, (**C**) weighted UniFrac distances and (**D**) Bray–Curtis dissimilarity index (PERMANOVA, *p* > 0.05). (**E**) Stacked barplots representing the relative abundance of the 25 most abundant taxa classified to the genus level per each sample.

**Figure S 3** Gut tissue metabolome profiling in 3‐month‐old C57Bl, mdx and mdx+ONX mice. (**A**) Heatmap showing all the relevant metabolites concentration change among 3 m C57Bl (*n* = 4), mdx (*n* = 4) and mdx+ONX (*n* = 3) with a *p*‐value < 0.05 according to ANOVA. Both metabolites and classes were clusterized according to the Wald method. Bacterial metabolites: 4‐hydroxybutanoic acid, butanoic acid, 5‐keto gluconate and octadecanamide. Metabolites involved in fatty acid synthesis and degradation: dodecanoic acid, palmitic acid, 5‐hydroxyhexanoic acid. Metabolites involved in simple sugar metabolism: glyceraldehyde‐3‐phospahte, fucose, erythrose, erythrose‐4‐phosphate, ribose, arabinose, xylitol, ribitol, mannose, fructose, glucose‐6‐phosphate, glucose, rhamnose, galactitol, mannitol, melibiose, maltose, lactic acid, glycolic acid, oxalic acid. (**B**) Metabolic pathways involving the relevant metabolites obtained using the MetPa algorithm. The colour and size of each circle are based on the *p*‐value and pathway impact value, respectively. The x‐axis represents the pathway impact, and the y‐axis represents the −log of *p* values from the pathway enrichment analysis for the key differential metabolites of 3 m C57Bl, mdx and mdx+ONX.

**Figure S 4** Mitochondrial enzymes activity in muscles of 3 m mdx+ONX‐0914 mice. Enzymatic activity of mitochondrial enzymes involved in respiratory chain complexes of TA and DIA of 3 m C57Bl, mdx and mdx+ONX mice (*n* = 3 each). The following abbreviations were used in the picture (NADH DH/citr synt: NADH dehydrogenase/citrate synthase; NADH ubiq 1 red/cit synt: NADH ubiquinone 1 reductase/citrate synthase; succinate DH/citr synt: succinate dehydrogenase/citrate synthase; succinate CoQ red/citr synt: succinate CoQ reductase/citrate synthase; cytr ox/citr synt: cytochrome oxidase/citrate synthase; NADH cit C red/citr synt: NADH citrate C reductase/citrate synthase; and succinate cit C red/citr synt: succinate citrate C reductase/citrate synthase). Data information: data are presented as mean ± SD (*p < 0.05; **p < 0.01, ***p < 0.001; One‐Way ANOVA with Tukey's multiple comparisons test).

**Figure S 5** Proteomic evaluation of microbiota‐depleted ONX‐treated skeletal muscles. Cropped images of representative WB analysis of TA muscle of (**A**) 3 m C57Bl and mdx (*n* = 3 each), mdx^Fmdx^ and mdx^Fmdx+ONX^ (*n* = 4 each) mice and of (**B**) 3 m mdx, mdx+ABX, mdx+ONX and mdx+ABX+ONX mice (*n* = 3 each, two independent experiments) showing the expression of the proteins specifically involved in mitochondrial functions and TCA complex; OXPHOS complex (C1: NDUFB8; C2: SDHB; C3: UQCRC2; C4: MTCO1; C5: ATP5A); mTOR‐dependent pathways; M1‐ and M2‐macrophages proliferation, skeletal muscle metabolism and pro‐inflammatory cytokines. OGDH: Oxoglutarate Dehydrogenase; CS: Citrate Synthase; MDH2: Malate Dehydrogenase 2; FH: Fumarate Hydratase; ACO2: Aconitase 2; IDH2: Isocitrate Dehydrogenase NADP(+) 2. Data information: densitometric data were normalized on vinculin and expressed as mean ± SD (*p < 0.05, **p < 0.01, ***p < 0.001; ****p < 0.0001, ordinary one‐way ANOVA, Tuckey multiple comparison test).

**Figure S 6** Representative staining of muscles following ONX‐0914 treatment and gut microbiota modulation. Representative staining of muscle sections expressing different MyHC isoforms (Type IIa in green, IIx in black, IIb in red; Type I in blu) in TAs of C57Bl, mdx, mdx+ABX, mdx+ONX and mdx+ABX+ONX mice and mdx^Fmdx^ and mdx^Fmdx+ONX^ mice (*n* = 3 each and *n* = 10 images per animal) (two independent experiments). Scale bar: 100 μm. Representative SDH staining of TAs from C57Bl, mdx, mdx+ABX, mdx+ONX and mdx+ABX+ONX mice (*n* = 5 each and *n* = 8 images per animal) and mdx^Fmdx^ and mdx^Fmdx+ONX^ mice (*n* = 4 each and *n* = 10 images per animal) (two independent experiments); EE staining of C57Bl, mdx, mdx+ABX, mdx+ONX and mdx+ABX+ONX mice and mdx^Fmdx^ and mdx^Fmdx+ONX^ mice (*n* = 4 each); Syrius Red staining of TAs from C57Bl, mdx, mdx+ABX, mdx+ONX and mdx+ABX+ONX mice (*n* = 3 each and *n* = 10 images per animal) and mdx^Fmdx^ and mdx^Fmdx+ONX^ mice (*n* = 3 each and *n* = 10 images per animal) (two independent experiments). Scale bar: 200 μm.

**Figure S 7** Food and water consumption of ONX‐0914‐treated mdx mice. Food and water consumption calculated on the weight of each animal in 3 m C57Bl (*n* = 6) (in red), mdx (*n* = 7) (in green) and mdx + ONX (*n* = 8) (in blue).

**Data S1:** Supplementary information.

**Data S2:** Supplementary information.

## Data Availability

Source data for all main figures and extended data figures are supplied with this paper. All necessary data to evaluate the paper's conclusions are available in the paper and Supporting Information. Other experimental data supporting the plots within this paper and other findings of this study are available from the corresponding author upon reasonable request.

## References

[1] G. Bulfield , W. G. Siller , P. A. Wight , and K. J. Moore , “X Chromosome‐Linked Muscular Dystrophy (mdx) in the Mouse,” Proceedings of the National Academy of Sciences of the United States of America 81 (1984): 1189–1192. 6583703 10.1073/pnas.81.4.1189 PMC344791

[2] W. Duddy , S. Duguez , H. Johnston , et al., “Muscular Dystrophy in the Mdx Mouse is a Severe Myopathy Compounded by Hypotrophy, Hypertrophy and Hyperplasia,” Skeletal Muscle 5 (2015): 16. 25987977 10.1186/s13395-015-0041-y PMC4434871

[3] A. Briguet , I. Courdier‐Fruh , M. Foster , T. Meier , and J. P. Magyar , “Histological Parameters for the Quantitative Assessment of Muscular Dystrophy in the Mdx‐Mouse,” Neuromuscular Disorders 14 (2004): 675–682. 15351425 10.1016/j.nmd.2004.06.008

[4] B. Chazaud , “Inflammation and Skeletal Muscle Regeneration: Leave It to the Macrophages!,” Trends in Immunology 41 (2020): 481–492. 32362490 10.1016/j.it.2020.04.006

[5] S. A. Villalta , A. S. Rosenberg , and J. A. Bluestone , “The Immune System in Duchenne Muscular Dystrophy: Friend or Foe,” Rare Diseases 3 (2015): e1010966. 26481612 10.1080/21675511.2015.1010966 PMC4588155

[6] C. M. Lo Cascio , O. Goetze , T. D. Latshang , S. Bluemel , T. Frauenfelder , and K. E. Bloch , “Gastrointestinal Dysfunction in Patients With Duchenne Muscular Dystrophy,” PLoS ONE 11 (2016): e0163779. 27736891 10.1371/journal.pone.0163779 PMC5063332

[7] K. Singh , G. Randhwa , F. N. Salloum , J. R. Grider , and K. S. Murthy , “Decreased Smooth Muscle Function, Peristaltic Activity, and Gastrointestinal Transit in Dystrophic (mdx) Mice,” Neurogastroenterology and Motility 33 (2021): e13968. 32789934 10.1111/nmo.13968

[8] J. B. Lynch and E. Y. Hsiao , “Microbiomes as Sources of Emergent Host Phenotypes,” Science 365 (2019): 1405–1409. 31604267 10.1126/science.aay0240

[9] D. Zheng , T. Liwinski , and E. Elinav , “Interaction Between Microbiota and Immunity in Health and Disease,” Cell Research 30 (2020): 492–506. 32433595 10.1038/s41422-020-0332-7 PMC7264227

[10] Y. Belkaid and T. W. Hand , “Role of the Microbiota in Immunity and Inflammation,” Cell 157 (2014): 121–141. 24679531 10.1016/j.cell.2014.03.011 PMC4056765

[11] Y. Belkaid and S. Naik , “Compartmentalized and Systemic Control of Tissue Immunity by Commensals,” Nature Immunology 14 (2013): 646–653. 23778791 10.1038/ni.2604 PMC3845005

[12] T. Ichinohe , I. K. Pang , Y. Kumamoto , et al., “Microbiota Regulates Immune Defense Against Respiratory Tract Influenza A Virus Infection,” Proceedings of the National Academy of Sciences of the United States of America 108 (2011): 5354–5359. 21402903 10.1073/pnas.1019378108 PMC3069176

[13] A. Farini , L. Tripodi , C. Villa , et al., “Microbiota Dysbiosis Influences Immune System and Muscle Pathophysiology of Dystrophin‐Deficient Mice,” EMBO Molecular Medicine 15 (2023): e16244. 36533294 10.15252/emmm.202216244 PMC9994487

[14] H. Kalkan , E. Pagano , D. Paris , et al., “Targeting Gut Dysbiosis Against Inflammation and Impaired Autophagy in Duchenne Muscular Dystrophy,” EMBO Molecular Medicine 15 (2023): e16225. 36594243 10.15252/emmm.202216225 PMC9994484

[15] T. Liu , L. Zhang , D. Joo , and S. C. Sun , “NF‐kappaB Signaling in Inflammation,” Signal Transduction and Targeted Therapy 2 (2017): 17023. 29158945 10.1038/sigtrans.2017.23 PMC5661633

[16] F. Gizard , A. Fernandez , and F. De Vadder , “Interactions Between Gut Microbiota and Skeletal Muscle,” Nutrition and Metabolic Insights 13 (2020): 1178638820980490. 33402830 10.1177/1178638820980490 PMC7745561

[17] S. Lahiri , H. Kim , I. Garcia‐Perez , et al., “The Gut Microbiota Influences Skeletal Muscle Mass and Function in Mice,” Science Translational Medicine 11 (2019): eaan5662. 31341063 10.1126/scitranslmed.aan5662 PMC7501733

[18] H. J. Kim , Y. J. Kim , Y. J. Kim , et al., “Microbiota Influences Host Exercise Capacity via Modulation of Skeletal Muscle Glucose Metabolism in Mice,” Experimental & Molecular Medicine 55 (2023): 1820–1830. 37542180 10.1038/s12276-023-01063-4 PMC10474268

[19] A. Farini , A. Gowran , P. Bella , et al., “Fibrosis Rescue Improves Cardiac Function in Dystrophin‐Deficient Mice and Duchenne Patient‐Specific Cardiomyocytes by Immunoproteasome Modulation,” American Journal of Pathology 189 (2019): 339–353. 30448404 10.1016/j.ajpath.2018.10.010

[20] A. Farini , C. Sitzia , B. Cassani , et al., “Therapeutic Potential of Immunoproteasome Inhibition in Duchenne Muscular Dystrophy,” Molecular Therapy 24 (2016): 1898–1912. 27506451 10.1038/mt.2016.162 PMC5154478

[21] A. Farini , L. Tripodi , C. Villa , et al., “Inhibition of the Immunoproteasome Modulates Innate Immunity to Ameliorate Muscle Pathology of Dysferlin‐Deficient BlAJ Mice,” Cell Death & Disease 13 (2022): 975. 36402750 10.1038/s41419-022-05416-1 PMC9675822

[22] D. E. Wood , J. Lu , and B. Langmead , “Improved Metagenomic Analysis With Kraken 2,” Genome Biology 20 (2019): 257. 31779668 10.1186/s13059-019-1891-0 PMC6883579

[23] C. M. Ferreira , A. T. Vieira , M. A. R. Vinolo , F. A. Oliveira , R. Curi , and F. d. S. Martins , “The Central Role of the Gut Microbiota in Chronic Inflammatory Diseases,” Journal of Immunology Research 2014 (2014): 18;2014:689492. 10.1155/2014/689492 PMC4189530 25309932

[24] S. Lobionda , P. Sittipo , H. Y. Kwon , and Y. K. Lee , “The Role of Gut Microbiota in Intestinal Inflammation With Respect to Diet and Extrinsic Stressors,” Microorganisms 7, no. 8 (2019): 271. 31430948 10.3390/microorganisms7080271 PMC6722800

[25] Z. Al Bander , M. D. Nitert , A. Mousa , and N. Naderpoor , “The Gut Microbiota and Inflammation: An Overview,” International Journal of Environmental Research and Public Health 17, no. 20 (2020): 7618. 33086688 10.3390/ijerph17207618 PMC7589951

[26] E. Pasolli , F. Asnicar , S. Manara , et al., “Extensive Unexplored Human Microbiome Diversity Revealed by Over 150,000 Genomes From Metagenomes Spanning Age, Geography, and Lifestyle,” Cell 176 (2019): e20. 10.1016/j.cell.2019.01.001 PMC6349461 30661755

[27] L. Tripodi , D. Molinaro , F. Fortunato , et al., “Immunoproteasome Inhibition Ameliorates Aged Dystrophic Mouse Muscle Environment,” International Journal of Molecular Sciences 23 (2022): 14657. 36498987 10.3390/ijms232314657 PMC9739773

[28] A. Goichon , W. Bahlouli , I. Ghouzali , et al., “Colonic Proteome Signature in Immunoproteasome‐Deficient Stressed Mice and Its Relevance for Irritable Bowel Syndrome,” Journal of Proteome Research 18, no. 1 (2019): 478–492. 30475625 10.1021/acs.jproteome.8b00793

[29] G. Mohapatra , A. Eisenberg‐Lerner , and Y. Merbl , “Gatekeepers of the Gut: The Roles of Proteasomes at the Gastrointestinal Barrier,” Biomolecules 11 (2021): 989. 34356615 10.3390/biom11070989 PMC8301830

[30] M. Guardamagna , M. A. Berciano‐Guerrero , R. Lavado‐Valenzuela , et al., “Association of Gut Microbiota and Immune Gene Expression With Response to Targeted Therapy in BRAF Mutated Melanoma,” Scientific Reports 15 (2025): 25430. 40659866 10.1038/s41598-025-11054-2 PMC12259845

[31] C. A. Timpani , A. Hayes , and E. Rybalka , “Revisiting the Dystrophin‐ATP Connection: How Half a Century of Research Still Implicates Mitochondrial Dysfunction in Duchenne Muscular Dystrophy Aetiology,” Medical Hypotheses 85 (2015): 1021–1033. 26365249 10.1016/j.mehy.2015.08.015

[32] M. Khairallah , R. Khairallah , M. E. Young , J. R. Dyck , B. J. Petrof , and C. Des Rosiers , “Metabolic and Signaling Alterations in Dystrophin‐Deficient Hearts Precede Overt Cardiomyopathy,” Journal of Molecular and Cellular Cardiology 43 (2007): 119–129. 17583724 10.1016/j.yjmcc.2007.05.015

[33] A. Lindsay , C. M. Chamberlain , B. A. Witthuhn , D. A. Lowe , and J. M. Ervasti , “Dystrophinopathy‐Associated Dysfunction of Krebs Cycle Metabolism,” Human Molecular Genetics 28 (2019): 942–951. 30476171 10.1093/hmg/ddy404 PMC6400043

[34] L. W. Markham , C. L. Brinkmeyer‐Langford , J. H. Soslow , et al., “GRMD Cardiac and Skeletal Muscle Metabolism Gene Profiles Are Distinct,” BMC Medical Genomics 10 (2017): 21. 28390424 10.1186/s12920-017-0257-2 PMC5385041

[35] M. F. Goody and C. A. Henry , “A Need for NAD^+^ in Muscle Development, Homeostasis, and Aging,” Skeletal Muscle 8 (2018): 9. 29514713 10.1186/s13395-018-0154-1 PMC5840929

[36] M. Laplante and D. M. Sabatini , “mTOR Signaling in Growth Control and Disease,” Cell 149 (2012): 274–293. 22500797 10.1016/j.cell.2012.03.017 PMC3331679

[37] E. Sierra‐Filardi , C. Nieto , A. Dominguez‐Soto , et al., “CCL2 Shapes Macrophage Polarization by GM‐CSF and M‐CSF: Identification of CCL2/CCR2‐Dependent Gene Expression Profile,” Journal of Immunology 192 (2014): 3858–3867. 10.4049/jimmunol.1302821 24639350

[38] D. H. Kong , Y. K. Kim , M. R. Kim , J. H. Jang , and S. Lee , “Emerging Roles of Vascular Cell Adhesion Molecule‐1 (VCAM‐1) in Immunological Disorders and Cancer,” International Journal of Molecular Sciences 19 (2018): 1057. 29614819 10.3390/ijms19041057 PMC5979609

[39] A. K. Jha , S. C. Huang , A. Sergushichev , et al., “Network Integration of Parallel Metabolic and Transcriptional Data Reveals Metabolic Modules That Regulate Macrophage Polarization,” Immunity 42 (2015): 419–430. 25786174 10.1016/j.immuni.2015.02.005

[40] E. L. Mills and L. A. O'Neill , “Reprogramming Mitochondrial Metabolism in Macrophages as an Anti‐Inflammatory Signal,” European Journal of Immunology 46 (2016): 13–21. 26643360 10.1002/eji.201445427

