# Supplementary figures and images for: Immunoproteasome Inhibition Positively Impacts the Gut‐Muscle Axis in Duchenne Muscular Dystrophy
Source: J Cachexia Sarcopenia Muscle. 2025 Oct 1;16(5):e70054. doi: 10.1002/jcsm.70054 (PMC12489020; doi:10.1002/jcsm.70054)

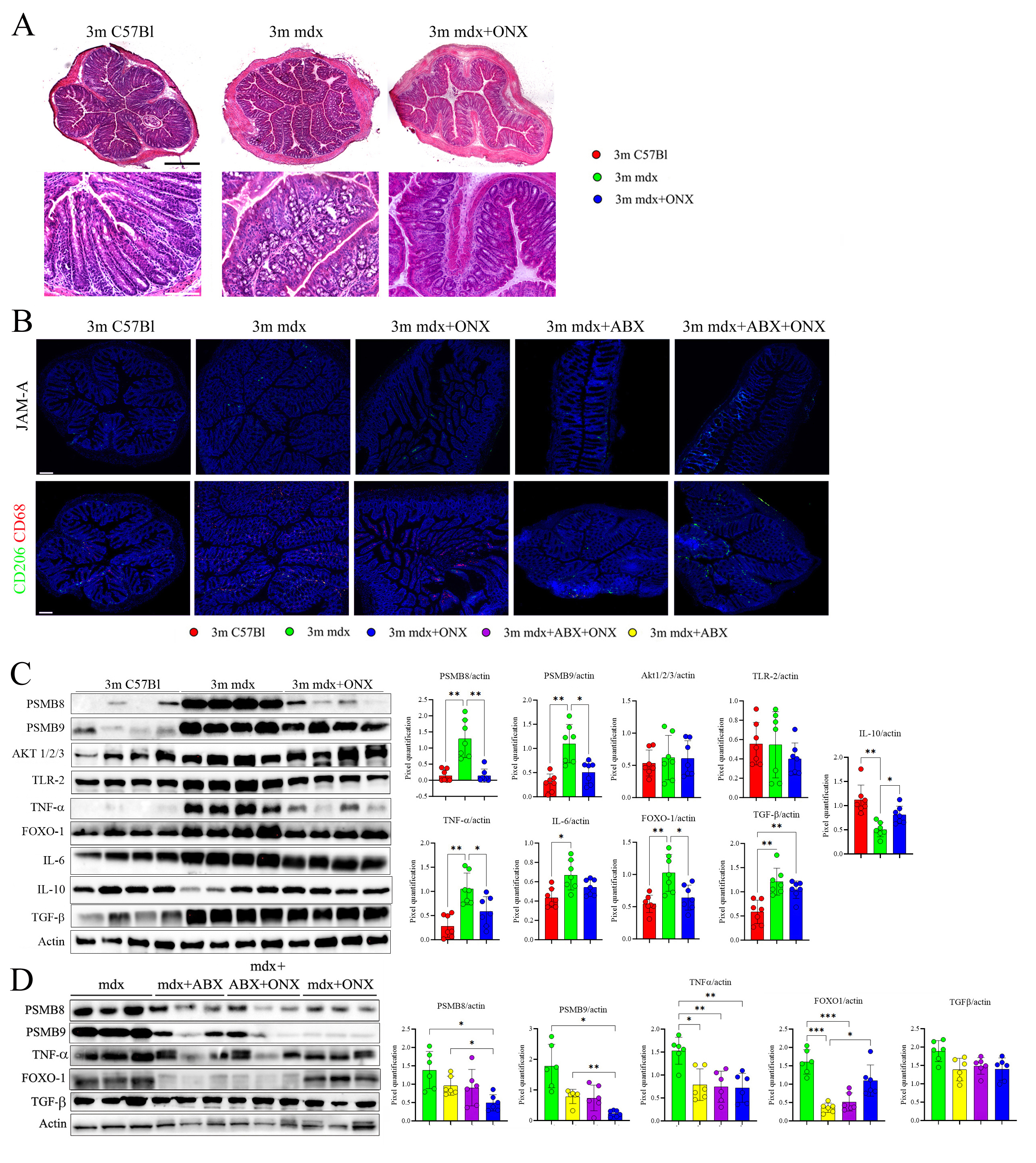

Supplement: Supplementary file 1 — Figure S 1. Evaluation of colon morphology in 3 m mdx+ONX‐0914 mice. (A) Representative images of H&E staining of colon from 3 m C57Bl, mdx and mdx+ONX mice. High magnification (scale bar: 200 μm) and low magnification (scale bar: 500 μm). (B) JAM‐A expression was evaluated in colonic tissues of 3 m C57Bl, mdx, mdx+ABX, mdx+ONX and mdx+ABX+ONX mice (n = 5 each, with 5 images analysed per animal). JAM‐A staining is shown in green and DAPI in blue. CD68+ M1 and CD206 + M2 macrophages were quantified in colonic tissues of 3 m C57Bl, mdx, mdx+ABX, mdx+ONX and mdx+ABX+ONX mice (n = 5 each, with 5 images analysed per animal). CD206 staining is shown in green, CD68 in red and DAPI in blue. Scale bars: 100 μm. Cropped images of representative WBs show the expression of pro‐inflammatory proteins in macrophages isolated from colon tissues of 3 m C57Bl, mdx and mdx+ONX mice (n = 4, two independent experiments) (C) and from 3 m C57Bl, mdx, mdx+ABX, mdx+ONX and mdx+ABX+ONX mice (n = 3 each, two independent experiments) (D). Densitometric analyses of protein expression are shown as a ratio to actin. Data information: data are presented as mean ± SD (*p < 0.05; **p < 0.01, ***p < 0.001, ****p < 0.0001; One‐Way ANOVA Kruskal–Wallis test for evaluation of images and One‐Way ANOVA with Tukey's multiple comparisons test for WB experiments). [file JCSM-16-e70054-s003.jpg]

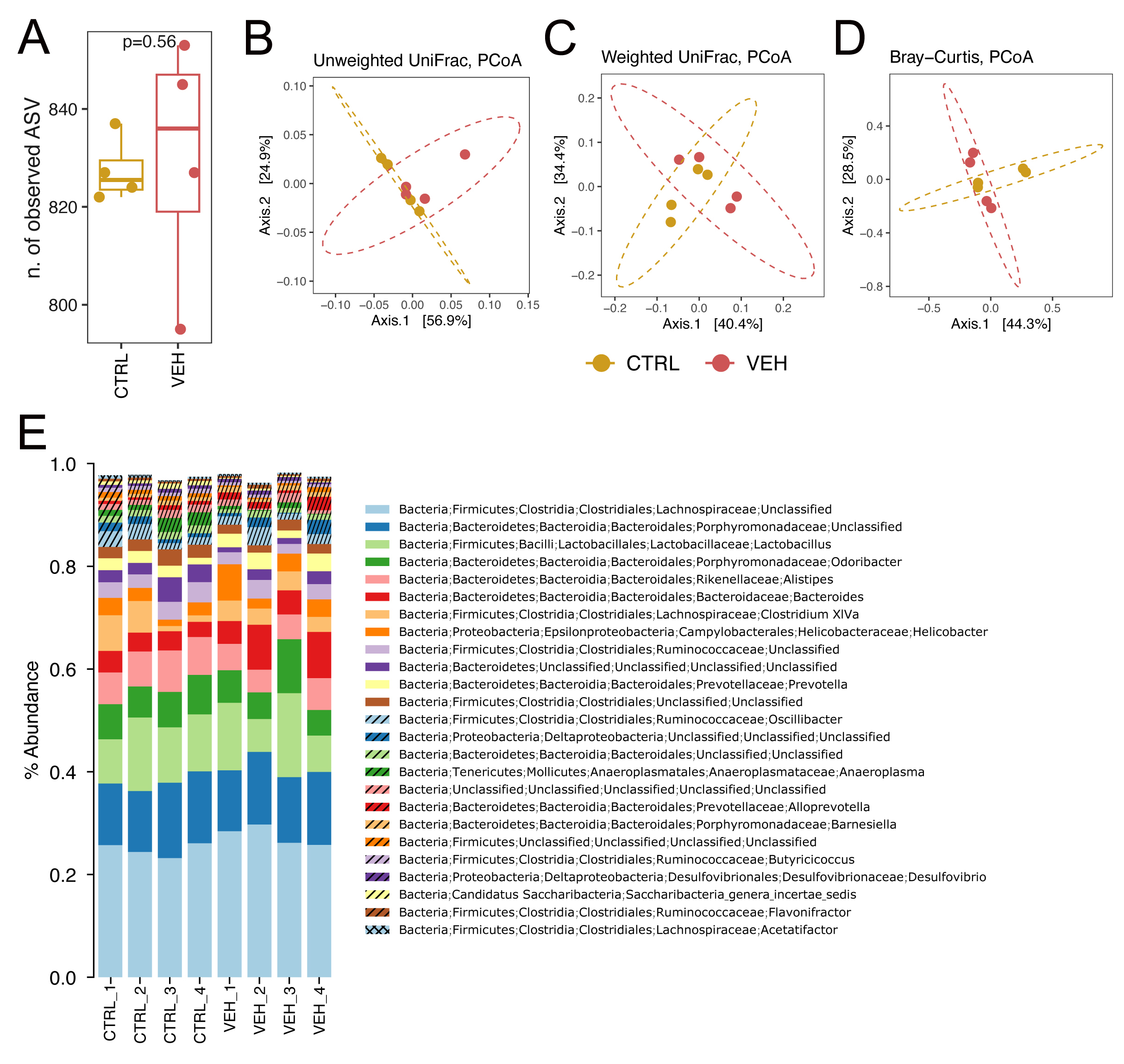

Supplement: Supplementary file 2 — Figure S 2 Microbiota richness is similar between mdx and vehicle‐treated mdx mice. (A) Analysis of alpha‐diversity (Wilcoxon sum rank test, p = 0.56) as measured by using the total number of observed amplicon sequence variants (ASV) in mdx (CTRL) and vehicle‐treated mdx (VEH) mice (n = 4 each). (B–D) Analysis of beta‐diversity as measured by using the (B) unweighted, (C) weighted UniFrac distances and (D) Bray–Curtis dissimilarity index (PERMANOVA, p > 0.05). (E) Stacked barplots representing the relative abundance of the 25 most abundant taxa classified to the genus level per each sample. [file JCSM-16-e70054-s007.jpg]

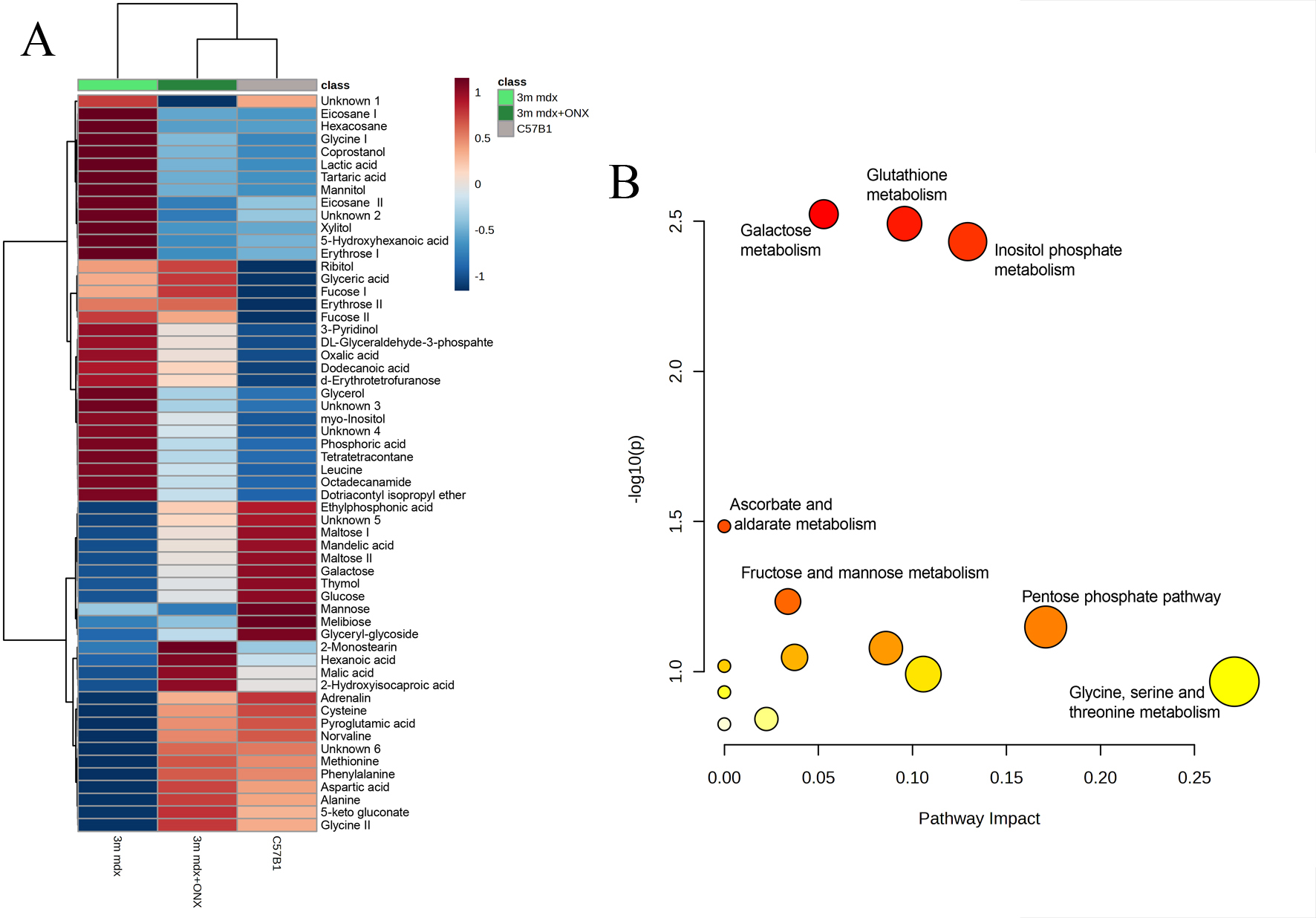

Supplement: Supplementary file 3 — Figure S 3 Gut tissue metabolome profiling in 3‐month‐old C57Bl, mdx and mdx+ONX mice. (A) Heatmap showing all the relevant metabolites concentration change among 3 m C57Bl (n = 4), mdx (n = 4) and mdx+ONX (n = 3) with a p‐value < 0.05 according to ANOVA. Both metabolites and classes were clusterized according to the Wald method. Bacterial metabolites: 4‐hydroxybutanoic acid, butanoic acid, 5‐keto gluconate and octadecanamide. Metabolites involved in fatty acid synthesis and degradation: dodecanoic acid, palmitic acid, 5‐hydroxyhexanoic acid. Metabolites involved in simple sugar metabolism: glyceraldehyde‐3‐phospahte, fucose, erythrose, erythrose‐4‐phosphate, ribose, arabinose, xylitol, ribitol, mannose, fructose, glucose‐6‐phosphate, glucose, rhamnose, galactitol, mannitol, melibiose, maltose, lactic acid, glycolic acid, oxalic acid. (B) Metabolic pathways involving the relevant metabolites obtained using the MetPa algorithm. The colour and size of each circle are based on the p‐value and pathway impact value, respectively. The x‐axis represents the pathway impact, and the y‐axis represents the −log of p values from the pathway enrichment analysis for the key differential metabolites of 3 m C57Bl, mdx and mdx+ONX. [file JCSM-16-e70054-s005.jpg]

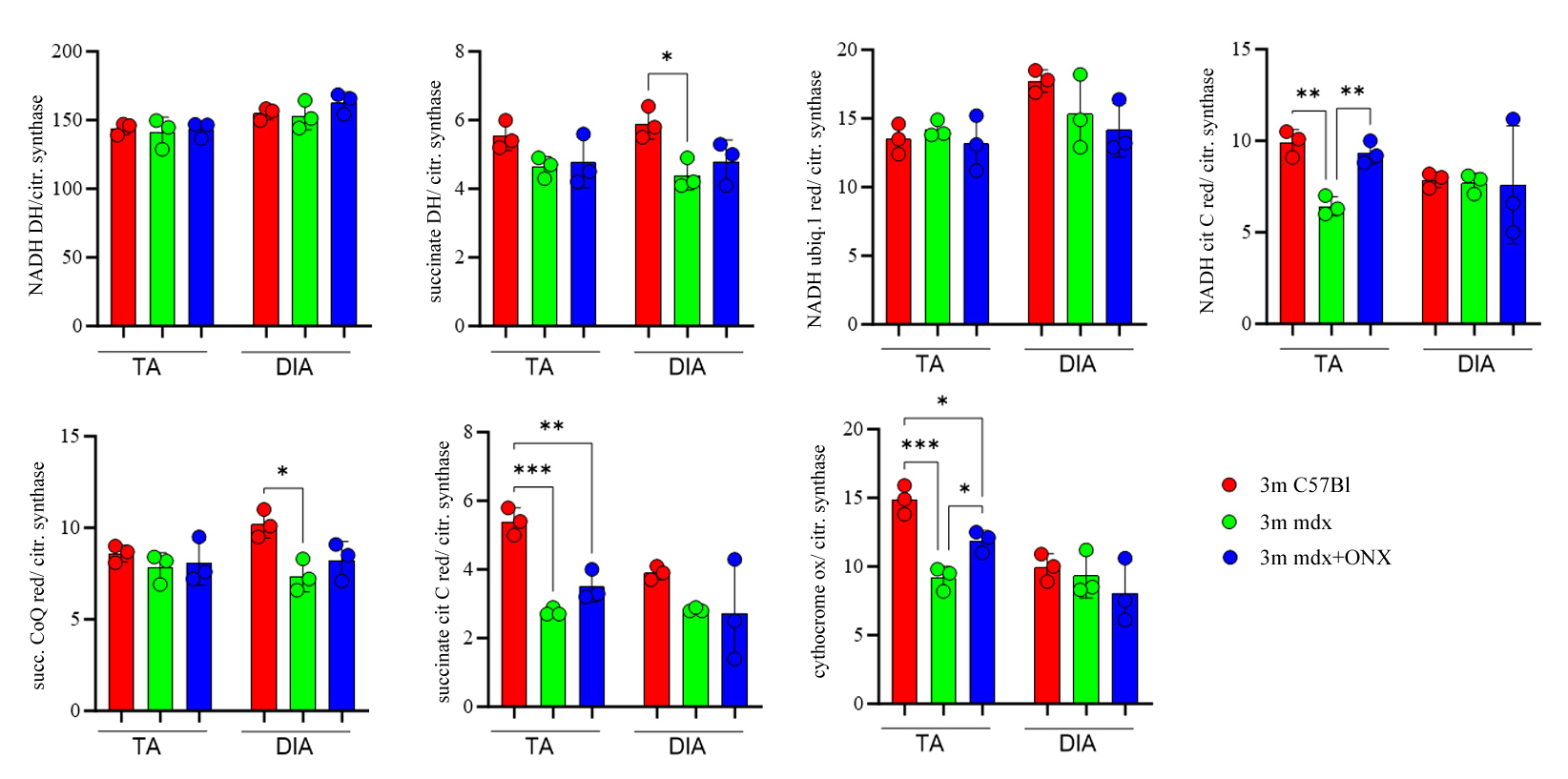

Supplement: Supplementary file 4 — Figure S 4 Mitochondrial enzymes activity in muscles of 3 m mdx+ONX‐0914 mice. Enzymatic activity of mitochondrial enzymes involved in respiratory chain complexes of TA and DIA of 3 m C57Bl, mdx and mdx+ONX mice (n = 3 each). The following abbreviations were used in the picture (NADH DH/citr synt: NADH dehydrogenase/citrate synthase; NADH ubiq 1 red/cit synt: NADH ubiquinone 1 reductase/citrate synthase; succinate DH/citr synt: succinate dehydrogenase/citrate synthase; succinate CoQ red/citr synt: succinate CoQ reductase/citrate synthase; cytr ox/citr synt: cytochrome oxidase/citrate synthase; NADH cit C red/citr synt: NADH citrate C reductase/citrate synthase; and succinate cit C red/citr synt: succinate citrate C reductase/citrate synthase). Data information: data are presented as mean ± SD (*p < 0.05; **p < 0.01, ***p < 0.001; One‐Way ANOVA with Tukey's multiple comparisons test). [file JCSM-16-e70054-s006.jpg]

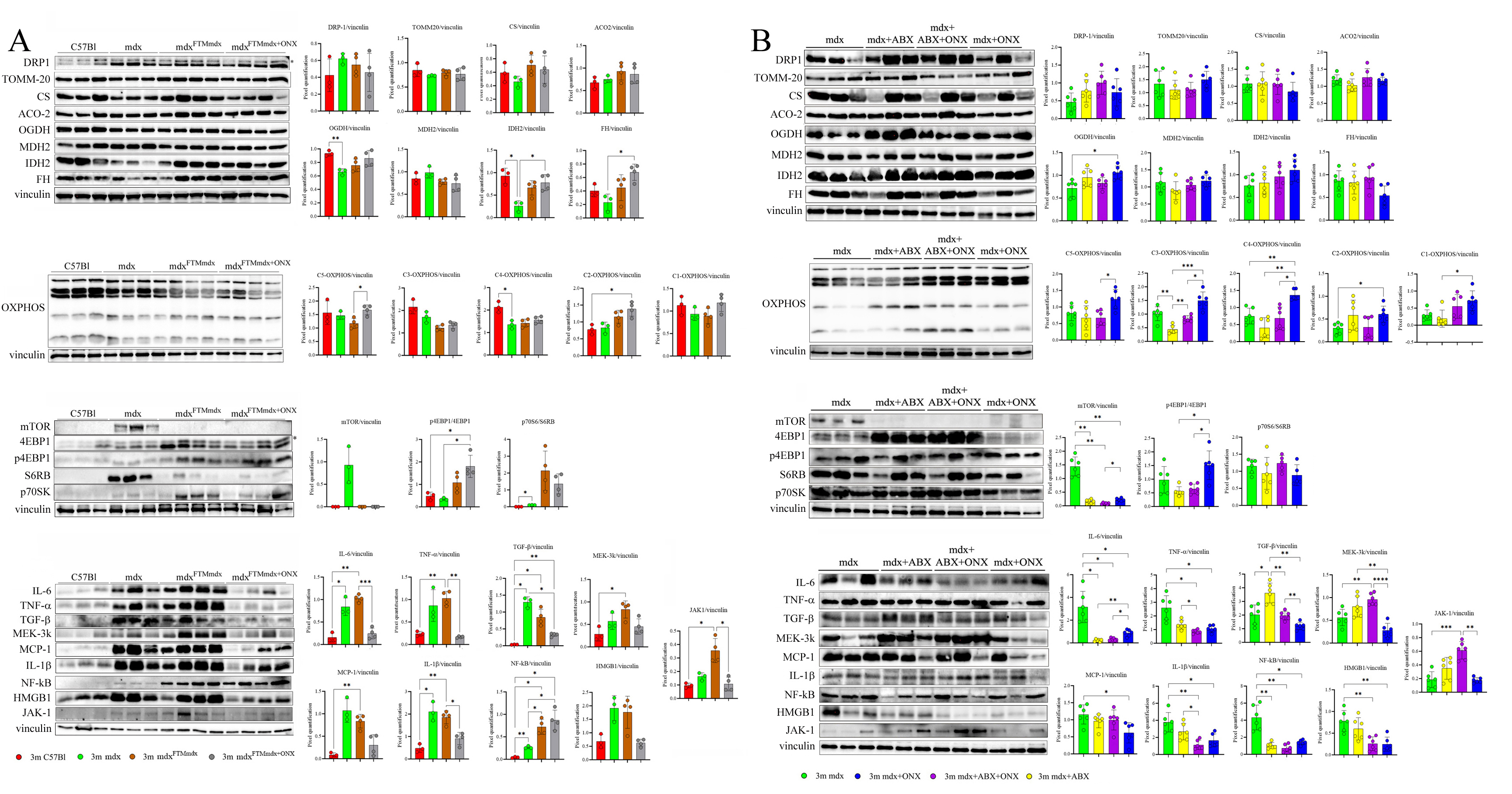

Supplement: Supplementary file 5 — Figure S 5 Proteomic evaluation of microbiota‐depleted ONX‐treated skeletal muscles. Cropped images of representative WB analysis of TA muscle of (A) 3 m C57Bl and mdx (n = 3 each), mdxFmdx and mdxFmdx+ONX (n = 4 each) mice and of (B) 3 m mdx, mdx+ABX, mdx+ONX and mdx+ABX+ONX mice (n = 3 each, two independent experiments) showing the expression of the proteins specifically involved in mitochondrial functions and TCA complex; OXPHOS complex (C1: NDUFB8; C2: SDHB; C3: UQCRC2; C4: MTCO1; C5: ATP5A); mTOR‐dependent pathways; M1‐ and M2‐macrophages proliferation, skeletal muscle metabolism and pro‐inflammatory cytokines. OGDH: Oxoglutarate Dehydrogenase; CS: Citrate Synthase; MDH2: Malate Dehydrogenase 2; FH: Fumarate Hydratase; ACO2: Aconitase 2; IDH2: Isocitrate Dehydrogenase NADP(+) 2. Data information: densitometric data were normalized on vinculin and expressed as mean ± SD (*p < 0.05, **p < 0.01, ***p < 0.001; ****p < 0.0001, ordinary one‐way ANOVA, Tuckey multiple comparison test). [file JCSM-16-e70054-s004.jpg]

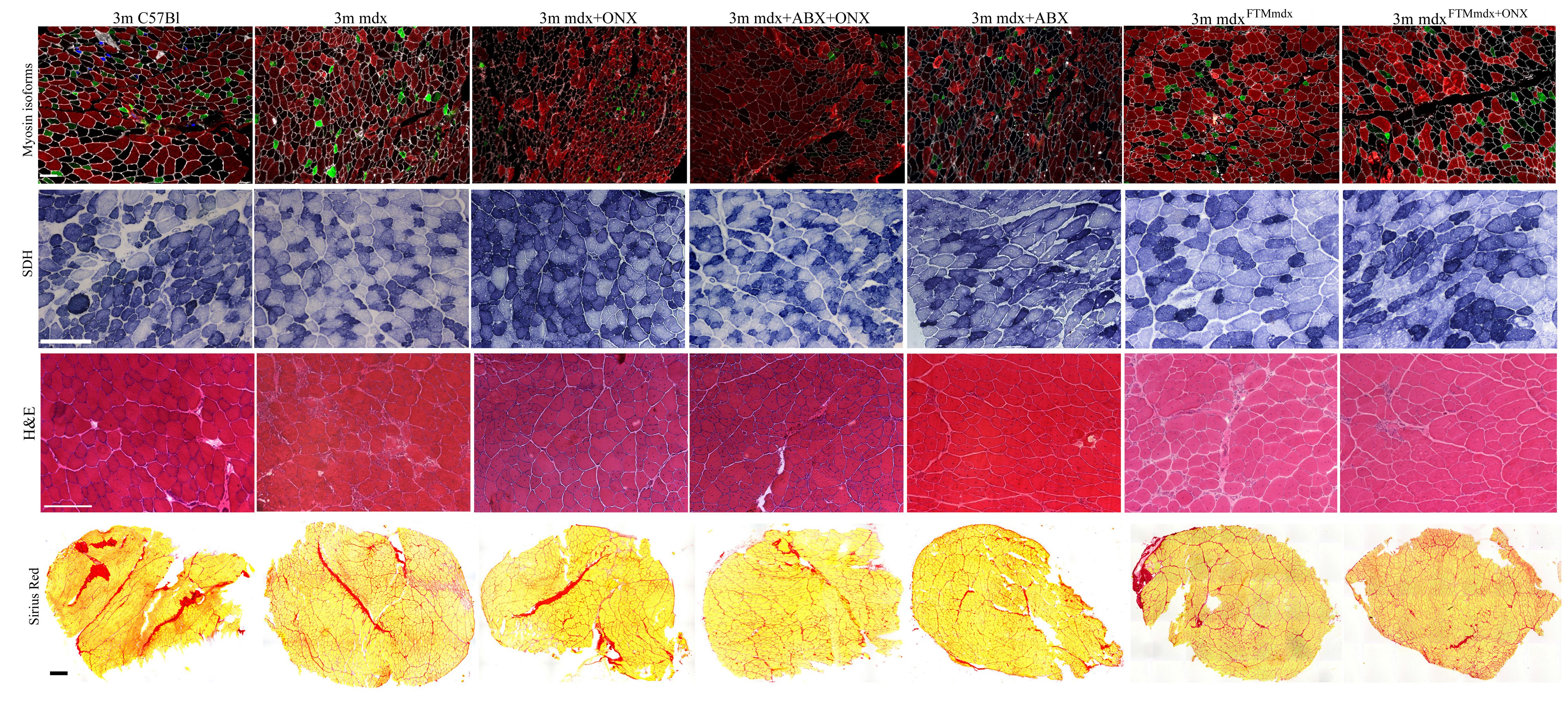

Supplement: Supplementary file 6 — Figure S 6 Representative staining of muscles following ONX‐0914 treatment and gut microbiota modulation. Representative staining of muscle sections expressing different MyHC isoforms (Type IIa in green, IIx in black, IIb in red; Type I in blu) in TAs of C57Bl, mdx, mdx+ABX, mdx+ONX and mdx+ABX+ONX mice and mdxFmdx and mdxFmdx+ONX mice (n = 3 each and n = 10 images per animal) (two independent experiments). Scale bar: 100 μm. Representative SDH staining of TAs from C57Bl, mdx, mdx+ABX, mdx+ONX and mdx+ABX+ONX mice (n = 5 each and n = 8 images per animal) and mdxFmdx and mdxFmdx+ONX mice (n = 4 each and n = 10 images per animal) (two independent experiments); EE staining of C57Bl, mdx, mdx+ABX, mdx+ONX and mdx+ABX+ONX mice and mdxFmdx and mdxFmdx+ONX mice (n = 4 each); Syrius Red staining of TAs from C57Bl, mdx, mdx+ABX, mdx+ONX and mdx+ABX+ONX mice (n = 3 each and n = 10 images per animal) and mdxFmdx and mdxFmdx+ONX mice (n = 3 each and n = 10 images per animal) (two independent experiments). Scale bar: 200 μm. [file JCSM-16-e70054-s002.jpg]

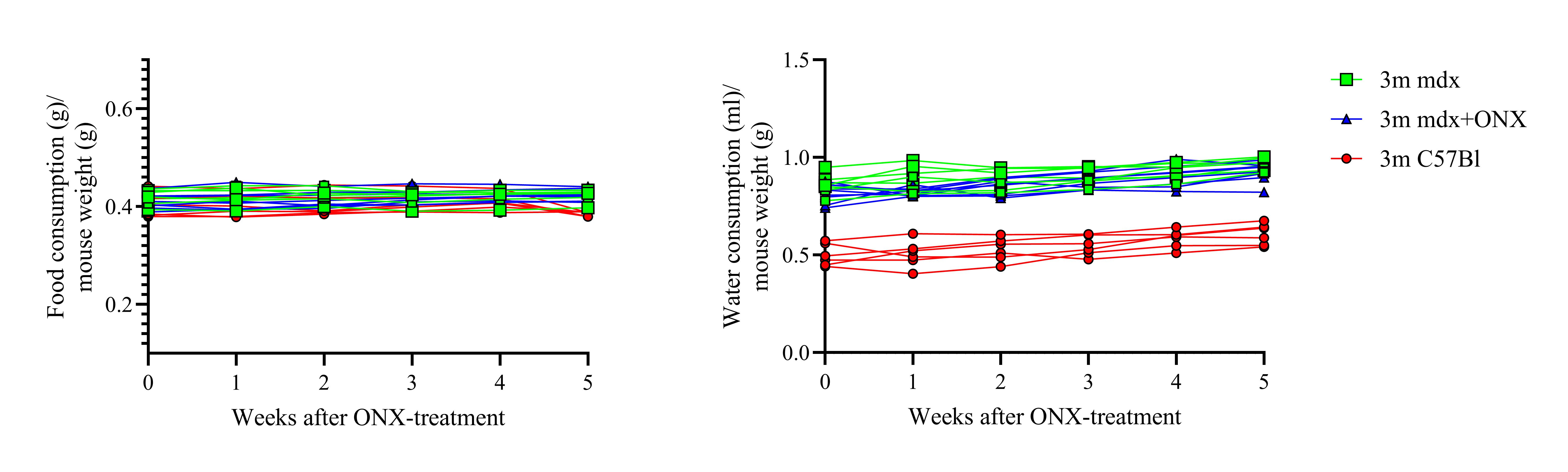

Supplement: Supplementary file 7 — Figure S 7 Food and water consumption of ONX‐0914‐treated mdx mice. Food and water consumption calculated on the weight of each animal in 3 m C57Bl (n = 6) (in red), mdx (n = 7) (in green) and mdx + ONX (n = 8) (in blue). [file JCSM-16-e70054-s009.jpg]
