# Supplementary material for: Immunoproteasome Inhibition Positively Impacts the Gut‐Muscle Axis in Duchenne Muscular Dystrophy
Source: J Cachexia Sarcopenia Muscle. 2025 Oct 1;16(5):e70054. doi: 10.1002/jcsm.70054 (PMC12489020; doi:10.1002/jcsm.70054)
Supplement: Supplementary file 8 — Data S1: Supplementary information. [file JCSM-16-e70054-s008.docx]

**Immunoproteasome inhibition positively impacts the gut-muscle axis in Duchenne muscular dystrophy.**

Andrea Farini ^1^, Francesco Strati ^2^, Monica Molinaro ^1^, Debora Mostosi ^3^, Sabrina Saccone ^1^, Luana Tripodi ^3^, Jacopo Troisi ^4,5^, Annamaria Landolfi ^4,5^, Chiara Amoroso ^2^, Barbara Cassani ^6^, Aitor Blanco-Míguez ^7^, Emma Leonetti ^3^, Davide Bazzani ^7^, Mattia Bolzan ^7^, Francesco Fortunato ^8^, Flavio Caprioli ^9^, Federica Facciotti ^2^, Yvan Torrente ^# 1,3^.

1 Neurology Unit, Fondazione IRCCS Ca' Granda Ospedale Maggiore Policlinico, Milan, Italy

2 Department of Biotechnology and Biosciences, University of Milano-Bicocca, Milan, Italy

3 Stem Cell Laboratory, Dino Ferrari Center, Department of Pathophysiology and Transplantation, University of Milan, Milan, Italy

4 Department of Medicine, Surgery and Dentistry, Scuola Medica Salernitana, University of Salerno, 84081, Baronissi, Italy

5 Theoreo srl, spinoff company of the University of Salerno, Montecorvino Pugliano, Italy

6 Department of Medical Biotechnologies and Translational Medicine, Università Degli Studi di Milano, Milan, Italy

7 PreBiomics S.r.l., Trento, Italy

8 Dino Ferrari Centre, Department of Pathophysiology and Transplantation (DEPT), University of Milan, 20122 Milan, Italy.

9 Department of Pathophysiology and Transplantation, Università degli Studi di Milano, Unit of Gastroenterology and Endoscopy, Fondazione IRCCS Ca’ Granda, Ospedale Policlinico di Milano, Via Francesco Sforza, 35, 20122 Milano, Italy

# correspondence to:

Yvan Torrente, Dipartimento di Fisiopatologia medico-chirurgica e dei Trapianti, Laboratorio di Cellule Staminali, Università degli Studi di Milano, Fondazione IRCCS Ca’ Granda Ospedale Maggiore Policlinico, Milano, Centro Dino Ferrari, Via Francesco Sforza 35, 20122, Milan, Italy. E-mail: yvan.torrente@unimi.it

**Contents:**

Supplementary Methods

Supplementary Figure + Legends 1 to 7

Supplementary Table 1

Supplementary References

# Animal experiments

Animals were housed at the Policlinico Hospital animal facility in ventilated cages with a 12-hour light/dark cycle and provided with free access to water and standard autoclaved chow. Certified as the Standard Diet by Mucedola (Settimo Milanese, Milano, Italy), the mouse feed is primarily composed of wheat, maize, toasted soybean meal, and corn gluten feed and includes the following nutritional additives per kilogram: Vitamin A (144,000 I.U.), Vitamin D3 (1,260 I.U.), iron (180 mg), and zinc (67.5 mg). Upon arrival, we acclimated the mice to animal facility environment for at least 4 weeks and analysis performed on 3 months-old animals. The immunoproteasome inhibitor ONX-0914 (6 mg/kg, Clini Sciences, France) or vehicle were administered intraperitoneally to 3m mdx mice for five weeks, with two injections per week (n=10). This dosage has been previously shown to ameliorate muscle damage and reduce muscle infiltrating inflammatory cells in mdx mice [[20](#_ENREF_25), [27](#_ENREF_47)] and in a murine model of dysferlinopathy [[21](#_ENREF_26)]. Untreated age-matched mdx and C57Bl mice served as control groups. As it is recognized that food intake in group-housed animals can be significantly influenced by social hierarchy [[S29](#_ENREF_69)], we housed individually the mice to measure the food and the water consumption every week during the treatment as performed in [[S30](#_ENREF_70)] and eliminate possible bias during the evaluation of fecal production and weight. No changes in food or water consumption were observed in either the treated or untreated groups of mice as summarized in the Figure S5.

C57Bl mice treated with ONX-0914 were not used as control since their percentages of innate and adaptive immune cells in the spleen were similar to untreated C57Bl mice [[20](#_ENREF_25)]. To investigate the effects of ONX-0914 on the microbiota, skeletal muscle and eventually other tissues, we depleted the microbiota from a group of 3-month-old mdx mice (n=6) through daily oral gavage with an antibiotic mix (ABX) consisting of Vancomycin (1.25 mg), Ampicillin (2.5 mg), and Metronidazole (1.25 mg) (administered in a 200µl solution for 4 weeks) (mdx+ABX). Subsequently, we treated the animals with ONX-0914 (mdx+ABX+ONX) as specified above. Mice were sacrificed through cervical dislocation according to the Italian country Law. Untreated age-matched mdx and C57Bl mice – as well as 3m mdx+ONX – served as control groups.

For the Fecal Microbiota Transplantation (FMT) experiments, 3 months-old mdx mice (n=4 per group) were pre-treated with the ABX-mix in 200 µl water/one months and then transplanted with feces from mdx (FMT^mdx^) and mdx+ONX (FMT^mdx+ONX^) as described in [[13](#_ENREF_13)]. Two weeks after the FMT, mice were sacrificed and untreated age-matched mdx and C57Bl mice served as control groups.

To perform the FITC-dextran experiment, we fasted the mice (n=6 per group) for 4 hours while maintaining *ad libitum* access to water and we monitored them to limit as possible the coprophagy. 80 mg/μl 4 kDa FITC-dextran (68509, Sigma-Aldrich) was diluted in sterile 1x PBS and 200 mL of solution were injected/mice through oral gavage using an animal feeding needle, size 20G, 1.2 inches x 1.9 mm (L x diam), with ball at the end (CAD9931-100EA, Cadence Science, Japan). Next, we harvested the fecal samples from each mouse 4 hours after the gavage and determined the concentration of FITC-dextran as specified in details in [[S31](#_ENREF_71)].

**Serum analysis**

CPK, ALT, AST analysis was performed on serum samples harvested from 3m C57Bl, mdx, mdx+ONX, and mdx+ABX+ONX mice with CPK/ALT/AST/GLUC3 kit (Cobas), according to manufacturer’s instructions.

**Tetanic force and muscle functional measures**

Tetanic force of TA of 3m C57Bl, mdx, mdx+ONX, mdx+ABX+ONX, FMT^mdx^ and FMT^mdx+ONX^ mice was determined as in [[20](#_ENREF_25)]: we measured the tetanic isometric contractions were studied at L0 (length at which the maximal isometric force is observed) and we normalized the values to muscle cross section area.

**Isolation of murine colonic macrophages**

As already described [[S32](#_ENREF_72), [S33](#_ENREF_73)], colons from treated and untreated mdx mice were carefully washed with cold PBS 1% BSA to eliminate any content trace and then cut longitudinally into 0.5/1cm lengths. The tissues were put on gentle shaker and incubated twice in RPMI 5% FCS, 0.2 mM EDTA for 20 min/37°C, then passed through a 100 mm nylon membrane. To eliminate epithelial and other immune cells, colonic tissues were digested again for 20 min/37°C in RPMI 10% FCS containing collagenase D (0.2 mg/ml) from Sigma (France), dispase-II (3 mg/ml) and Dnase-I (0.2 mg/ml) all from Roche (Germany). Cells were obtained following a centrifugation phase and Percoll gradient (gradient 30%/40%/70%; Sigma).

# Western Blot (WB) analysis

Relative levels of various proteins in skeletal muscle tissue and colonic macrophages of mdx mice were estimated by Western blot as previously described [[13](#_ENREF_13)]. Following the achievement of total proteins, we prepared samples resolved on polyacrylamide gels (ranging from 6% to 15%), transferred to nitrocellulose membranes (Bio-Rad Laboratories, California, USA) and incubated with antibodies overnight.

For skeletal muscle analyses, the following antibodies were used: vinculin (0.3 μg/ml, MA5-11690, Invitrogen); mTOR (80.7 μg/ml, PA-5-34663, Invitrogen); OXPHOS (3 μg/ml, MS604-300, Abcam); AKT1/2/3 (2 μg/ml, ab126811, Abcam); TOMM20 (0.2 μg/ml, AB186735, Abcam); JAK-1 (0.2 μg/ml, sc-136225, Santa Cruz); S6RB (1:600, #2217, Cell Signalling); p70-S6RB (1:600, #9205, Cell Signalling); total 4EBP1 (1:600, #9644, Cell Signalling); phosphorylated 4EBP1 (1:600, #9451, Cell Signalling); PI3K (0.4 μg/ml, sc-1637, Santa Cruz); MAP3K3/MEK kinase-3 (1:500, sc-136260, Santa Cruz); MCP-1 (0.1 μg/ml, sc-52701, Santa Cruz); TNF-α (1.67 μg/ml, AMC3012, Invitrogen); FOXO-1 (1:500, sc-374427, Santa Cruz); IL-6 (1:500, sc-57315, Santa Cruz); TGF-β (1.6 μg/ml, APO635OPU-N, Origene); FH (1.17 μg/ml, E-AB-15051, Elabscience); ACO2 (0.5 μg/ml, E-AB-16130, Elabscience); IDH2 (0.5 μg/ml, E-AB-11319, Elabscience); MDH2 (1.67 μg/ml, E-AB-16130, Elabscience); OGDH (1:600, HPA020347, Sigma); CS (1:600, SAB2701077, Sigma); IL1-β (1.6 μg/ml, Santa Cruz, sc-12742); NF-kB (1.6 μg/ml, 8242S, Cell Signalling).

For colonic macrophages analyses, the following antibodies were used: PSMB8 (2 μg/ml, Proteasome 20S LMP7, ab3329, Abcam); PSMB9 (1.1 μg/ml; Proteasome 20S LMP2 (EPR13785) ab184172, Abcam); TLR2 (0.4 μg/ml, orb229137, Biorbyt): AKT1/2/3 (2 μg/ml, ab126811, Abcam); TNF-α (1.67 μg/ml, AMC3012, Invitrogen); FOXO-1 (1:500, sc-374427, Santa Cruz); IL-6 (1:500, sc-57315, Santa Cruz); TGF-β (1.6 μg/ml, APO635OPU-N, Origene); actin (0.24 μg/ml, ab68167, Abcam).

Filters were detected with peroxidase conjugated secondary antibodies (Agilent Technologies, California, USA) and developed by ECL (Amersham Biosciences, United Kingdom). Bands were quantitated in ImageJ software. The images of uncropped gels are presented in Supplemental File.

**Qualitative (RT-qPCR) experiments**

We extracted total RNA from TA muscle of 3m C57Bl, mdx and mdx+ONX mice, generating the cDNA through the Reverse Transcriptase Kit (ThermoFisher Scientific, California, USA). All the samples that did not rich quality control standards due to the presence of contaminants for RNA were excluded. We quantified the expression of genes through SYBR-Green method, testing all the samples in triplicate. The threshold cycles (Ct) of target genes were normalized against the housekeeping gene – β-actin – and we calculated the relative transcript levels from the Ct values with this formula: X = 2^−ΔΔct^ where X is the fold difference in amount of target gene versus β-actin and ΔCt=Ct_target_−Ct_β-actin_. The efficiency of primers used was calculated between 95.2% and 98.9%.

**Histological and immunofluorescence analysis of tissutal sections.**

For histological and immunofluorescence analysis, we collected TA and colon from 3m C57Bl, mdx, mdx+ONX, mdx+ABX and mdx+ABX+ONX mice, we froze them in liquid-nitrogen cooled isopentane and finally cut on a cryostat into 10µm slices. All the samples that did not rich quality control standards due to problems in freezing procedures for histological analysis were excluded. H&E staining were performed as in [[S34](#_ENREF_74)] to evaluate the morphology of muscles. SDH enzymatic activity was assessed by placing the slides in SDH incubating solution, containing sodium succinate as a substrate and nitro-blue tetrazolium (NBT) for visualization of reaction for 60 minutes/37°C. Slides were incubated for 10 seconds in 30-60-90-60-30% acetone solution and then 30 seconds in 80-90-100% ethanol solution for dehydration. As a final step, slides were incubated for 60 seconds with 100% Xylene (Sigma-Aldrich, USA) and mounted with DPX reagent (VWR International, USA) and coverslips.

For Picro Sirius Red staining, frozen sections were warmed at RT and fixed in Bouin’s solution (#7000.1000, VWR International, USA) in a water bath at 56°C for 15 minutes. Sections were then washed three times for 5 minutes/wash in distilled water at room temperature and incubated in Picro Sirius Red solution (#ab150681, Abcam, UK) for 1 h at room temperature. Following two washes in acidified water (#ab150681, Abcam, UK) for 30 seconds each, the sections were dehydrated in 70%, 95% and 100% ethanol (30 seconds each) and mounted with DPX reagent (VWR International, USA) and coverslips.

For immunofluorescence staining, the sections were incubated overnight at 4°C with the following primary antibodies: Mannose Receptor (CD206) (10 μg/ml, ab64693, Abcam); CD68-conjugated Alexa Fluor 647 (50 μg/ml, ab201845, Abcam); CD3 (0.3 μg/ml, AB135372, Abcam, UK); CD31 (10 μg/ml, ab119339, Abcam);

Isolectin GS-IB4 Alexa Fluor™ 594 Conjugate (10 μg/ml, I21413, Thermo Fisher Scientific); α-Smooth Muscle – FITC (20 μg/ml, F3777, Sigma-Aldrich); fibers type I (0.5 μg/ml, BA-D5), fibers type IIA (0.5 μg/ml, sc-71) and fibers type IIB (0.5 μg/ml, BF-F3), all from Developmental Studies Hybridoma Bank (Douglas Houston). For JAM-A (4 μg/ml, sc-53623, Santa Cruz) immunofluorescence, we used 10% goat serum. Fluorochrome-conjugated secondary antibodies (1:200, diluted in PBS1X) were added for 1h at room temperature. Slides were then mounted with Prolong Gold® Antifade Reagent with DAPI (Thermo Fisher, Carlsbad, CA). A GA3 analysis was conducted to quantify JAM-A using Nis-element software, employing preprocessing filters for each channel (blue-green). In the blue channel, correction filters such as automatic shadow correction and sharpening were applied to correct the background signal and better define the specific signal. Segmentation was performed using the bright spot function to identify specific signal spots, which were then enlarged to obtain an optimal calculation of the total area of interest. In the green channel, the same pre-processing filters used in the previous channel were applied, while segmentation utilized a specific range of thresholds, along with an area filter to exclude any nonspecific spots or those outside the region of interest. Only spots appearing in both the green and blue channel masks were measured. At the end of the analysis, the ratio of the area of the green mask to the total area represented by the blue mask was calculated.

**Metabolome analysis**

*Metabolomics Profiling*

The MetaboPrep GC kit, manufactured by Theoreo srl (Montecorvino Pugliano, SA, Italy), was employed for the extraction, purification, and derivatization of serum samples in accordance with the methodology outlined by Troisi et al. [[S35](#_ENREF_75), [S36](#_ENREF_76)]. This particular approach yields untargeted metabolomic profiles. Briefly, 50 μL of serum or 50 mg of tissue sample was combined with an extraction solution based on alcohol and 2-isopropyl malic acid, which served as an internal standard. This mixture was then subjected to vortexing for a duration of 30 minutes at 1250 rpm, followed by centrifugation at 1600 rpm for 5 minutes at a temperature of 4°C. Subsequently, the resulting supernatant (200 μL) was transferred to fresh Eppendorf tubes, treated with a purification solution, and vortexed for 30 seconds at 1250 rpm. Another round of centrifugation at 1600 rpm for 5 minutes at 4°C was then performed. The resulting supernatants (175 μL) were transferred to 2 mL glass vials and subjected to overnight freeze-drying. The process of derivatization took place in two phases: firstly, 50 μL of a methoxylamine hydrochloride solution in pyridine was added and vortexed for 90 minutes at 1200 rpm. Subsequently, 25 μL of a derivatizing solution containing N,O-Bis(trimethylsilyl)trifluoroacetamide (BSTFA) was added, and the vials were vortexed again for 90 minutes at 1200 rpm. Finally, the resulting 75 μL of derivatized metabolome was placed in a GC vial equipped with a low-volume insert for auto-sampler injection. Prior to injection into the GC-MS system, the vials were centrifuged at 16,000 rpm for 5 minutes at a temperature of 4°C. The analysis phase was performed using the GCMS-2010SE instrument, manufactured by Shimadzu Corp. in Kyoto, Japan. Chromatographic separation was achieved utilizing a 30 mm × 0.25 mm CP-Sil 8 CB fused silica capillary column with a film thickness of 1.00 μm, procured from Agilent (Agilent, J&W, Santa Clara, CA, USA), with helium serving as the carrier gas. Initially, the oven temperature was set to 100°C and maintained for 1 minute, followed by a gradual increase to 320°C at a rate of 6°C per minute, with an additional hold time of 2.33 minutes. The gas flow was adjusted to ensure a constant linear speed of 39 cm/s, while the split flow was set to 1:5. The mass spectrometer was operated utilizing electron impact ionization (70 eV) in full scan mode, covering a range of 35-600 m/z, with a scan velocity of 3333 amu/sec and a solvent delay of 5 minutes. Peak identification was accomplished by setting the maximum tolerance for Kovats index difference to 50, while the minimum matching for the NIST library search was set to 85%.

The research involved partitioning the specimens into groups of 25 and subjecting each group to four distinct controls. The controls utilized in the study consisted of an injection of instrument blank, an injection of a standard mix, an injection of a pooled sample solution, and a duplicated injection of a randomly selected sample from the batch. For the instrument blank, 2 μL of hexane were utilized, while the standard mixture consisted of 15 molecules that had undergone the same derivatization process as the samples, encompassing organic acids, sugars, amino acids, steroids, and fatty acids. The pooled sample comprised 2 μL of 50 randomly selected derivatized samples, and the duplicate injection involved a random sample extracted from the batch.

For a batch to be considered validated, four criteria had to be met: the solvent blank should not yield any peaks, the peak areas of all analytes in the standard mix (normalized to the internal standard peak area) must remain within 10% of the expected value, the variation among the peak areas (normalized to the internal standard) of the 100 highest peaks from the repeated injection should be less than 15% of the first injection, and the pooled sample must fall within the same area as the other pooled samples, specifically accounting for less than 5% of the total area in a model constructed using all the samples analyzed.

Only gas chromatography–mass spectrometry signals that were consistently present in at least 80% of the samples were taken into consideration. Peaks corresponding to metabolites at low concentrations, which consequently exhibited poor mass spectral quality, were not subjected to further examination. Additionally, signals stemming from the same metabolites, such as sugars resulting in multiple derivatization products, were treated as independent characteristics.

*Data Analysis*

In the realm of metabolomics data analysis, the initial step involved organizing the raw data derived from chromatographic investigations into a tabular structure, where each sample corresponded to a row and each variable or metabolite was represented by a column. Prior to the analysis phase, logarithmic transformation was applied to the raw chromatographic signal intensities Then the peak areas of each metabolite was normalized with respect to an internal standard area. Subsequently, these transformed values underwent a scaling process known as autoscaling, which centered the values around their mean and divided them by the standard deviation specific to each variable. Samples were divided into training and test sets, maintaining a ratio of 66:34. The training set was utilized to train a PLS-DA model using the MetaboPredict software (Theoreo srl, Montecorvino Pugliano, SA, Italy).

During the model training process, cross-validation was employed to assess model accuracy, and a permutation test was applied in order to prevent overfitting.

*Pathway analysis*

The investigation into metabolic pathways was carried out by employing a combination of pathway enrichment analysis and pathway topology analysis, following the prescribed methodology introduced by Xia and Wishart [[S37](#_ENREF_77)]. The analysis relied on the KEGG metabolic pathways as the foundational knowledge base and was conducted using the web application of the MetPa algorithm. The initial phase encompassed an over-representation analysis, wherein the hypergeometric test was employed to discern whether specific compounds exhibited a disproportionate presence within a given pathway compared to random occurrences. The subsequent step involved pathway topology analysis, which incorporated structural insights into the pathways. Here, betweenness centrality served as a measure of node centrality to gauge the significance of each node. To address the consideration of multiple pathways, the statistical p-values obtained from the enrichment analysis underwent adjustment for multiple testing employing the false discovery rate (FDR) method. Subsequently, the Impact, which denotes the pathway's impact value, was computed based on the outcomes derived from the pathway topology analysis.

**Amplicon microbiota analysis**

We performed DNA extraction, 16S rRNA gene amplification, purification, library preparation and pair-end sequencing from 3m mdx, mdx+vehicle and mdx+ONX on the Illumina MiSeq platform as previously described in [[S38](#_ENREF_78)]. MICCA pipeline (v.1.7.0) (<https://micca.readthedocs.io/en/latest/index.html>) was used to pre-process the reads [[S39](#_ENREF_79)] while micca trim and micca filter were employed for forward/reverse primers trimming and quality filtering, respectively. Filtered sequences were denoised using the UNOISE algorithm implemented in micca otu to determine true biological sequences at the single nucleotide resolution by generating amplicon sequence variants (ASVs). Micca classify and the Ribosomal Database Project (RDP) Classifier v2.11 were used to classify taxonomically the bacterial ASVs [[S40](#_ENREF_80)] while Nearest Alignment Space Termination (NAST) algorithm performed the multiple sequence alignment (MSA) of 16S sequences [[S41](#_ENREF_81)] as described in detail in [[S42](#_ENREF_82)] (release 13_08). Phylogenetic trees were inferred using micca tree [[S43](#_ENREF_83)]. Indeed, we reduced sampling heterogeneity rarefying samples at the depth of the less abundant sample using micca tablerare while we performed the phyloseq R package to assess alpha (within-sample richness) and beta- diversity (between-sample dissimilarity) [[S44](#_ENREF_84)]. P-values were False Discovery Rate corrected using the Benjamini-Hochberg procedure implemented in DESeq2 and the R package DESeq2 was used to test differential abundance [[S45](#_ENREF_85)]. The psych R package was used to compute Spearman’s correlation tests. Prediction of functional metagenomic content was inferred by using Piphillin [[S46](#_ENREF_86)] with the reference curated databases BioCyc [[S47](#_ENREF_87)] and Kyoto Encyclopedia of Genes and Genomes (KEGG) [[S48](#_ENREF_88)]. iPATH 3 was used to construct the metabolic pathway (<https://pathways.embl.de/>).

**Shotgun microbiota analysis**

*Library preparation and sequencing*

The sequencing libraries were prepared with the Illumina DNA Prep, (M) Tagmentation (96 Samples, IPB) kit (Illumina, #20060059) in combination with the UD for Illumina DNA/RNA UD Indexes Set B (Illumina, 20091647) and the amplified libraries were purified with the double-sided bead purification procedure, as described by the Illumina protocol. Then, libraries concentration (ng/µl) were quantified with the Quant-iT™ 1X dsDNA Assay Kits, HS (Life Technologies, #Q33232) in combination with the Varioskan LUX Microplate Reader (Thermo Fisher Scientific, #VL0000D0). In addition, the base pair length (bp) was evaluated by using the D5000 ScreenTape Assay (Agilent, #5067-5588/9) in combination with the TapeStation 4150 (Agilent Technologies, #G2992AA). By knowing both library concentration and base pair length, it is possible to obtain the correct library volume to pool in the same tube to achieve optimal cluster density. The library pool was then quantified with the Qubit 1x dsDNA HS kit (Life Technologies, #Q33231) through the Qubit® 3.0 Fluorometer (Life Technologies, #Q33216) and the base pair length (bp) was evaluated as described before. Finally, the library pools were sequenced using the Novaseq X Plus platform (Illumina) at an average depth of 7,5 Gb per sample.

*Preprocessing and quality control*

Preprocessing and quality control of the sequenced samples was performed using an standalone pipeline available at <https://github.com/SegataLab/preprocessing>. Briefly, the software TrimGalore (version 0.6.6; https://github.com/FelixKrueger/TrimGalore) was used for the read-level quality control step: reads with a quality score < 20, fragmented short reads (length < 75) and reads with more than 5 ambiguous nucleotides were removed. During the following screening for contaminant DNA, mouse DNA (NCBI entry: GCA_000001635) was removed using Kraken2 (version 2.0.7) [2[2](#_ENREF_27)].

*Taxonomic and functional profiling*

Taxonomic profiling was performed using MetaPhlAn (version 4.1.1) [[S49](#_ENREF_89)] against the Jun23 database with default parameters. Community-level functional profiling was performed using HUMAnN (version 4.0.alpha.1) [[S50](#_ENREF_90)] at gene family (based on UniRef90 gene families) and at pathway levels (using the MetaCyc database [[S51](#_ENREF_91)]).

**Measurement of lipocalin-2**

Approximately 50 mg of frozen feces were diluted 10-fold in 0.1% Tween-20/D-PBS, vortexed for 20 minutes, and centrifuged at 12,000 × g for 20 minutes at 4 °C. The supernatant was collected and subjected to a second centrifugation (12,000 × g for 20 minutes at 4 °C). The resulting upper aqueous portion was used to measure lipocalin-2 concentration using the Mouse Lipocalin-2/NGAL Quantikine ELISA Kit® (R&D Systems), following the manufacturer's protocol.

# Statistics

To allocate the animals to different experimental procedures, we used the randomization within blocks. To avoid that the efficacy of ONX-0914 treatment on mdx mice was not correctly interpreted worsening the reliability of our results, animal handlers were blinded regarding the treatment that the mice received throughout all the experimental procedures. Animals that suffered from clinical complications as enhancement of stress or motor impairments were excluded from the experimental plan and eventually sacrificed. Sample-size calculator freely available on internet was used to assess sample size. Alpha and beta diversity analyses were performed on the taxonomic and functional profiles using the SciKit-bio (version 0.5.6), SciKit-learn (version 1.2.2) and SciPy (version 1.10.1) python libraries.

**Supplementary Figures Legend**

**FigS1. Evaluation of colon morphology in 3m mdx+ONX-0914 mice**

**(A**) Representative images of H&E staining of colon from 3m C57Bl, mdx and mdx+ONX mice. High magnification (scale bar: 200 μm) and low magnification (scale bar: 500 μm). (**B**) JAM-A expression was evaluated in colonic tissues of 3m C57Bl, mdx, mdx+ABX, mdx+ONX and mdx+ABX+ONX mice (n=5 each, with 5 images analyzed per animal). JAM-A staining is shown in green and DAPI in blue. CD68+ M1 and CD206+ M2 macrophages were quantified in colonic tissues of 3m C57Bl, mdx, mdx+ABX, mdx+ONX and mdx+ABX+ONX mice (n=5 each, with 5 images analyzed per animal). CD206 staining is shown in green, CD68 in red and DAPI in blue. Scale bars: 100 μm. Cropped images of representative WBs show the expression of pro-inflammatory proteins in macrophages isolated from colon tissues of 3m C57Bl, mdx, and mdx+ONX mice (n=4, two independent experiments)**(C)** and from 3m C57Bl, mdx, mdx+ABX, mdx+ONX, and mdx+ABX+ONX mice (n=3 each, two independent experiments) **(D).** Densitometric analyses of protein expression are shown as a ratio to actin. Data information: data are presented as mean ± SD (*p<0.05; **p<0.01, ***p<0.001, ****p<0.0001; One-Way ANOVA Kruskal-Wallis test for evaluation of images and One-Way ANOVA with Tukey's multiple comparisons test for WB experiments).

**FigS2. Microbiota richness is similar between mdx and vehicle-treated mdx mice**

(**A**) Analysis of alpha-diversity (Wilcoxon sum rank test, p=0.56) as measured by using the total number of observed amplicon sequence variants (ASV) in mdx (CTRL) and vehicle-treated mdx (VEH) mice (n=4 each). (**B-D**) Analysis of beta-diversity as measured by using the (**B**) unweighted, (**C**) weighted UniFrac distances and (**D**) Bray-Curtis dissimilarity index (PERMANOVA, p>0.05). (**E**) Stacked barplots representing the relative abundance of the 25 most abundant taxa classified to the genus level per each sample

**FigS3. Gut tissue metabolome profiling in 3-month-old C57Bl, mdx, and mdx+ONX mice**.

(**A**) Heatmap showing all the relevant metabolites concentration change among 3m C57Bl (n=4), mdx (n=4) and mdx+ONX (n=3) with a p-value <0.05 according to ANOVA. Both metabolites and classes were clusterized according to the Wald method. Bacterial metabolites: 4-hydroxybutanoic acid, butanoic acid, 5-keto gluconate and octadecanamide. Metabolites involved in fatty acid synthesis and degradation: dodecanoic acid, palmitic acid, 5-hydroxyhexanoic acid. Metabolites involved in simple sugar metabolism: glyceraldehyde-3-phospahte, fucose, erythrose, erythrose-4-phosphate, ribose, arabinose, xylitol, ribitol, mannose, fructose, glucose-6-phosphate, glucose, rhamnose, galactitol, mannitol, melibiose, maltose, lactic acid, glycolic acid, oxalic acid. (**B**) Metabolic pathways involving the relevant metabolites obtained using the MetPa algorithm. The colour and size of each circle are based on the p-value and pathway impact value, respectively. The x-axis represents the pathway impact, and the y-axis represents the −log of p values from the pathway enrichment analysis for the key differential metabolites of 3m C57Bl, mdx and mdx+ONX.

**FigS4. Mitochondrial enzymes activity in muscles of 3m mdx+ONX-0914 mice**

Enzymatic activity of mitochondrial enzymes involved in respiratory chain complexes of TA and DIA of 3m C57Bl, mdx and mdx+ONX mice (n=3 each). The following abbreviations were used in the picture (NADH DH/citr synt: NADH dehydrogenase/citrate synthase; NADH ubiq 1 red/cit synt: NADH ubiquinone 1 reductase/citrate synthase; succinate DH/citr synt: succinate dehydrogenase/citrate synthase; succinate CoQ red/citr synt: succinate CoQ reductase/citrate synthase; cytr ox/citr synt: cytochrome oxidase/citrate synthase; NADH cit C red/citr synt: NADH citrate C reductase/citrate synthase; and succinate cit C red/citr synt: succinate citrate C reductase/citrate synthase).

Data information: data are presented as mean ± SD (*p<0.05; **p<0.01, ***p<0.001; One-Way ANOVA with Tukey's multiple comparisons test).

**FigS5. Proteomic evaluation of microbiota-depleted ONX-treated skeletal muscles**

Cropped images of representative WB analysis of TA muscle of (**A**) 3m C57Bl and mdx (n=3 each), mdx^FTMmdx^ and mdx^FTMmdx+ONX^ (n=4 each) mice and of (**B**) 3m mdx, mdx+ABX, mdx+ONX and mdx+ABX+ONX mice (n=3 each, two independent experiments) showing the expression of the proteins specifically involved in mitochondrial functions and TCA complex; OXPHOS complex (C1: NDUFB8; C2: SDHB; C3: UQCRC2; C4: MTCO1; C5: ATP5A); mTOR-dependent pathways; M1- and M2-macrophages proliferation, skeletal muscle metabolism and pro-inflammatory cytokines.

OGDH: Oxoglutarate Dehydrogenase; CS: Citrate Synthase; MDH2: Malate Dehydrogenase 2; FH: Fumarate Hydratase; ACO2: Aconitase 2; IDH2: Isocitrate Dehydrogenase NADP(+) 2.

Data information: densitometric data were normalized on vinculin and expressed as mean±SD (*p<0.05, **p<0.01, ***p<0.001; ****p<0.0001, ordinary one-way ANOVA, Tuckey multiple comparison test).

**FigS6. Representative staining of muscles following ONX-0914 treatment and gut microbiota modulation**

Representative staining of muscle sections expressing different MyHC isoforms (Type IIa in green, IIx in black, IIb in red; Type I in blu) in TAs of C57Bl, mdx, mdx+ABX, mdx+ONX and mdx+ABX+ONX mice and mdx^FTMmdx^ and mdx^FTMmdx+ONX^ mice (n=3 each and n=10 images per animal) (two independent experiments). Scale bar: 100 μm. Representative SDH staining of TAs from C57Bl, mdx, mdx+ABX, mdx+ONX and mdx+ABX+ONX mice (n=5 each and n=8 images per animal) and mdx^FTMmdx^ and mdx^FTMmdx+ONX^ mice (n=4 each and n=10 images per animal) (two independent experiments); EE staining of C57Bl, mdx, mdx+ABX, mdx+ONX and mdx+ABX+ONX mice and mdx^FTMmdx^ and mdx^FTMmdx+ONX^ mice (n=4 each); Syrius Red staining of TAs from C57Bl, mdx, mdx+ABX, mdx+ONX and mdx+ABX+ONX mice (n=3 each and n=10 images per animal) and mdx^FTMmdx^ and mdx^FTMmdx+ONX^ mice (n=3 each and n=10 images per animal) (two independent experiments). Scale bar: 200 μm.

**FigS7. Food and water consumption of ONX-0914-treated mdx mice.**

Food and water consumption calculated on the weight of each animal in 3m C57Bl (n=6), mdx (n=7) and mdx+ONX (n=8)

**Supplementary Table 1**

List of primers used in RT-qPCR experiments

| ***ccl2*** |
| --- |
| F- CATCATGCCTACCCTGCGTGTCCC |
| R- CCTCCTCCCTAGGACAGTTTATGGA |
| ***il-10*** |
| F- TAATAAGCTCCAAGACCAAGG |
| R- CATCATGTATGCTTCTATGCAG |
| ***icam-1*** |
| F- GCATCCTGACCAGTAGAGAAAC |
| R- AGTACATCAGTGAGGAGGTGAA |
| ***vicam-1*** |
| F- GGTCACTGGGTTGACTTTCA |
| R- TCAGGTTAGTCATTACACAAGACC |

**Supplementary References**

S1. Coeffier M, Gloro R, Boukhettala N, Aziz M, Lecleire S, Vandaele N, et al. Increased proteasome-mediated degradation of occludin in irritable bowel syndrome. The American journal of gastroenterology. 2010;105:1181-8. doi:10.1038/ajg.2009.700

S2. Visekruna A, Joeris T, Seidel D, Kroesen A, Loddenkemper C, Zeitz M, et al. Proteasome-mediated degradation of IkappaBalpha and processing of p105 in Crohn disease and ulcerative colitis. The Journal of clinical investigation. 2006;116:3195-203. doi:10.1172/JCI28804

S3. Basler M, Dajee M, Moll C, Groettrup M, Kirk CJ. Prevention of experimental colitis by a selective inhibitor of the immunoproteasome. Journal of immunology. 2010;185:634-41. doi:10.4049/jimmunol.0903182

S4. Koerner J, Brunner T, Groettrup M. Inhibition and deficiency of the immunoproteasome subunit LMP7 suppress the development and progression of colorectal carcinoma in mice. Oncotarget. 2017;8:50873-88. doi:10.18632/oncotarget.15141

S5. Rouette A, Trofimov A, Haberl D, Boucher G, Lavallee VP, D'Angelo G, et al. Expression of immunoproteasome genes is regulated by cell-intrinsic and -extrinsic factors in human cancers. Scientific reports. 2016;6:34019. doi:10.1038/srep34019

S6. Schneider CA, Rasband WS, Eliceiri KW. NIH Image to ImageJ: 25 years of image analysis. Nature methods. 2012;9:671-5. doi:10.1038/nmeth.2089

S7. Segata N, Izard J, Waldron L, Gevers D, Miropolsky L, Garrett WS, et al. Metagenomic biomarker discovery and explanation. Genome biology. 2011;12:R60. doi:10.1186/gb-2011-12-6-r60

S8. Vetrano S, Danese S. The role of JAM-A in inflammatory bowel disease: unrevealing the ties that bind. Annals of the New York Academy of Sciences. 2009;1165:308-13. doi:10.1111/j.1749-6632.2009.04045.x

S9. Laukoetter MG, Nava P, Lee WY, Severson EA, Capaldo CT, Babbin BA, et al. JAM-A regulates permeability and inflammation in the intestine in vivo. The Journal of experimental medicine. 2007;204:3067-76. doi:10.1084/jem.20071416

S10. Sandri M, El Meslemani AH, Sandri C, Schjerling P, Vissing K, Andersen JL, et al. Caspase 3 expression correlates with skeletal muscle apoptosis in Duchenne and facioscapulo human muscular dystrophy. A potential target for pharmacological treatment? Journal of neuropathology and experimental neurology. 2001;60:302-12. doi:10.1093/jnen/60.3.302

S11. Solier S, Mondini M, Meziani L, Jacquel A, Lacout C, Berghe TV, et al. Caspase Inhibition Modulates Monocyte-Derived Macrophage Polarization in Damaged Tissues. International journal of molecular sciences. 2023;24:doi:10.3390/ijms24044151

S12. Rong SJ, Yang CL, Wang FX, Sun F, Luo JH, Yue TT, et al. The Essential Role of FoxO1 in the Regulation of Macrophage Function. BioMed research international. 2022;2022:1068962. doi:10.1155/2022/1068962

S13. Leigh SJ, Uhlig F, Wilmes L, Sanchez-Diaz P, Gheorghe CE, Goodson MS, et al. The impact of acute and chronic stress on gastrointestinal physiology and function: a microbiota-gut-brain axis perspective. The Journal of physiology. 2023;601:4491-538. doi:10.1113/JP281951

S14. Rothschild D, Weissbrod O, Barkan E, Kurilshikov A, Korem T, Zeevi D, et al. Environment dominates over host genetics in shaping human gut microbiota. Nature. 2018;555:210-5. doi:10.1038/nature25973

S15. Wu G, Fang YZ, Yang S, Lupton JR, Turner ND. Glutathione metabolism and its implications for health. J Nutr. 2004;134:489-92. doi:10.1093/jn/134.3.489

S16. Conte F, van Buuringen N, Voermans NC, Lefeber DJ. Galactose in human metabolism, glycosylation and congenital metabolic diseases: Time for a closer look. Biochimica et biophysica acta General subjects. 2021;1865:129898. doi:10.1016/j.bbagen.2021.129898

S17. Harding JW, Jr., Pyeritz EA, Copeland ES, White HB, 3rd. Role of glycerol 3-phosphate dehydrogenase in glyceride metabolism. Effect of diet on enzyme activities in chicken liver. Biochem J. 1975;146:223-9. doi:10.1042/bj1460223

S18. Sohal RS, Weindruch R. Oxidative stress, caloric restriction, and aging. Science. 1996;273:59-63. doi:10.1126/science.273.5271.59

S19. Kodron A, Mussulini BH, Pilecka I, Chacinska A. The ubiquitin-proteasome system and its crosstalk with mitochondria as therapeutic targets in medicine. Pharmacological research. 2021;163:105248. doi:10.1016/j.phrs.2020.105248

S20. Meul T, Berschneider K, Schmitt S, Mayr CH, Mattner LF, Schiller HB, et al. Mitochondrial Regulation of the 26S Proteasome. Cell reports. 2020;32:108059. doi:10.1016/j.celrep.2020.108059

S21. Ghouzali I, Lemaitre C, Bahlouli W, Azhar S, Bole-Feysot C, Meleine M, et al. Targeting immunoproteasome and glutamine supplementation prevent intestinal hyperpermeability. Biochim Biophys Acta. 2017;1861:3278-88. doi:10.1016/j.bbagen.2016.08.010

S22. Scott NA, Lawson MAE, Hodgetts RJ, Le Gall G, Hall LJ, Mann ER. Macrophage metabolism in the intestine is compartment specific and regulated by the microbiota. Immunology. 2022;166:138-52. doi:10.1111/imm.13461

S23. den Besten G, Bleeker A, Gerding A, van Eunen K, Havinga R, van Dijk TH, et al. Short-Chain Fatty Acids Protect Against High-Fat Diet-Induced Obesity via a PPARgamma-Dependent Switch From Lipogenesis to Fat Oxidation. Diabetes. 2015;64:2398-408. doi:10.2337/db14-1213

S24. Albornoz N, Álvarez-Indo J, de la Peña A, Munoz EA, Coca A, et al*.* Targeting the immunoproteasome in hypothalamic neurons as a novel therapeutic strategy for high-fat diet-induced obesity and metabolic dysregulation. J Neuroinflammation. 2024;21:191. doi:10.1186/s12974-024-03154-z

S25. Devaraj S, Jialal I. C-reactive protein polarizes human macrophages to an M1 phenotype and inhibits transformation to the M2 phenotype. Arteriosclerosis, thrombosis, and vascular biology. 2011;31:1397-402. doi:10.1161/ATVBAHA.111.225508

S26. Kaplan JL, Marshall MA, C CM, Harmon DB, Garmey JC, Oldham SN, et al. Adipocyte progenitor cells initiate monocyte chemoattractant protein-1-mediated macrophage accumulation in visceral adipose tissue. Molecular metabolism. 2015;4:779-94. doi:10.1016/j.molmet.2015.07.010

S27. He Y, Wang L, Liu W, Zhong J, Bai S, Wang Z, et al. MAP3K3 expression in tumor cells and tumor-infiltrating lymphocytes is correlated with favorable patient survival in lung cancer. Scientific reports. 2015;5:11471. doi:10.1038/srep11471

S28. Dalal PJ, Sumagin R. Emerging Functions of ICAM-1 in Macrophage Efferocytosis and Wound Healing. Journal of cellular immunology. 2020;2:250-3. doi:10.33696/immunology.2.051

S29. Tamashiro KL, Hegeman MA, Nguyen MM, Melhorn SJ, Ma LY, Woods SC, et al. Dynamic body weight and body composition changes in response to subordination stress. Physiology & behavior. 2007;91:440-8. doi:10.1016/j.physbeh.2007.04.004

S30. Mei J, Yu S, Ahren B. Study on administration of 1,5-anhydro-D-fructose in C57BL/6J mice challenged with high-fat diet. BMC endocrine disorders. 2010;10:17. doi:10.1186/1472-6823-10-17

S31. Gerkins C, Hajjar R, Oliero M, Santos MM. Assessment of Gut Barrier Integrity in Mice Using Fluorescein-Isothiocyanate-Labeled Dextran. Journal of visualized experiments : JoVE. 2022;doi:10.3791/64710

S32. Rahabi M, Jacquemin G, Prat M, Meunier E, AlaEddine M, Bertrand B, et al. Divergent Roles for Macrophage C-type Lectin Receptors, Dectin-1 and Mannose Receptors, in the Intestinal Inflammatory Response. Cell reports. 2020;30:4386-98 e5. doi:10.1016/j.celrep.2020.03.018

S33. Weigmann B, Tubbe I, Seidel D, Nicolaev A, Becker C, Neurath MF. Isolation and subsequent analysis of murine lamina propria mononuclear cells from colonic tissue. Nature protocols. 2007;2:2307-11. doi:10.1038/nprot.2007.315

S34. Farini A, Sitzia C, Villa C, Cassani B, Tripodi L, Legato M, et al. Defective dystrophic thymus determines degenerative changes in skeletal muscle. Nature communications. 2021;12:2099. doi:10.1038/s41467-021-22305-x

S35. Troisi J, Mollo A, Lombardi M, Scala G, Richards SM, Symes SJK, et al. The Metabolomic Approach for the Screening of Endometrial Cancer: Validation from a Large Cohort of Women Scheduled for Gynecological Surgery. Biomolecules. 2022;12:doi:10.3390/biom12091229

S36. Troisi J, Tafuro M, Lombardi M, Scala G, Richards SM, Symes SJK, et al. A Metabolomics-Based Screening Proposal for Colorectal Cancer. Metabolites. 2022;12:doi:10.3390/metabo12020110

S37. Xia J, Wishart DS. Using MetaboAnalyst 3.0 for Comprehensive Metabolomics Data Analysis. Current protocols in bioinformatics. 2016;55:14 0 1- 0 91. doi:10.1002/cpbi.11

S38. Burrello C, Giuffre MR, Macandog AD, Diaz-Basabe A, Cribiu FM, Lopez G, et al. Fecal Microbiota Transplantation Controls Murine Chronic Intestinal Inflammation by Modulating Immune Cell Functions and Gut Microbiota Composition. Cells. 2019;8:doi:10.3390/cells8060517

S39. Albanese D, Fontana P, De Filippo C, Cavalieri D, Donati C. MICCA: a complete and accurate software for taxonomic profiling of metagenomic data. Scientific reports. 2015;5:9743. doi:10.1038/srep09743

S40. Wang Q, Garrity GM, Tiedje JM, Cole JR. Naive Bayesian classifier for rapid assignment of rRNA sequences into the new bacterial taxonomy. Applied and environmental microbiology. 2007;73:5261-7. doi:10.1128/AEM.00062-07

S41. DeSantis TZ, Jr., Hugenholtz P, Keller K, Brodie EL, Larsen N, Piceno YM, et al. NAST: a multiple sequence alignment server for comparative analysis of 16S rRNA genes. Nucleic acids research. 2006;34:W394-9. doi:10.1093/nar/gkl244

S42. DeSantis TZ, Hugenholtz P, Larsen N, Rojas M, Brodie EL, Keller K, et al. Greengenes, a chimera-checked 16S rRNA gene database and workbench compatible with ARB. Applied and environmental microbiology. 2006;72:5069-72. doi:10.1128/AEM.03006-05

S43. Price MN, Dehal PS, Arkin AP. FastTree 2--approximately maximum-likelihood trees for large alignments. PLoS One. 2010;5:e9490. doi:10.1371/journal.pone.0009490

S44. McMurdie PJ, Holmes S. phyloseq: an R package for reproducible interactive analysis and graphics of microbiome census data. PLoS One. 2013;8:e61217. doi:10.1371/journal.pone.0061217

S45. Love MI, Huber W, Anders S. Moderated estimation of fold change and dispersion for RNA-seq data with DESeq2. Genome biology. 2014;15:550. doi:10.1186/s13059-014-0550-8

S46. Narayan NR, Weinmaier T, Laserna-Mendieta EJ, Claesson MJ, Shanahan F, Dabbagh K, et al. Piphillin predicts metagenomic composition and dynamics from DADA2-corrected 16S rDNA sequences. BMC genomics. 2020;21:56. doi:10.1186/s12864-019-6427-1

S47. Caspi R, Altman T, Billington R, Dreher K, Foerster H, Fulcher CA, et al. The MetaCyc database of metabolic pathways and enzymes and the BioCyc collection of Pathway/Genome Databases. Nucleic acids research. 2014;42:D459-71. doi:10.1093/nar/gkt1103

S48. Kanehisa M, Goto S. KEGG: kyoto encyclopedia of genes and genomes. Nucleic acids research. 2000;28:27-30. doi:10.1093/nar/28.1.27

S49. Blanco-Miguez A, Beghini F, Cumbo F, McIver LJ, Thompson KN, Zolfo M, et al. Extending and improving metagenomic taxonomic profiling with uncharacterized species using MetaPhlAn 4. Nature biotechnology. 2023;41:1633-44. doi:10.1038/s41587-023-01688-w

S50. Beghini F, McIver LJ, Blanco-Miguez A, Dubois L, Asnicar F, Maharjan S, et al. Integrating taxonomic, functional, and strain-level profiling of diverse microbial communities with bioBakery 3. eLife. 2021;10:doi:10.7554/eLife.65088

S51. Karp PD, Riley M, Paley SM, Pellegrini-Toole A. The MetaCyc Database. Nucleic acids research. 2002;30:59-61. doi:10.1093/nar/30.1.59
