# Supplementary material for: Immunoproteasome Inhibition Positively Impacts the Gut‐Muscle Axis in Duchenne Muscular Dystrophy
Source: J Cachexia Sarcopenia Muscle. 2025 Oct 1;16(5):e70054. doi: 10.1002/jcsm.70054 (PMC12489020; doi:10.1002/jcsm.70054)

Figure S1C

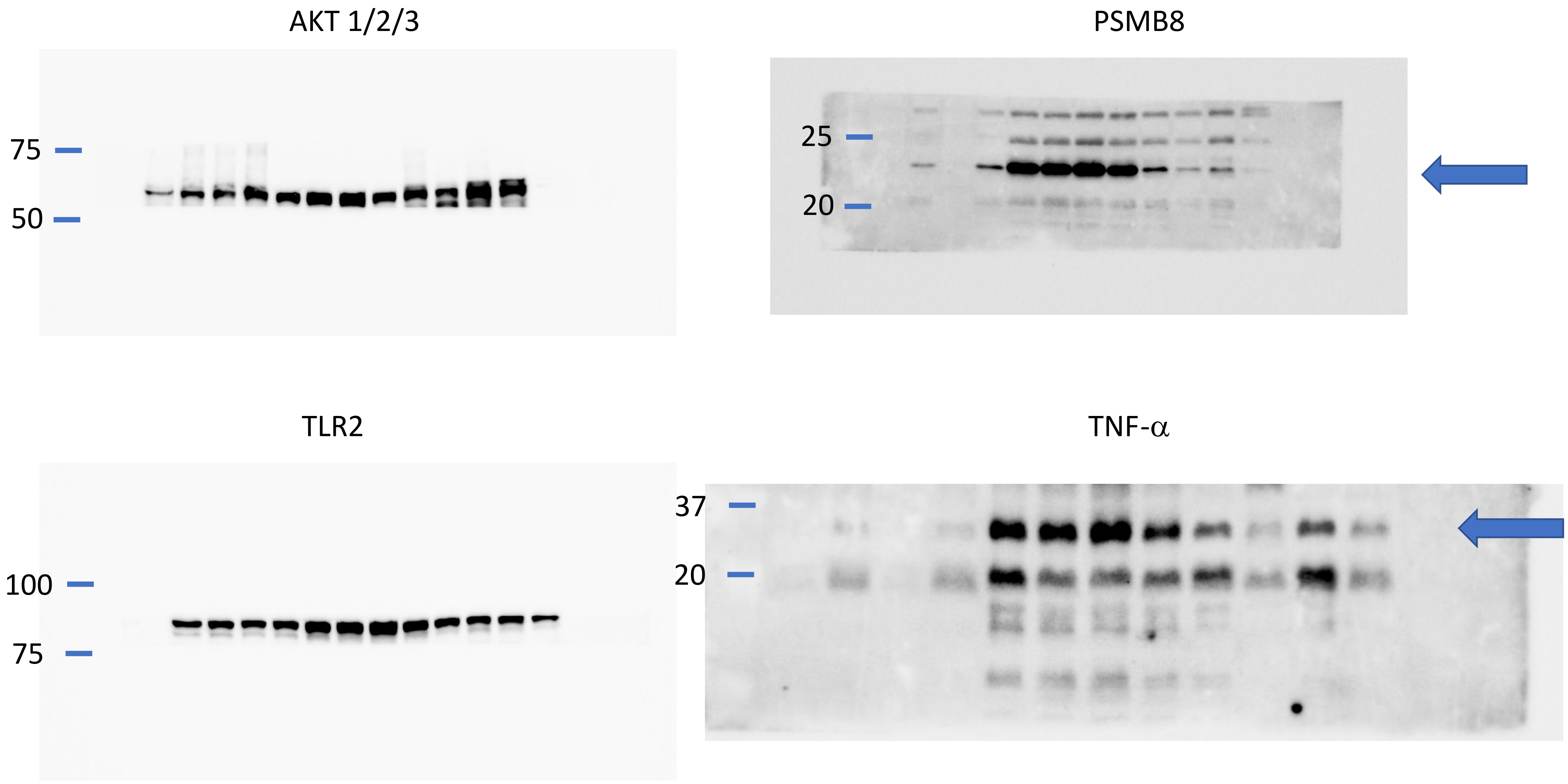

FOXO1

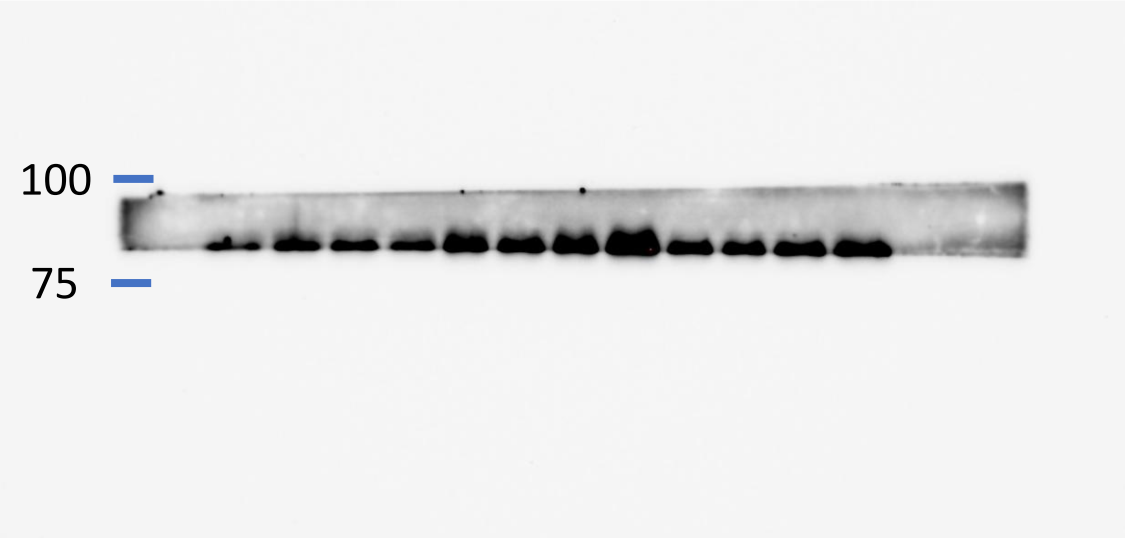

IL-6

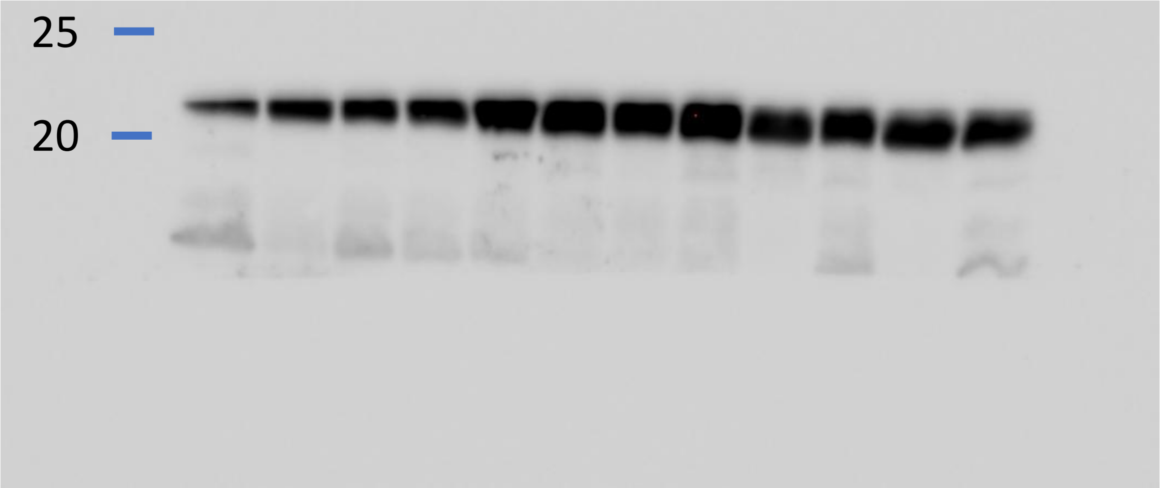

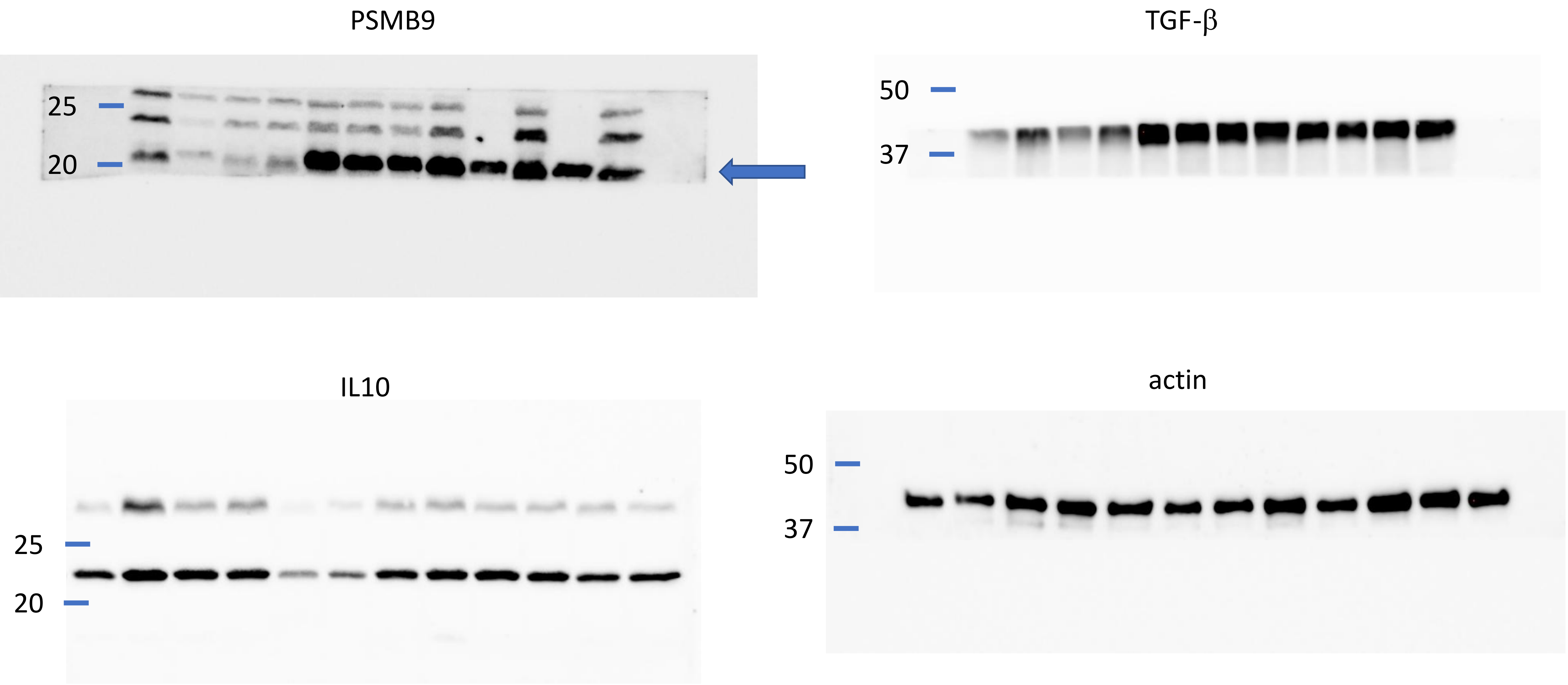
PSMB9
25
20
TGF-β
50
37
IL10
25
20
actin
50
37

Figure S1C - BIS

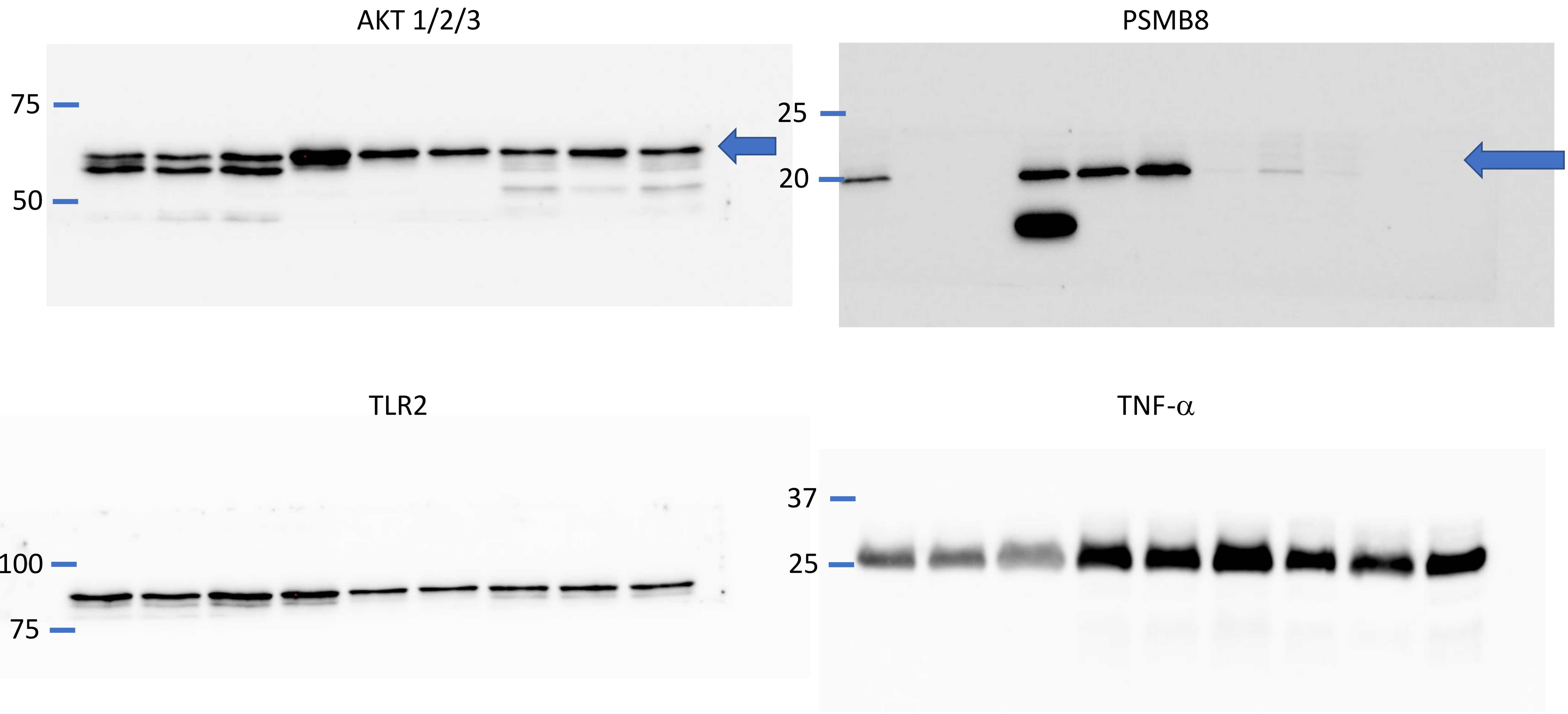

FOXO1

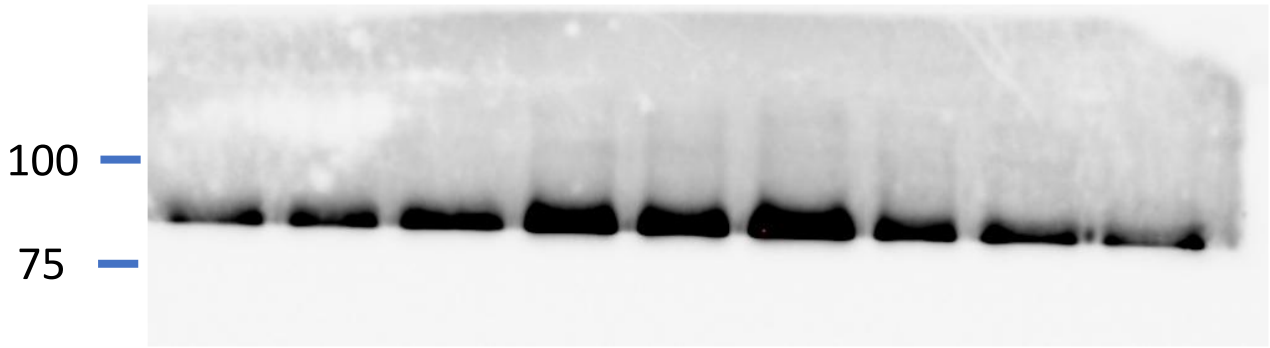

IL-6

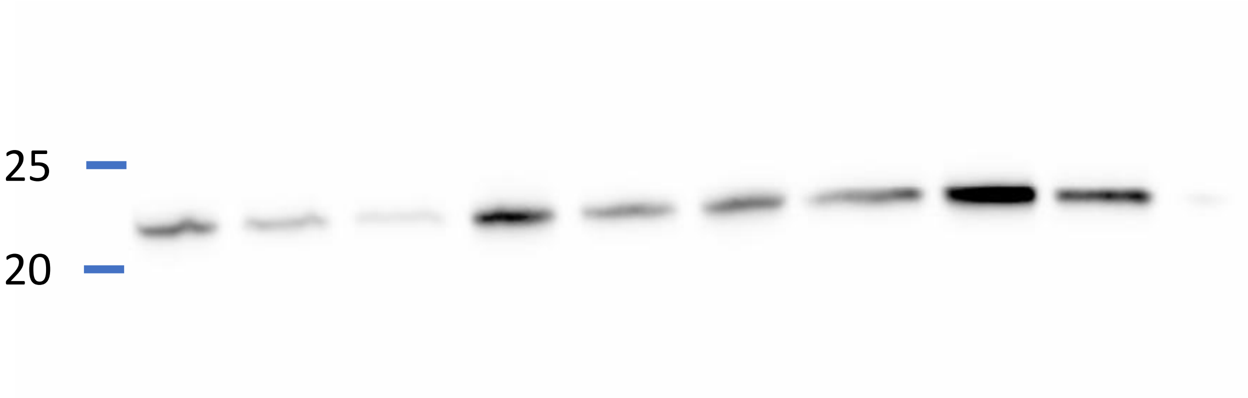

IL-10

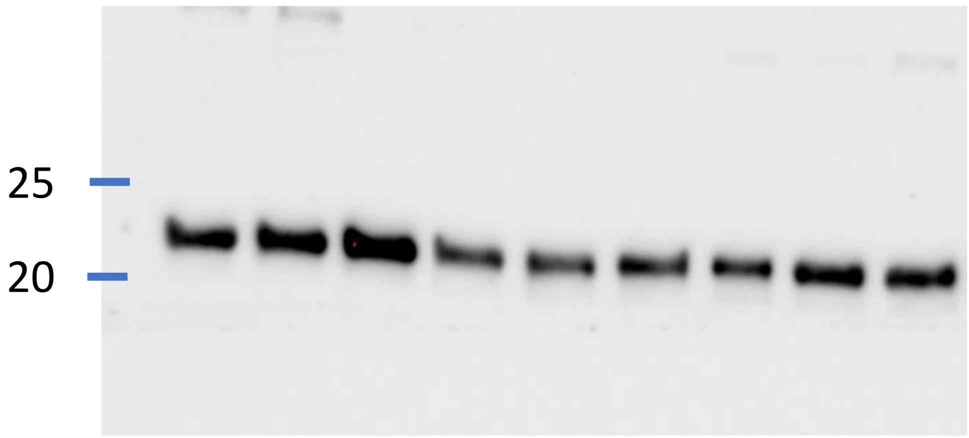

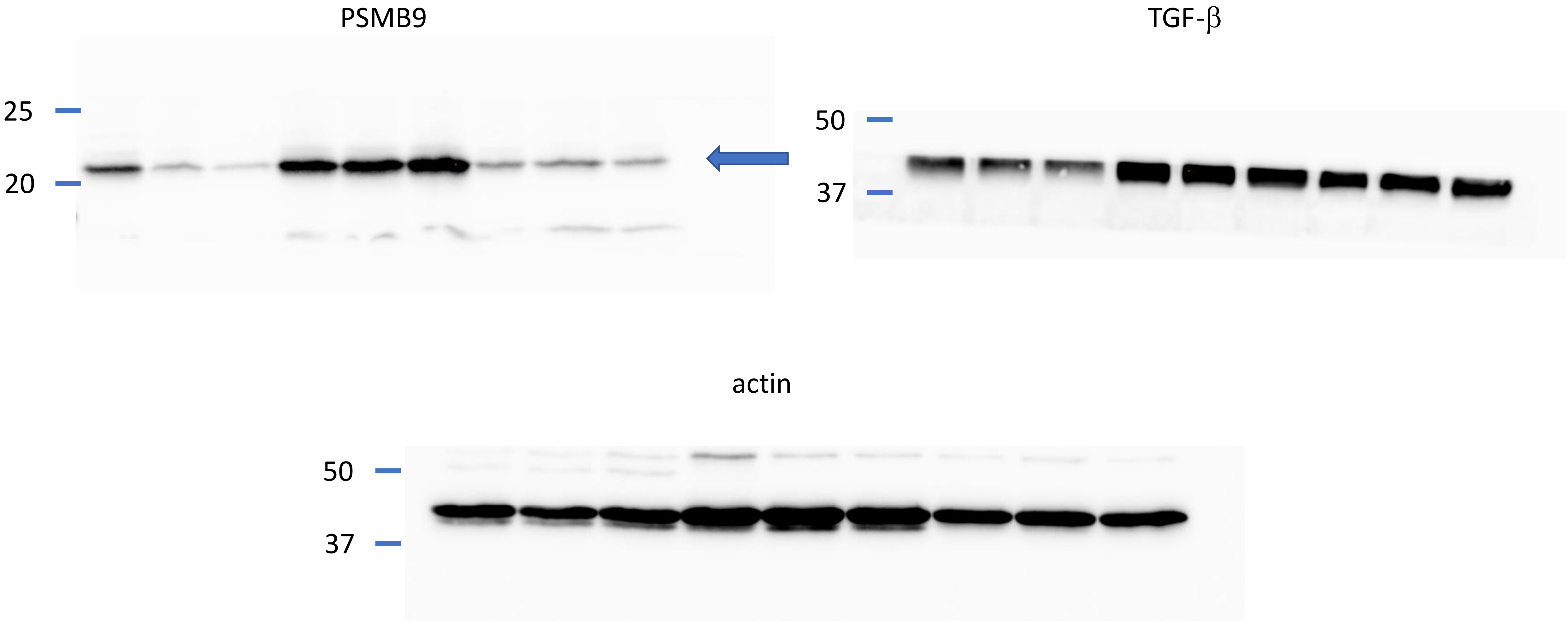
PSMB9
25
20
TGF-β
50
37
actin
50
37

FIGURE S1D

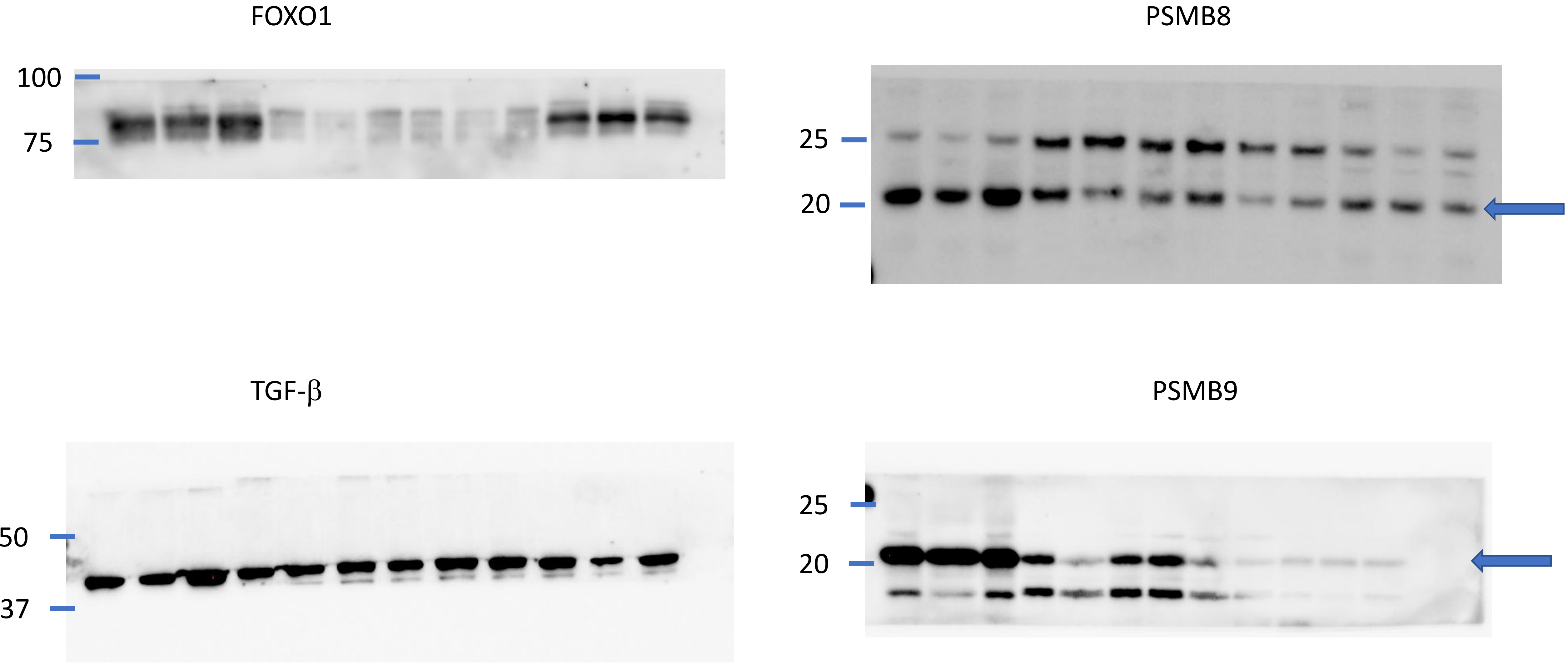

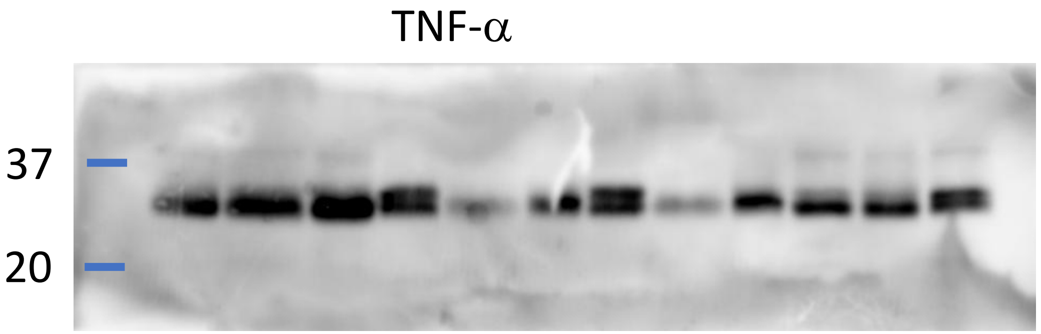
TNF-α
37
20

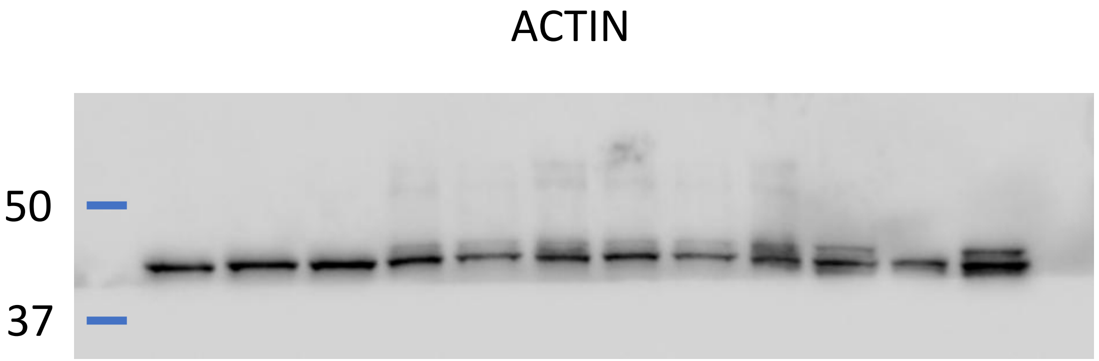
ACTIN
50
37

FIGURE S1D bis

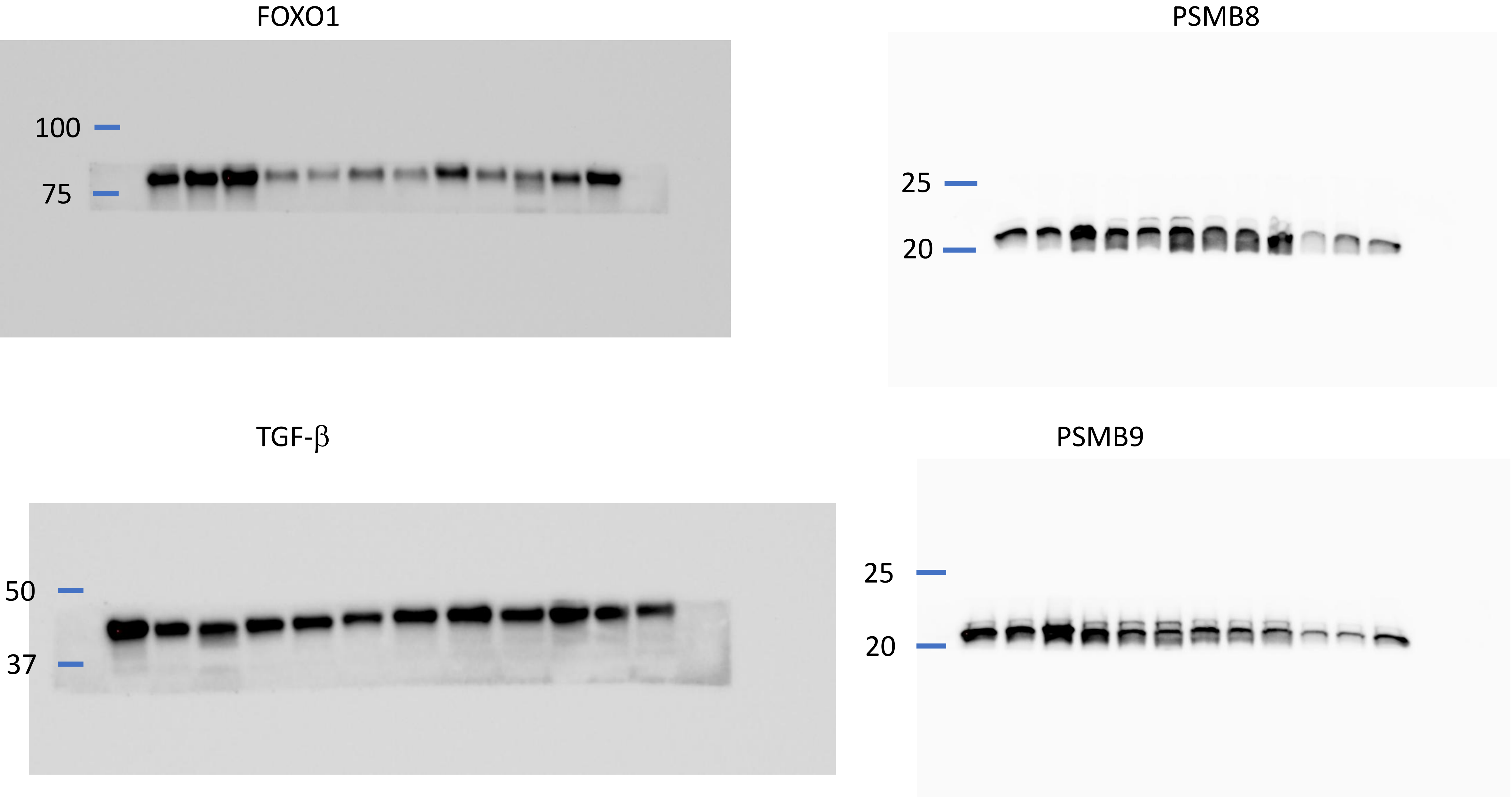

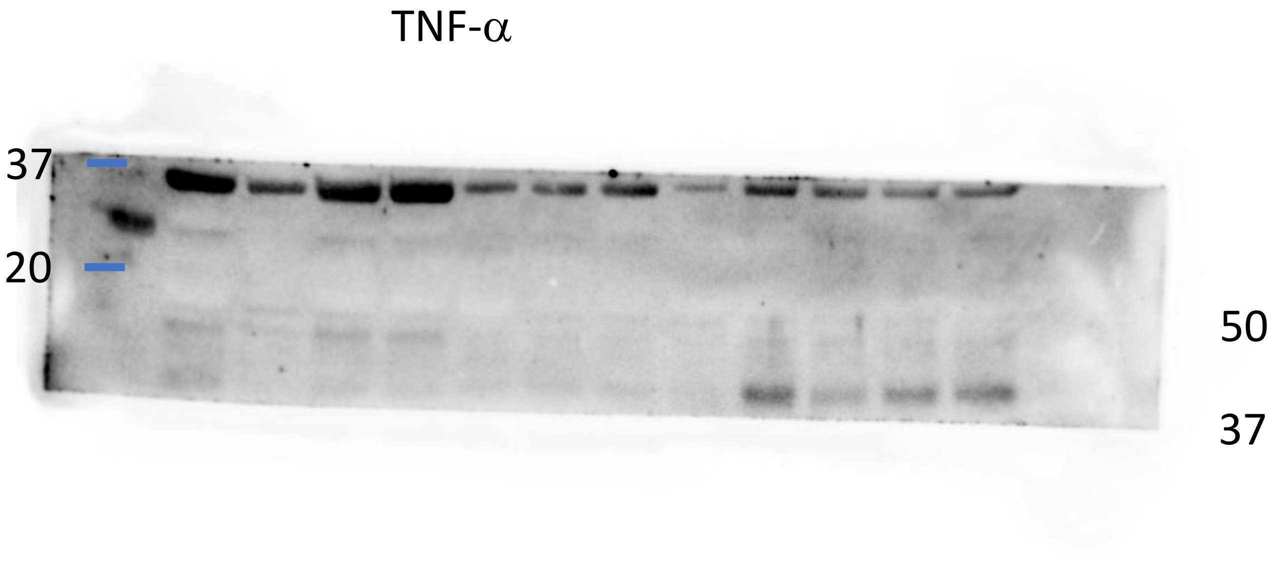
TNF-α
37
20

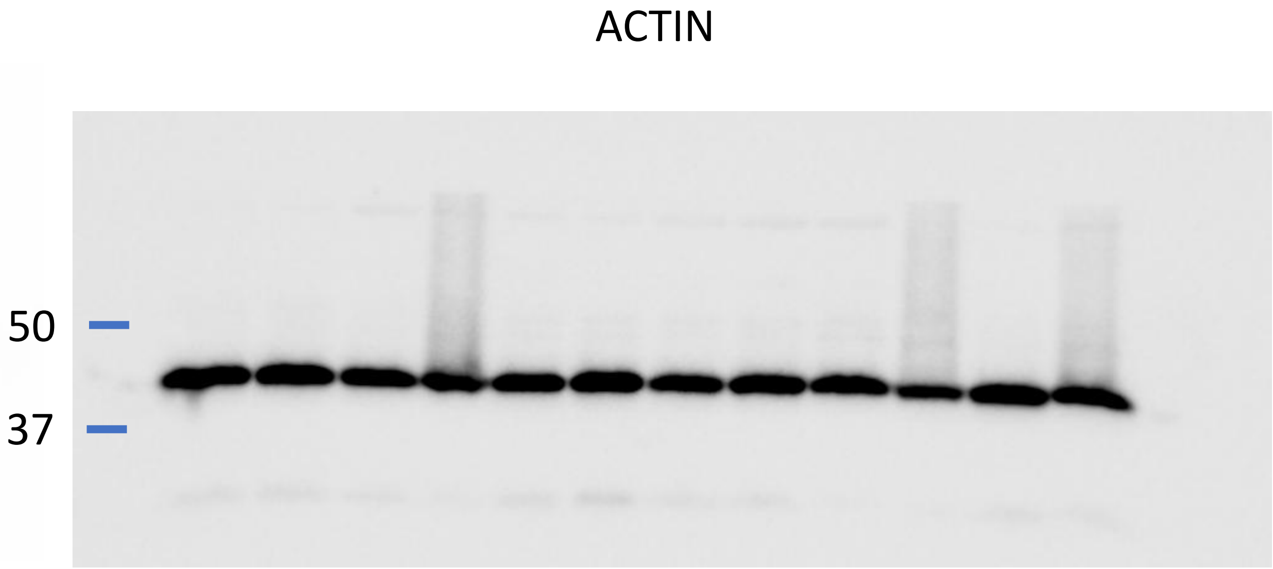
ACTIN
50
37

Figure S5A

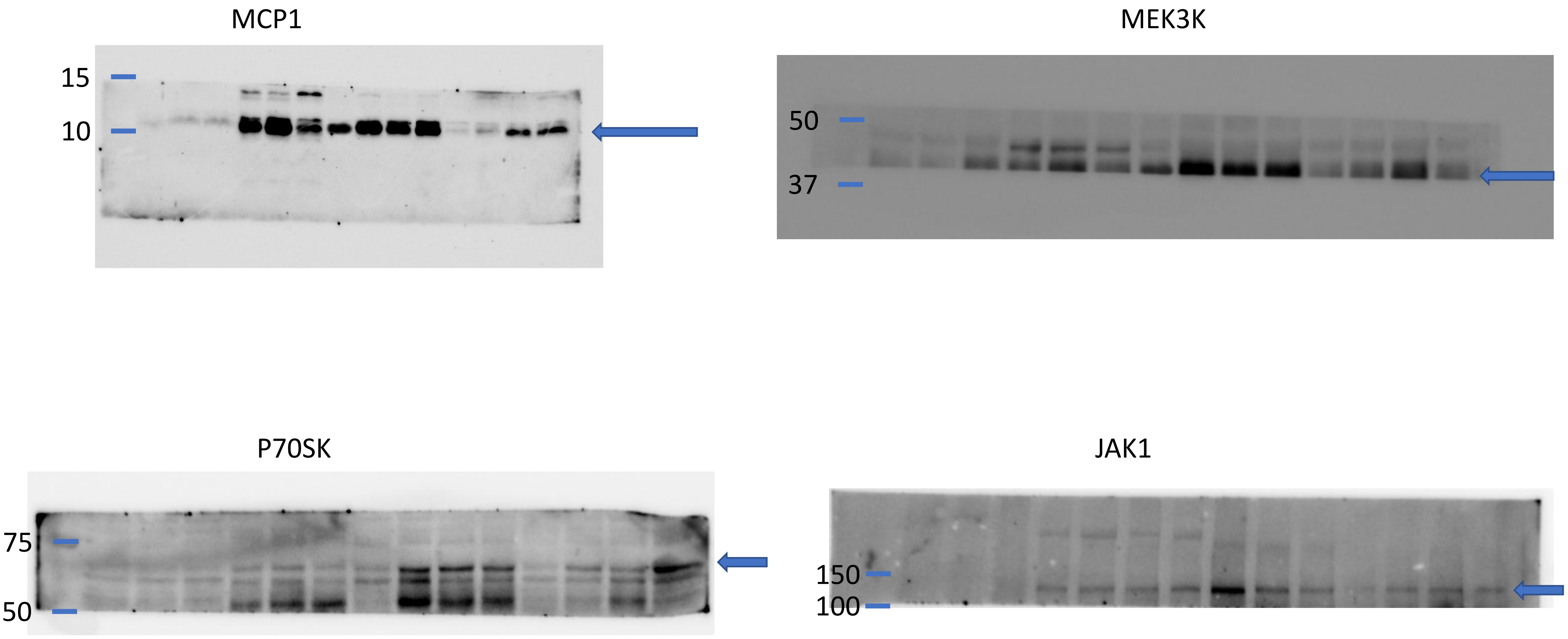

S6RB

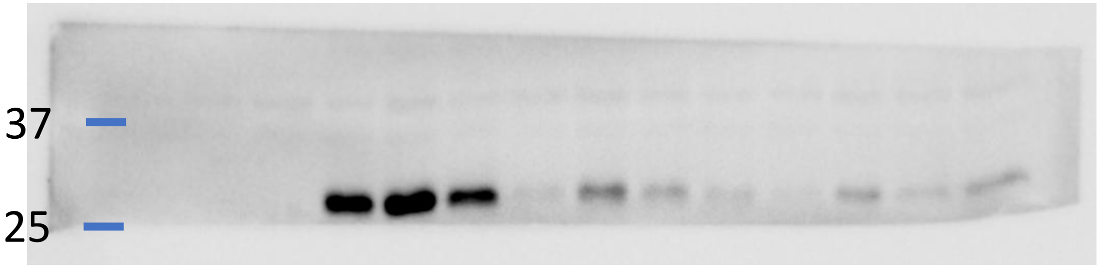

4EBP1

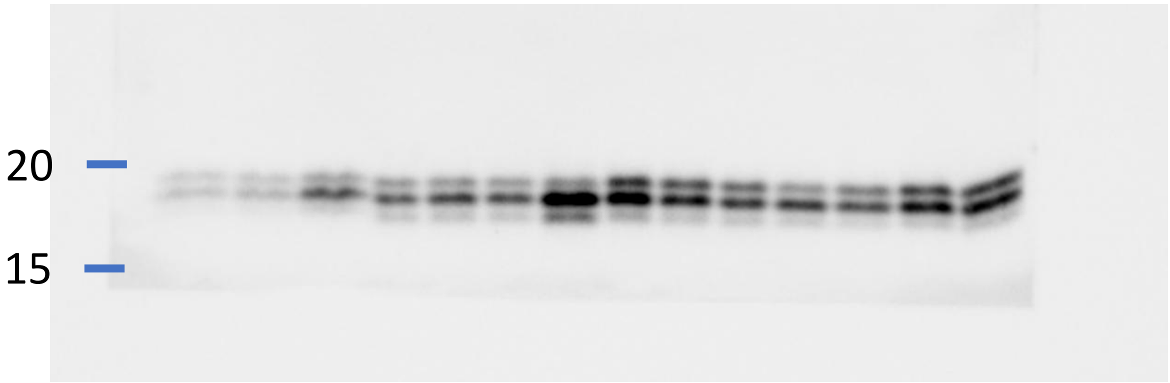

MTOR

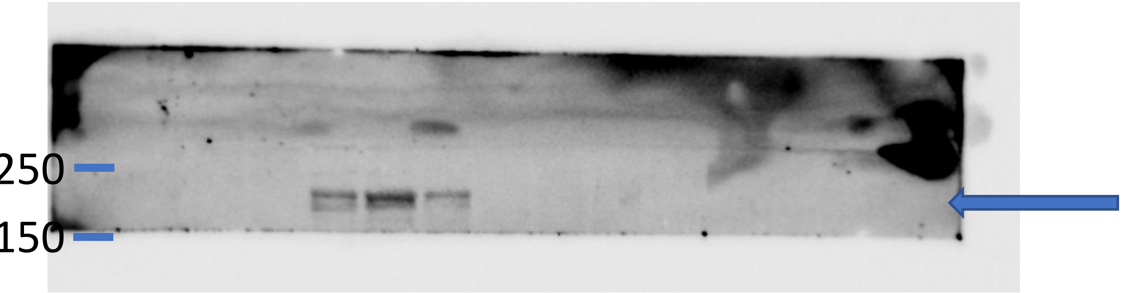

PHO-4EBP1

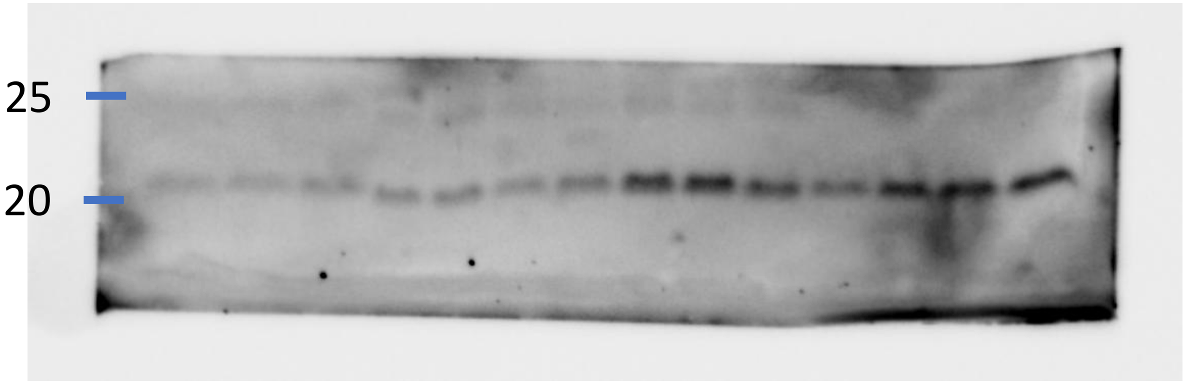

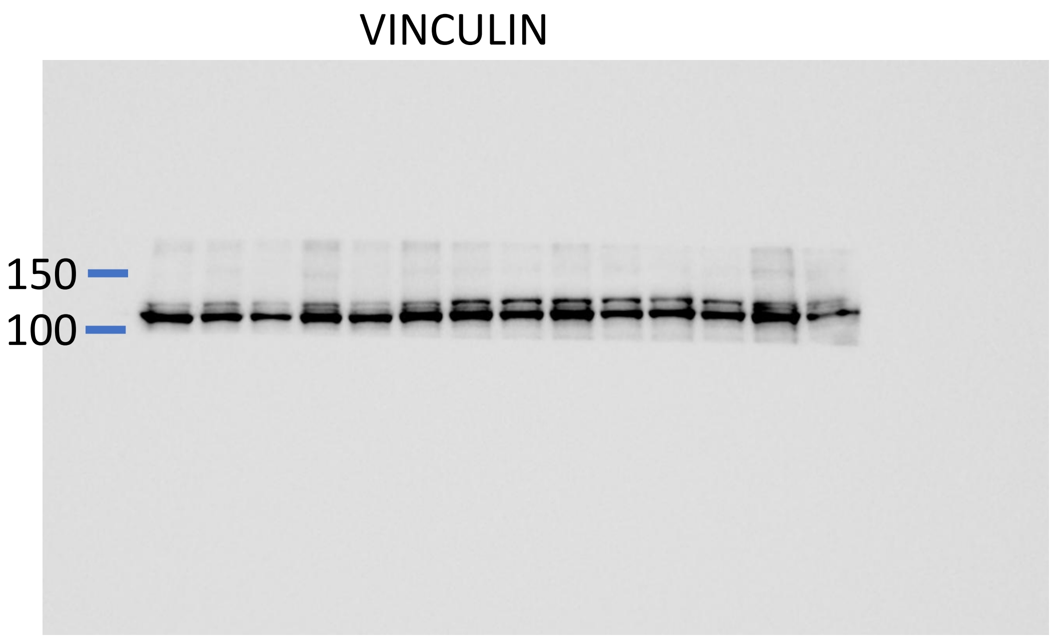
VINCULIN
150
100

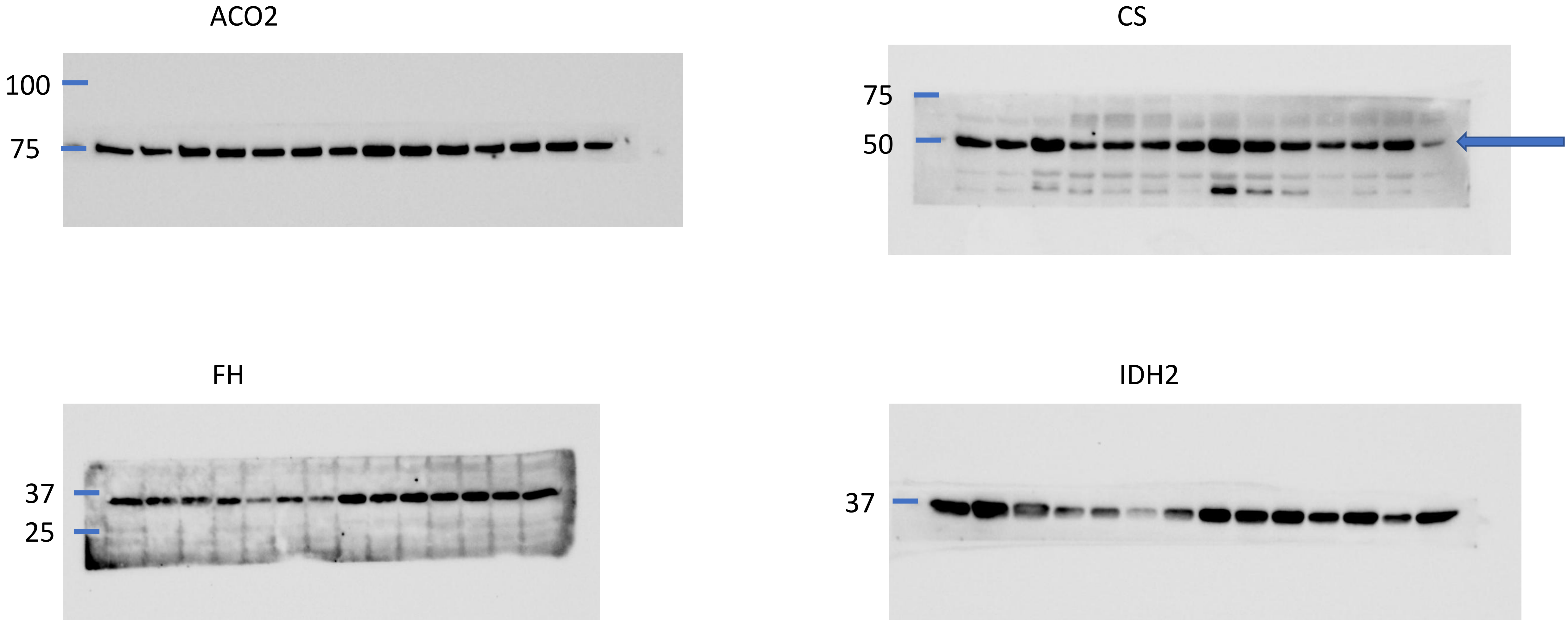
ACO2
100
75
CS
75
50
FH
37
25
IDH2
37

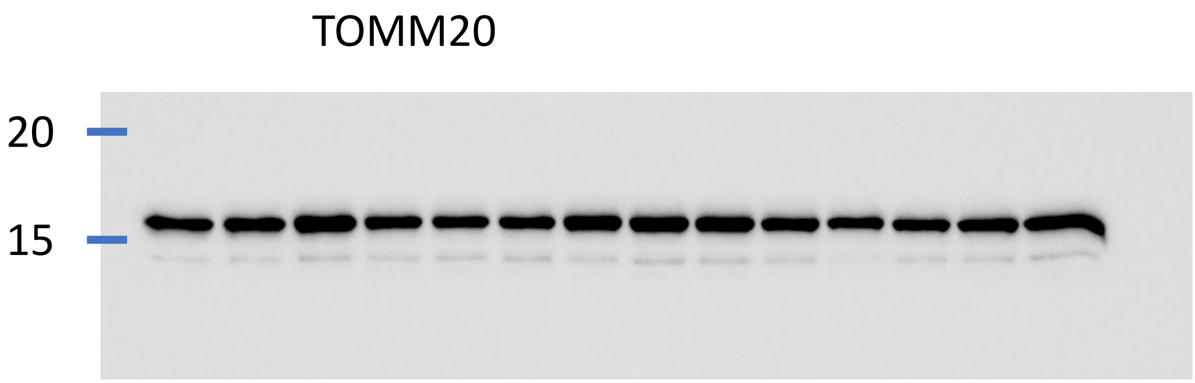
TOMM20
20
15

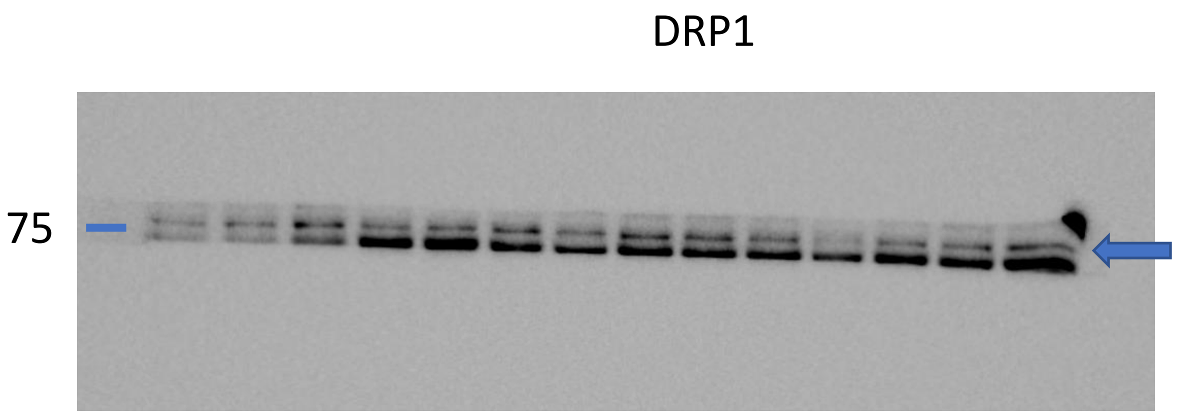
DRP1
75

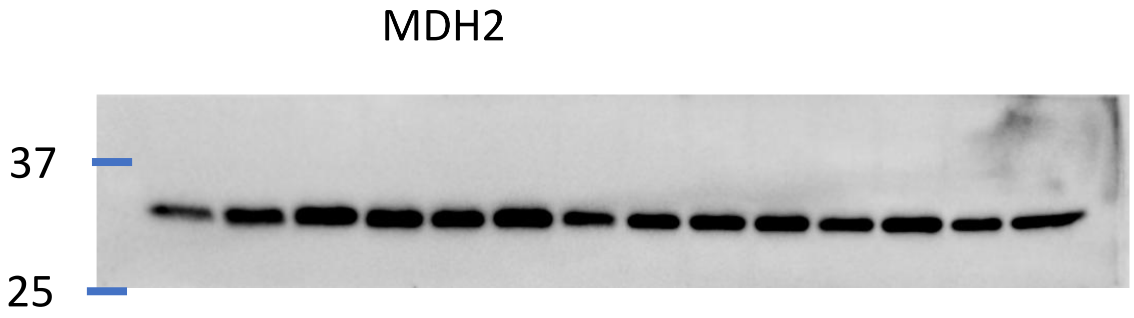
MDH2
37
25

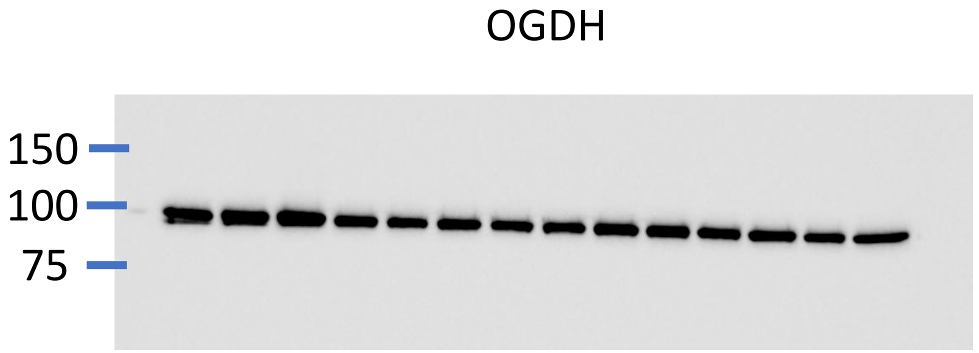
OGDH
150
100
75

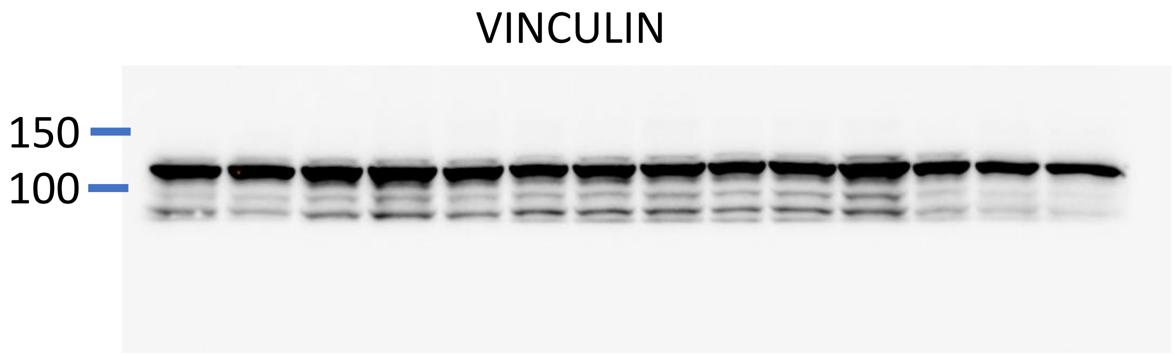
VINCULIN
150
100

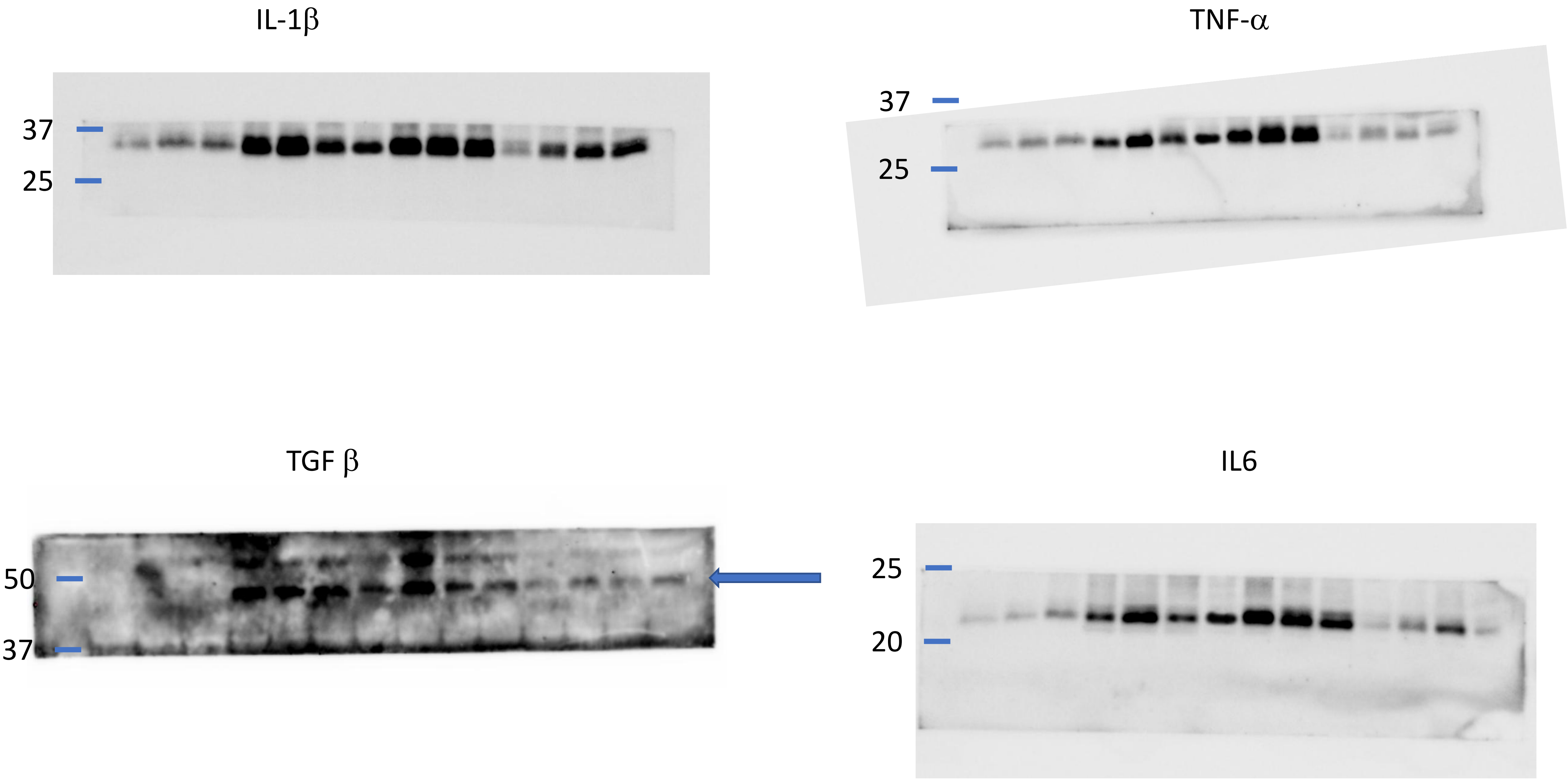
IL-1β
37
25
TNF-α
37
25
TGF β
50
37
IL6
25
20

NFkB

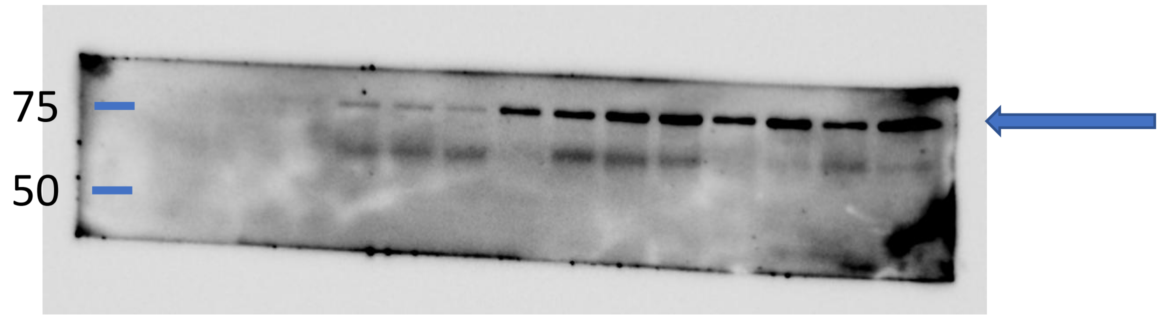

HMGB1

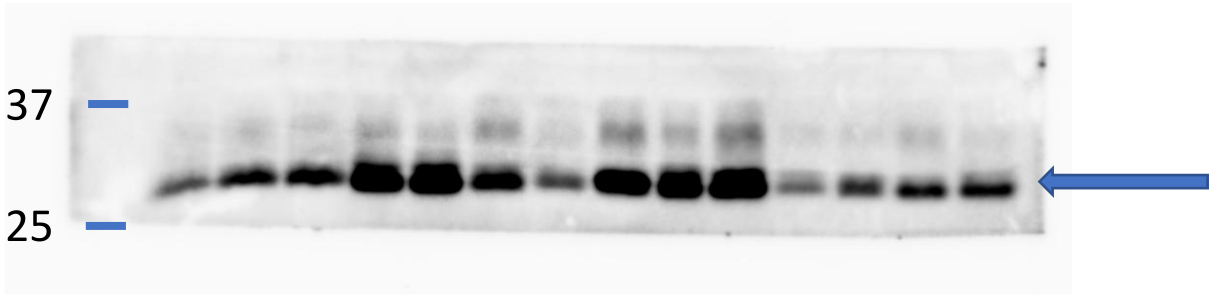

Vinculin

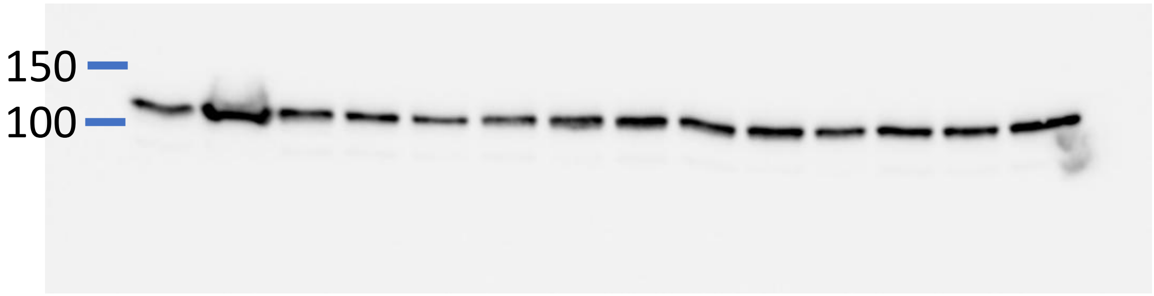

OXPHOS

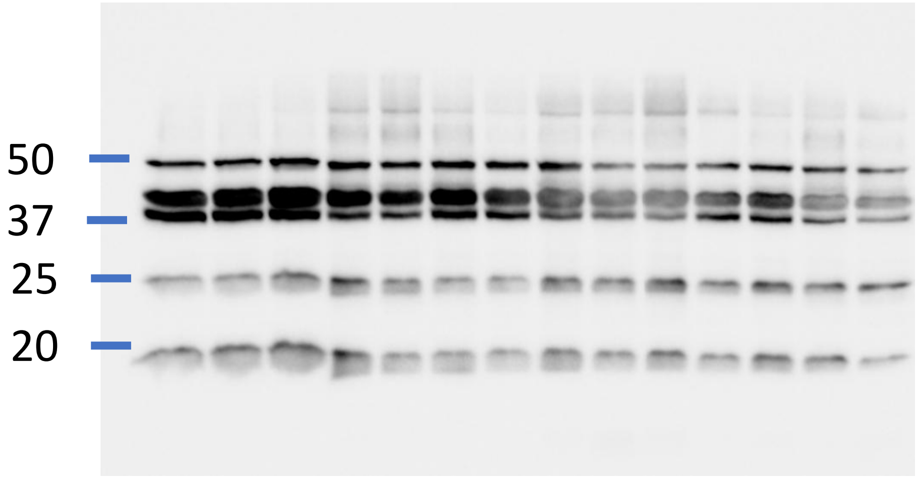

vinculin

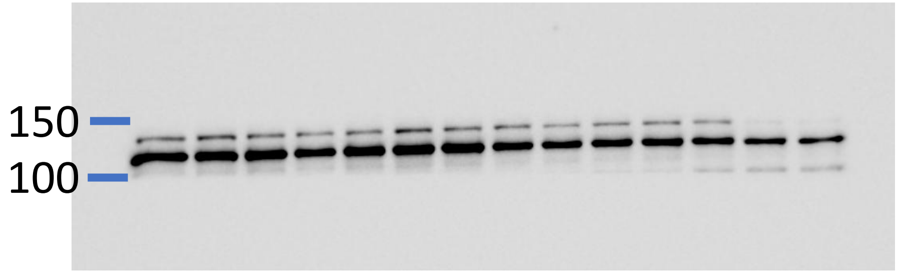

Figure S5B

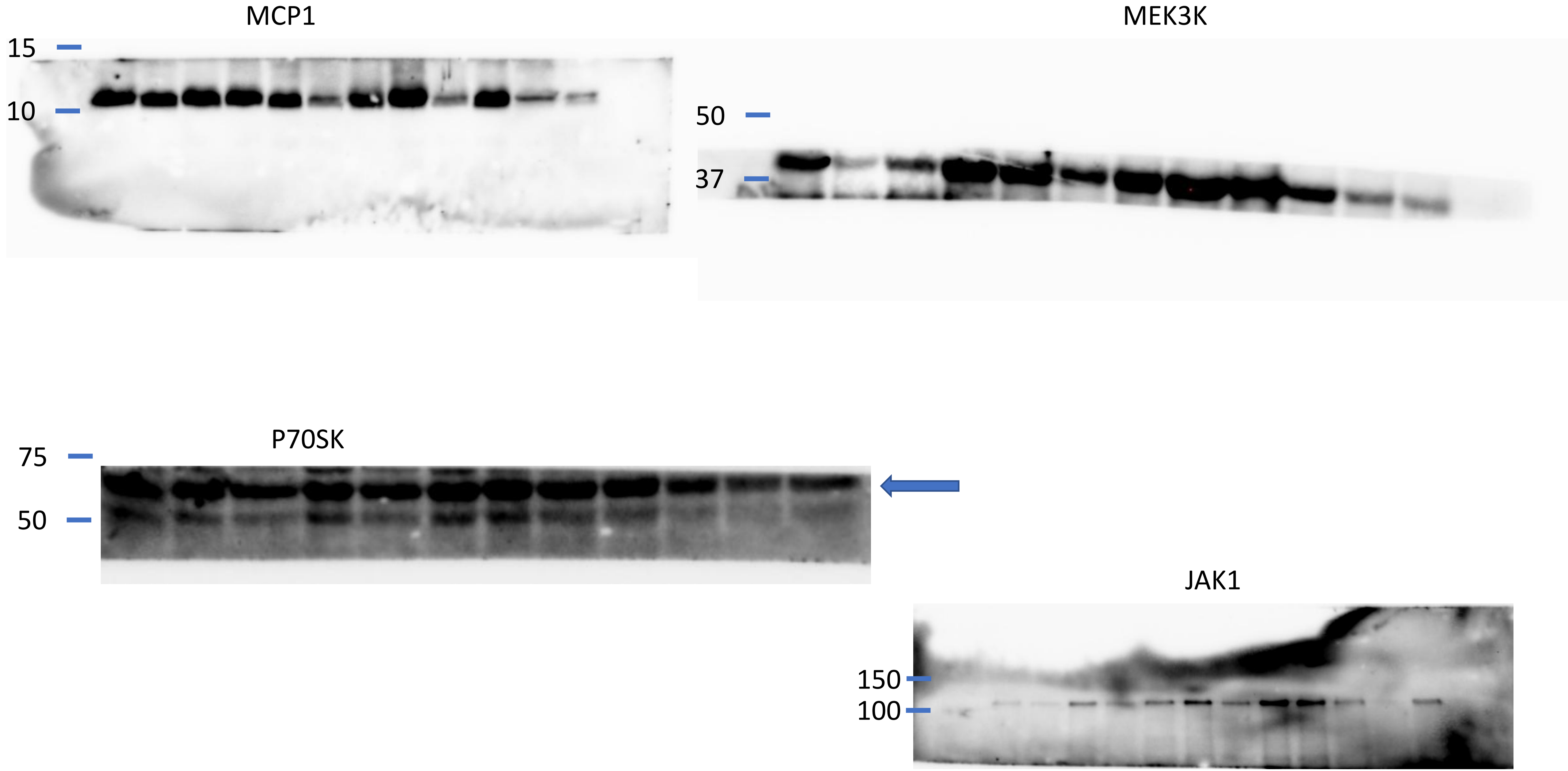

S6RB

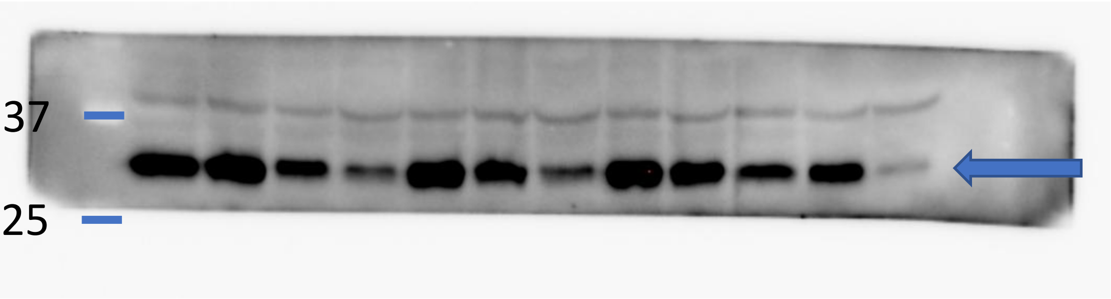

4EBP1

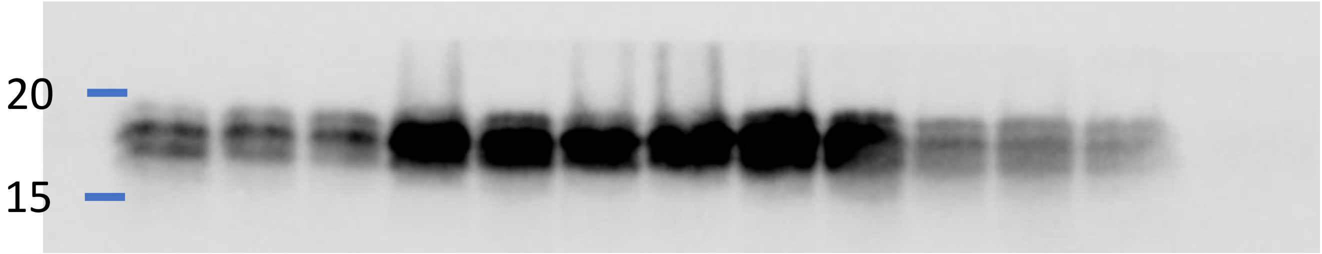

MTOR

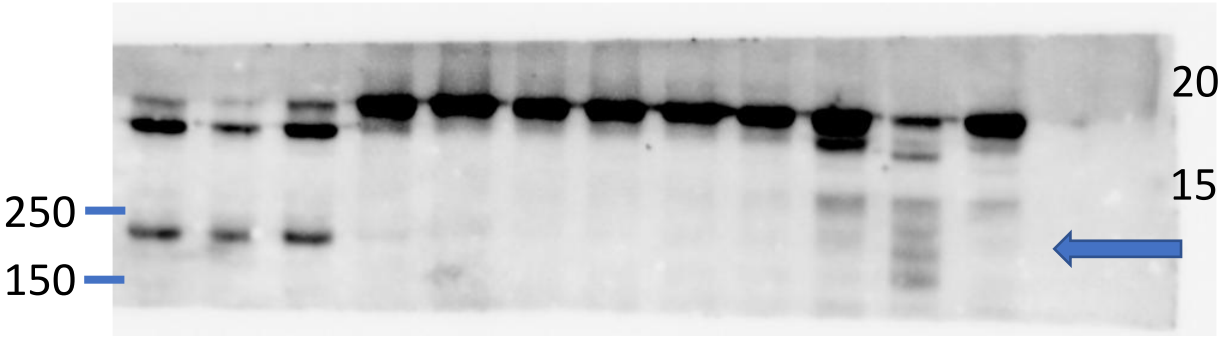

PHO-4EBP1

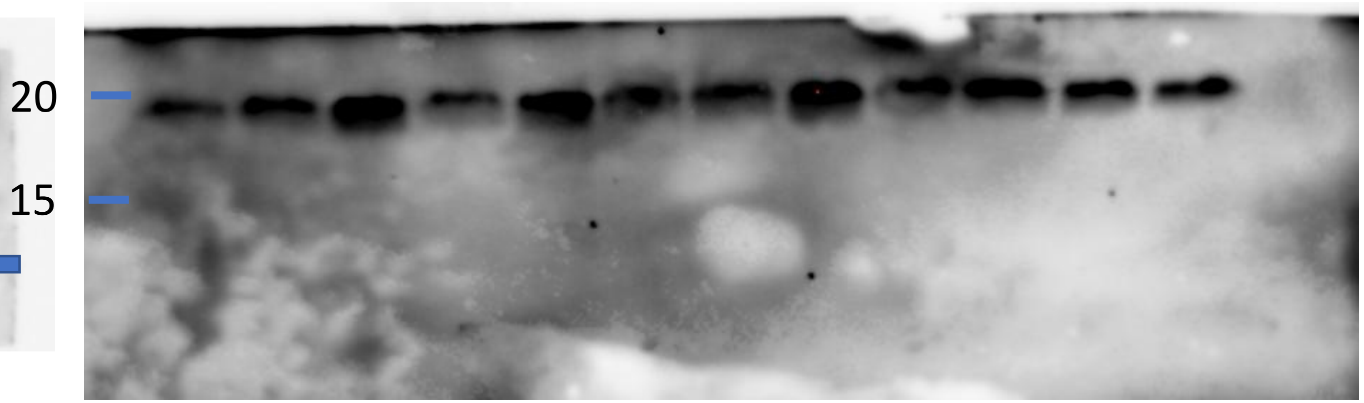

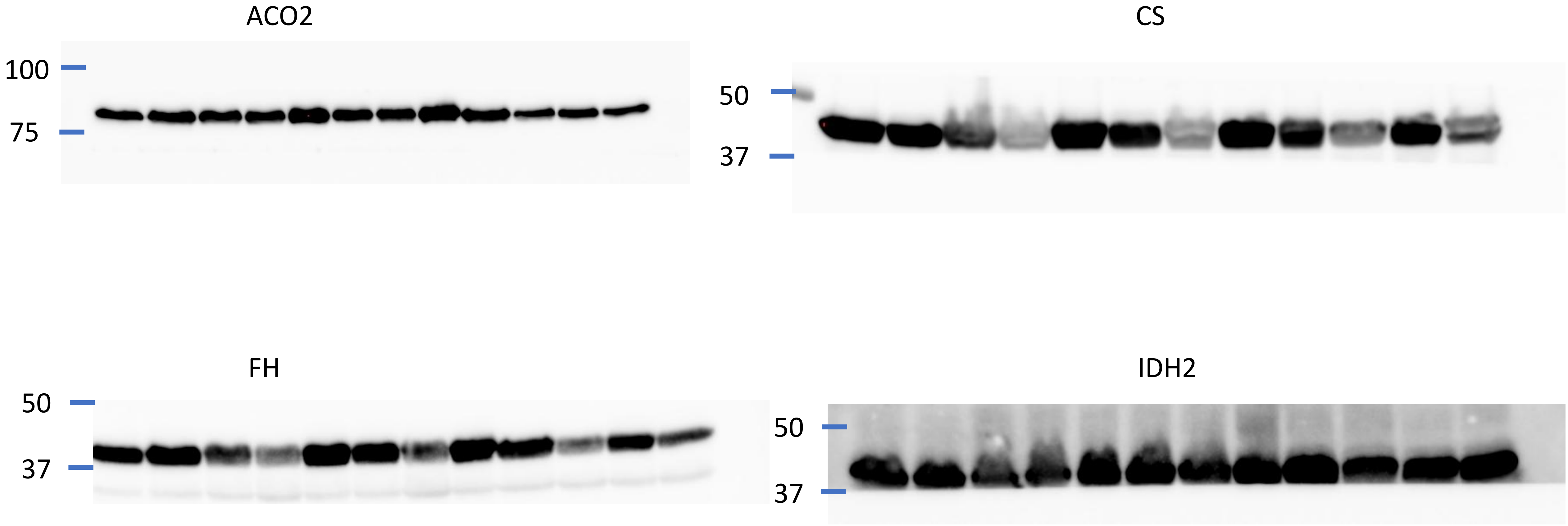
ACO2
100
75
CS
50
37
FH
50
37
IDH2
50
37

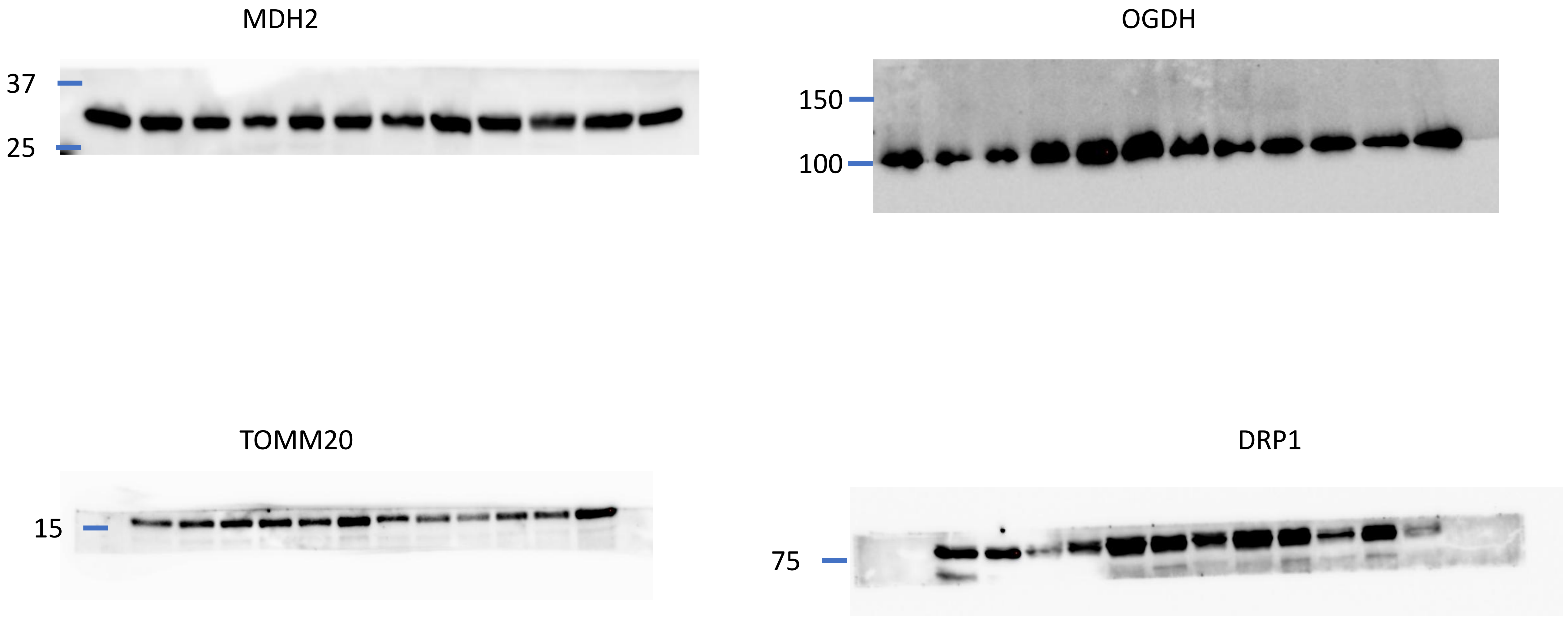
MDH2
37
25
OGDH
150
100
TOMM20
15
DRP1
75

IL-1β

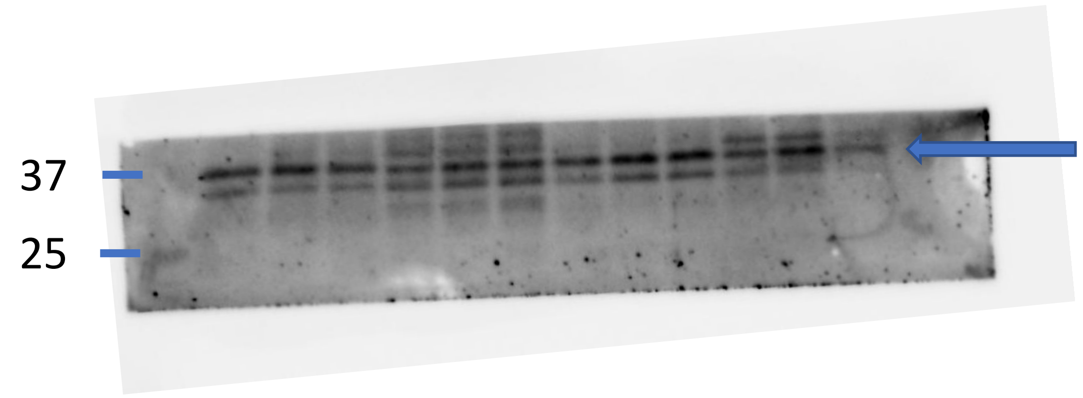

TNF-α

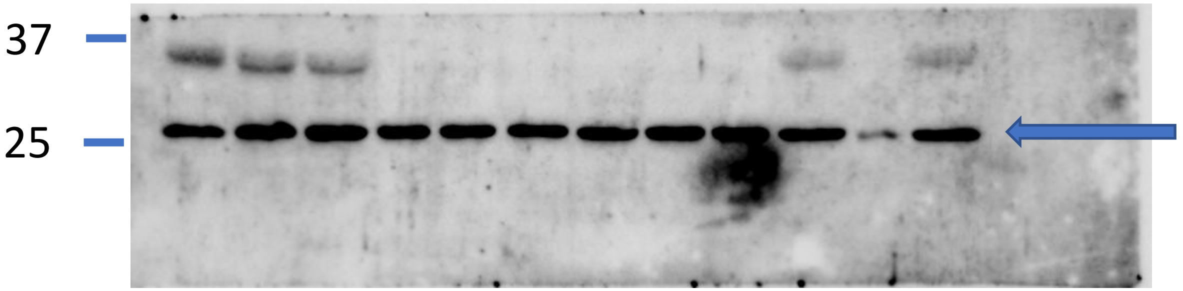

TGF β

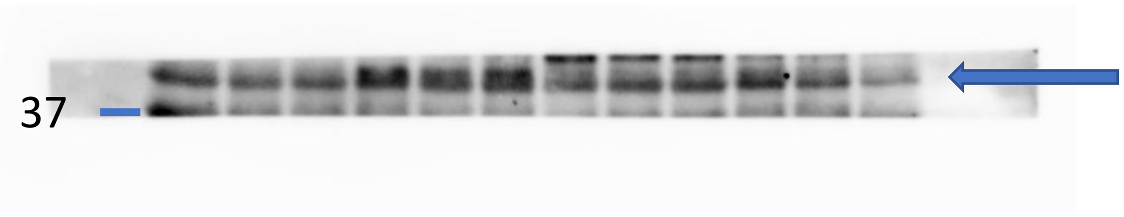

IL6

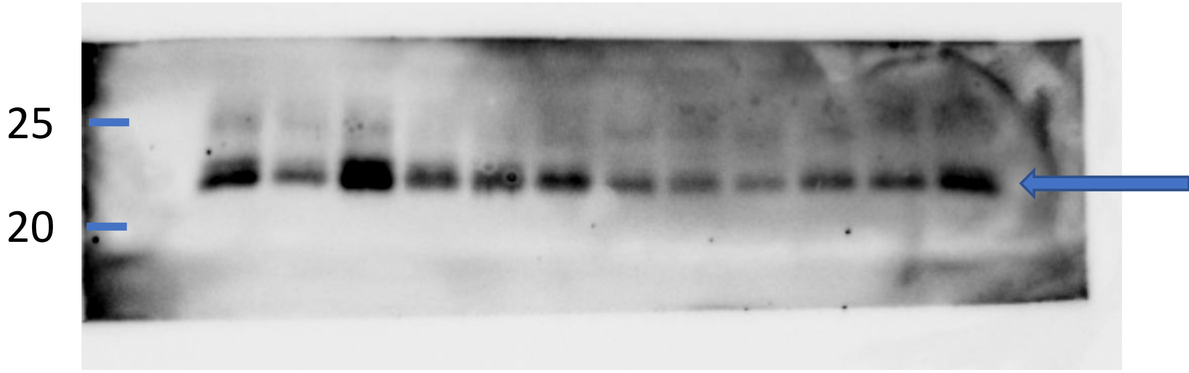

NFkB

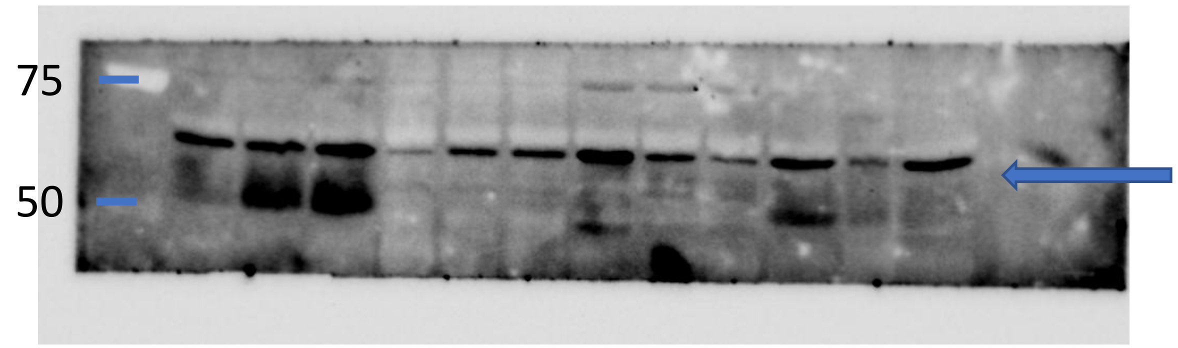

HMGB1

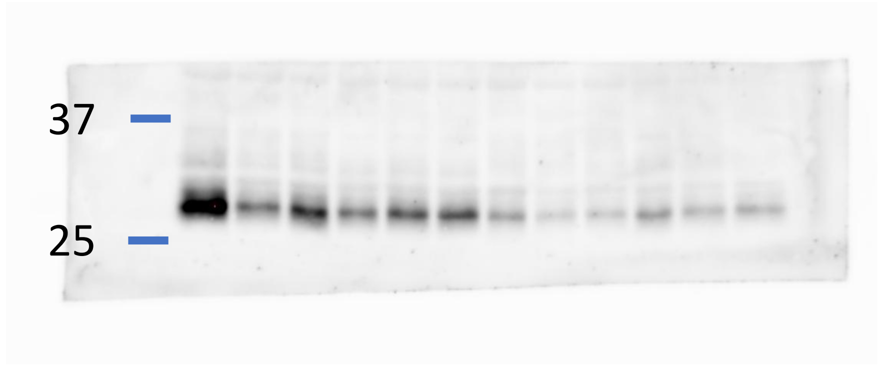

OXPHOS

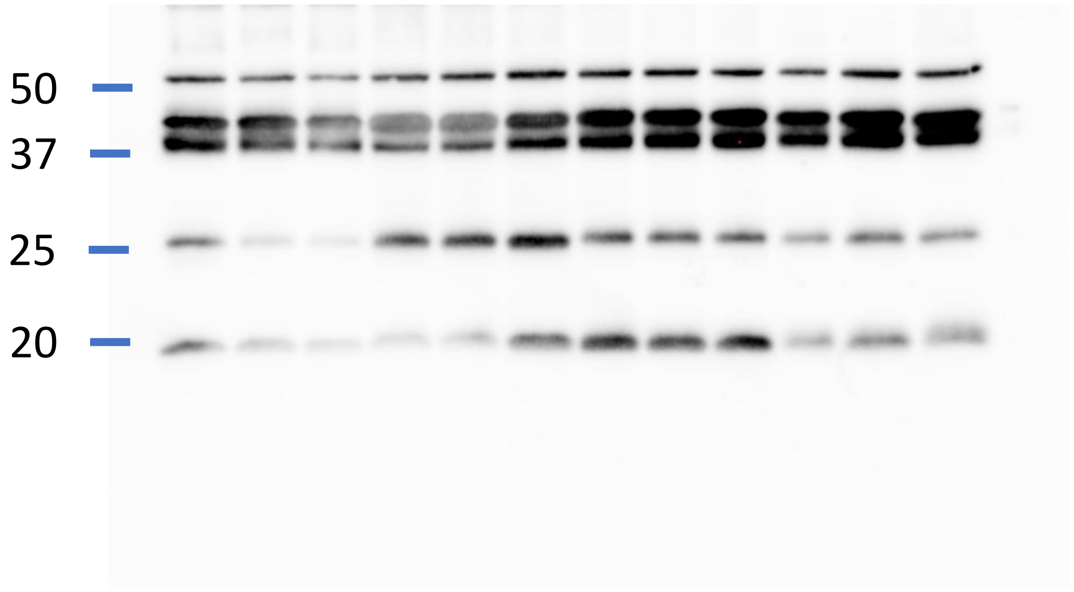

Vinculin

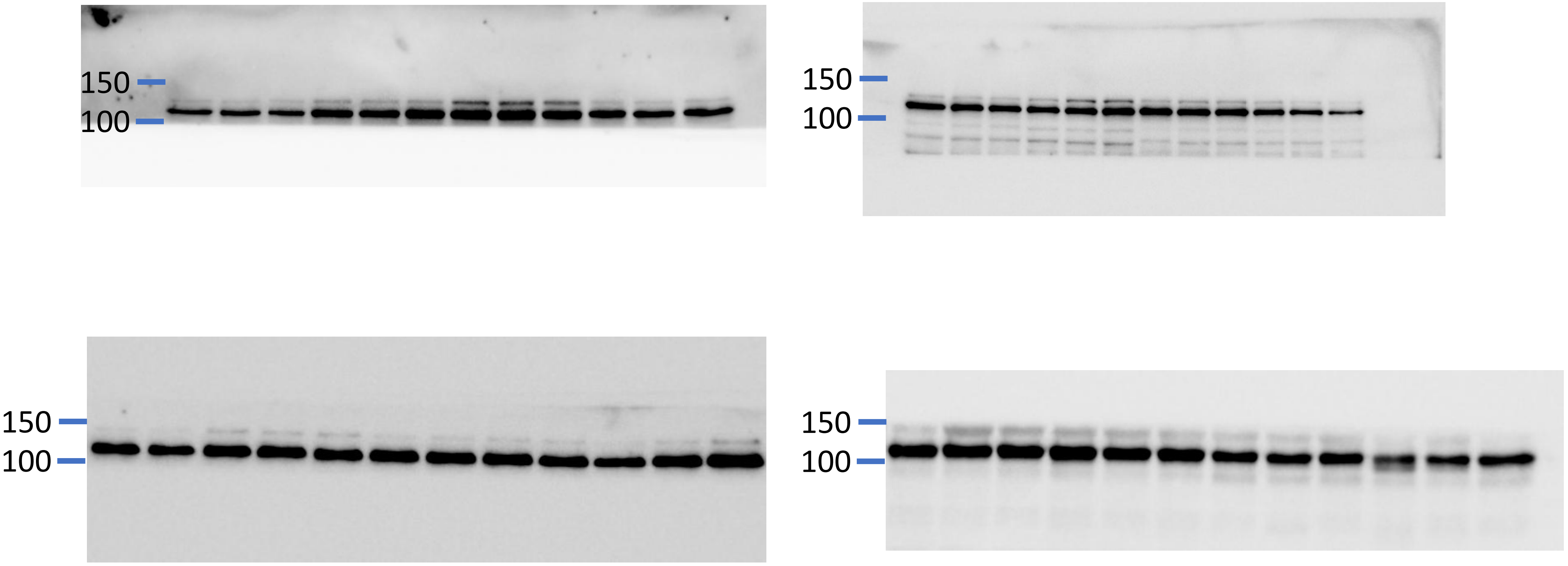

Figure S5B bis

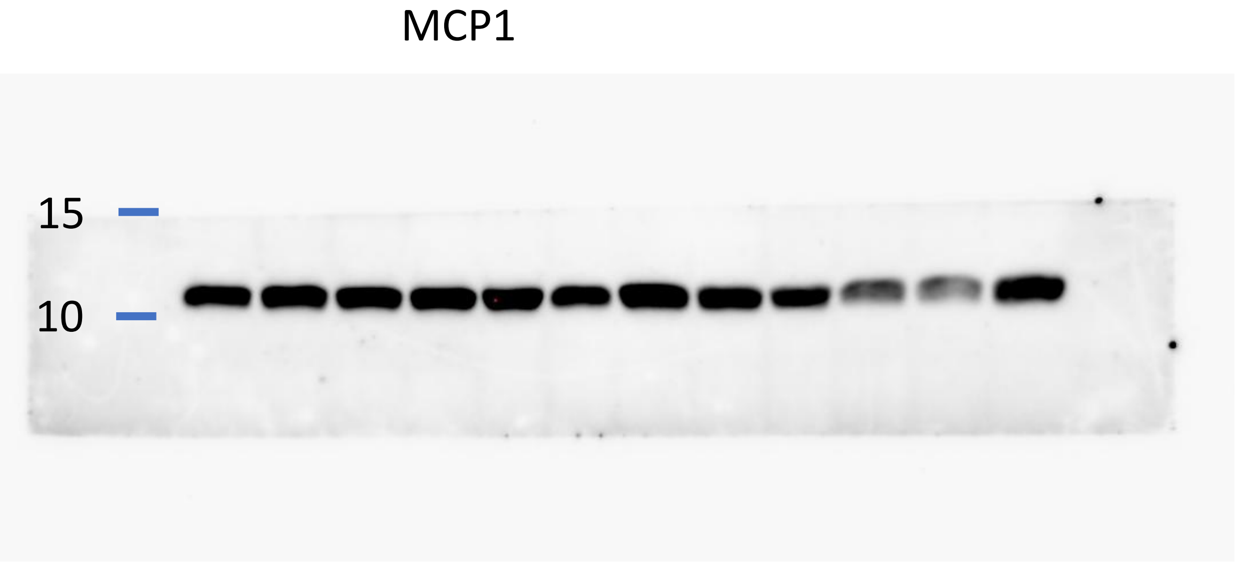

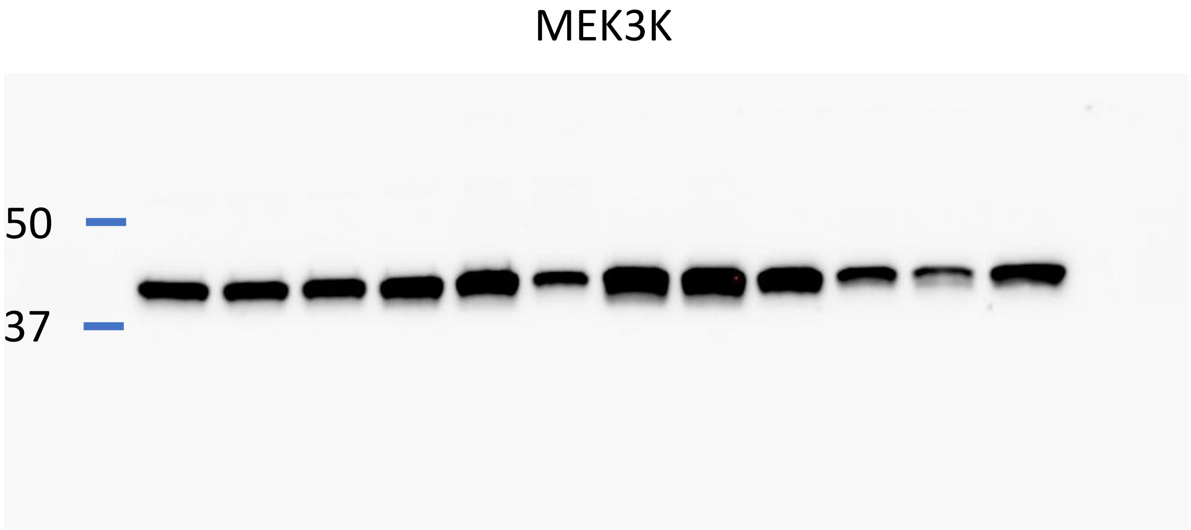

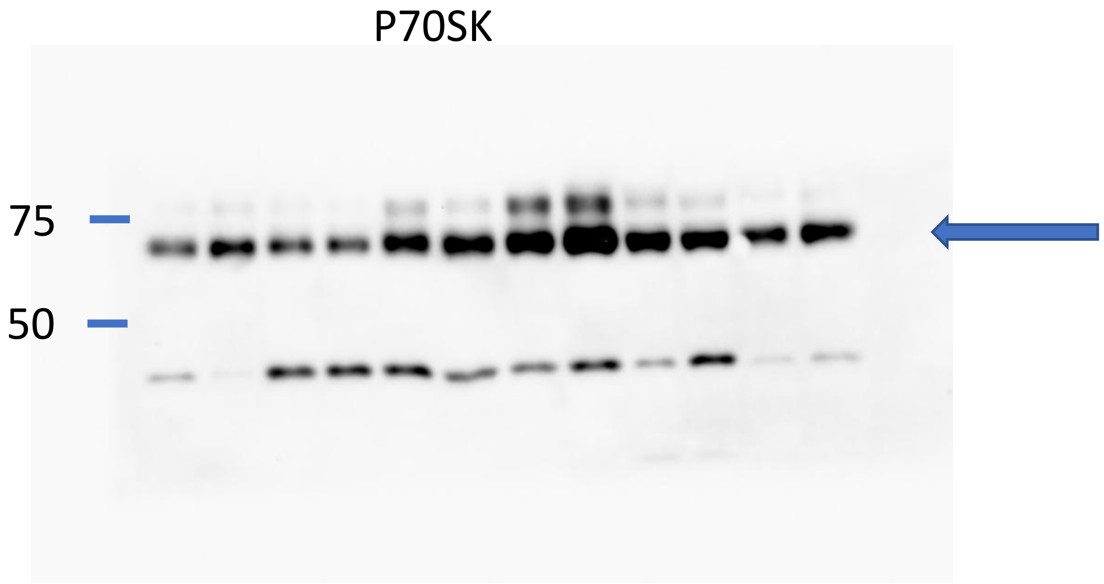

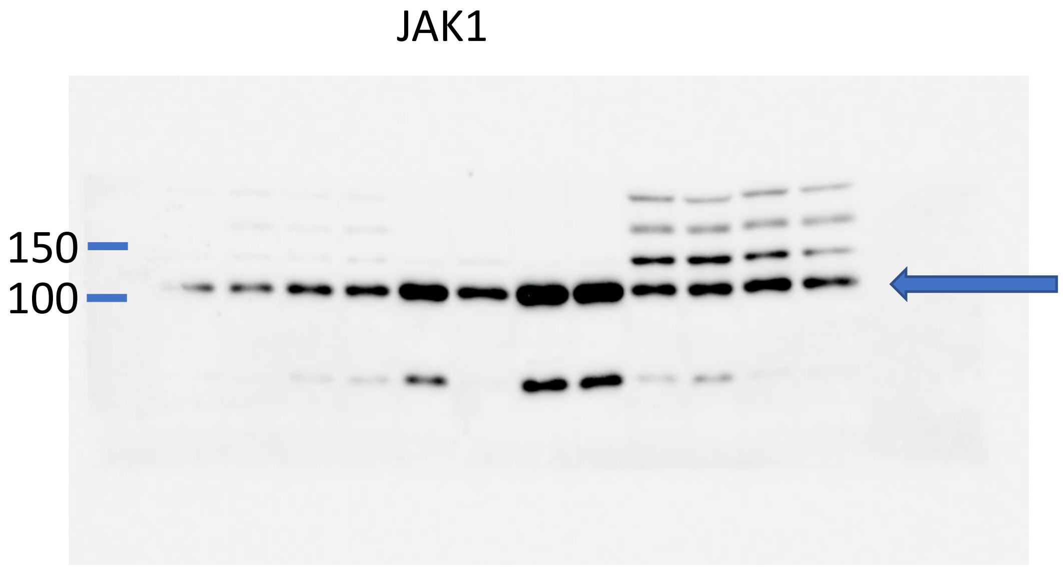

S6RB

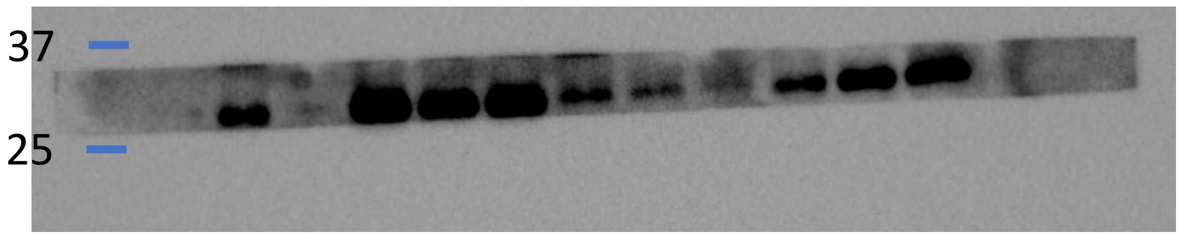

4EBP1

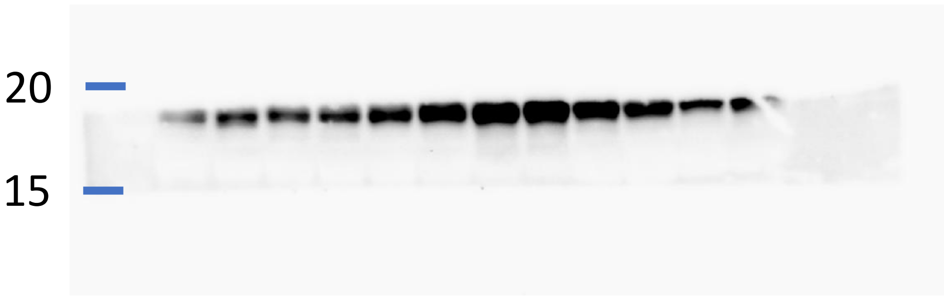

MTOR

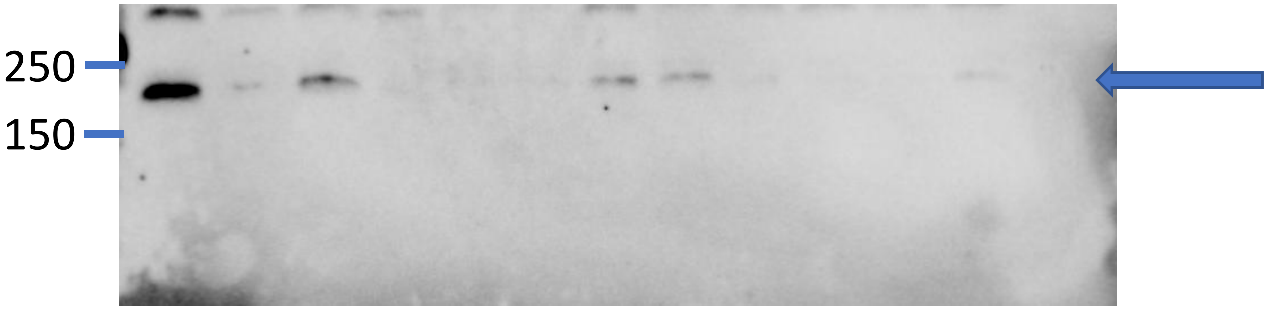

PHO-4EBP1

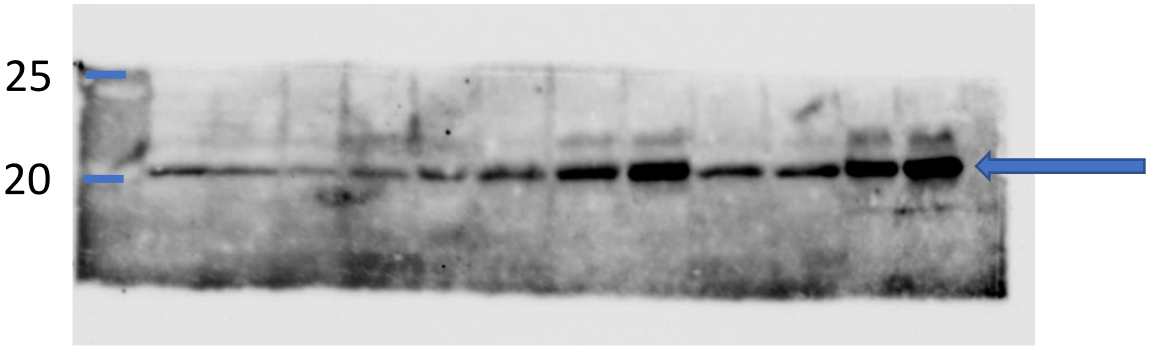

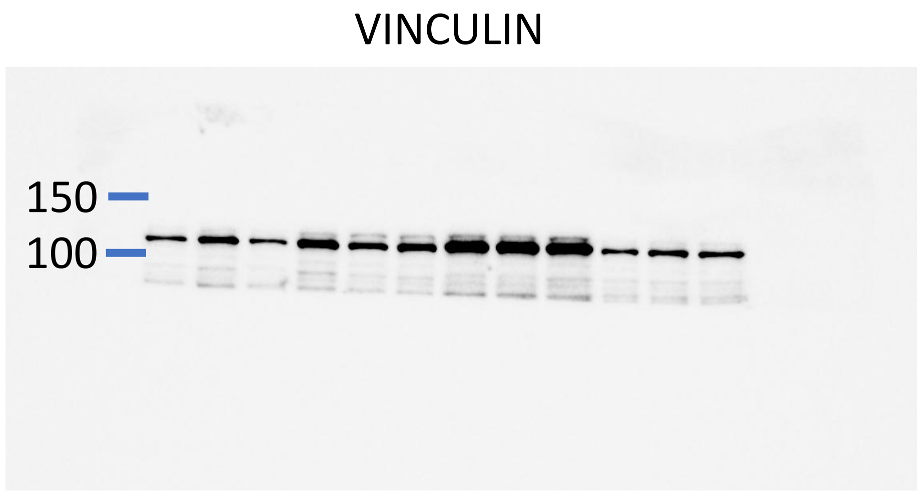
VINCULIN
150
100

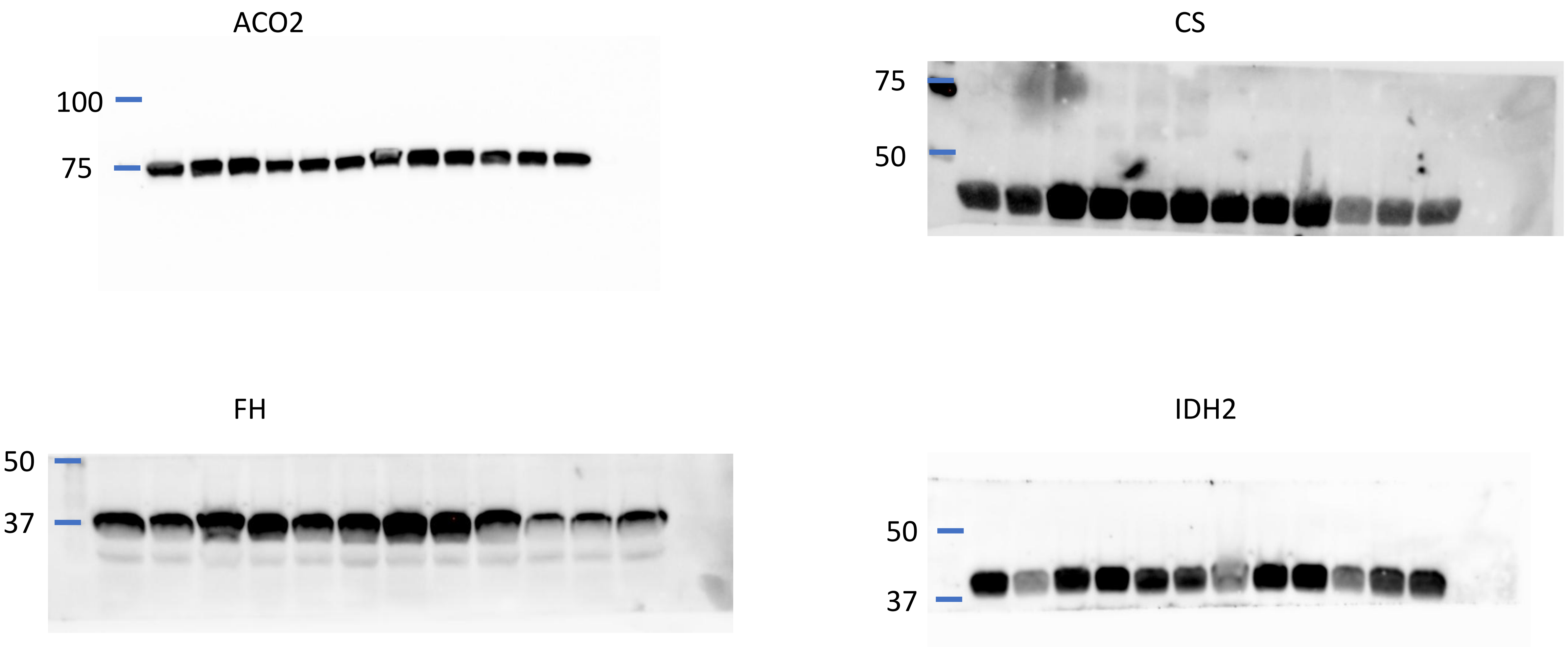
ACO2
100
75
CS
75
50
FH
50
37
IDH2
50
37

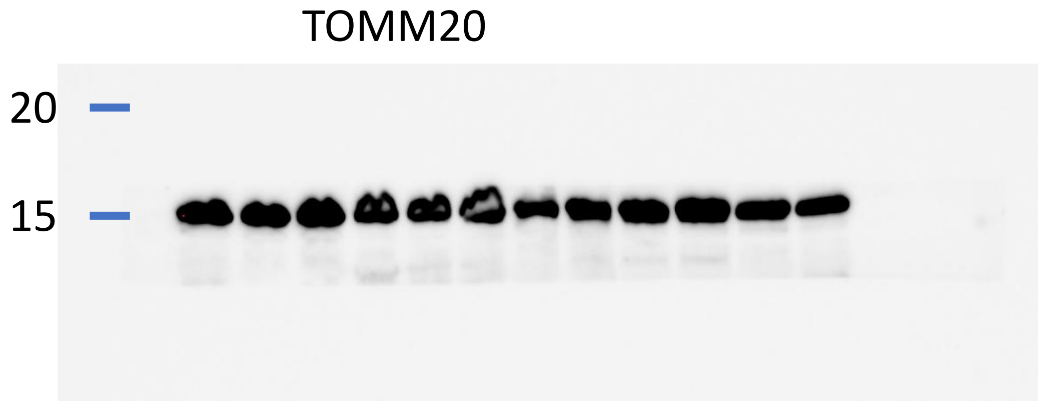
TOMM20
20
15

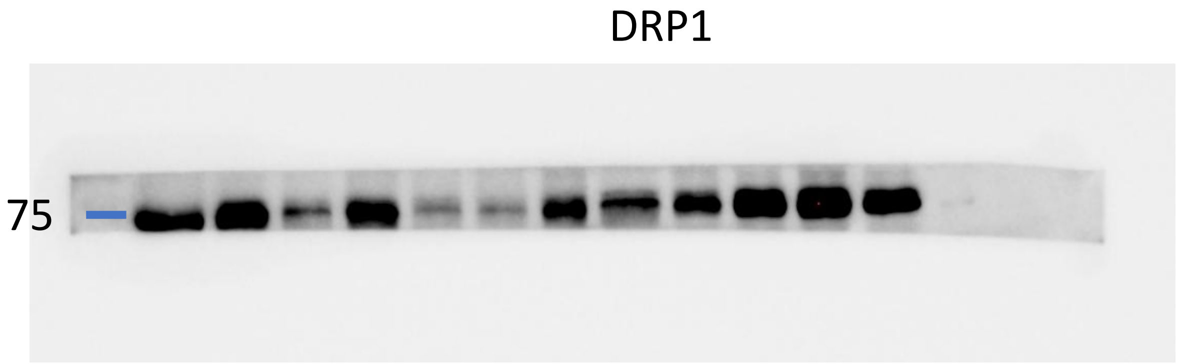
DRP1
75

MDH2

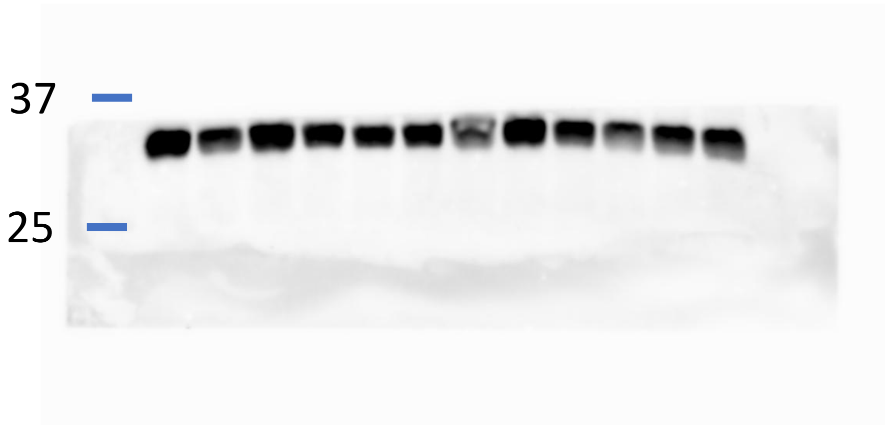

OGDH

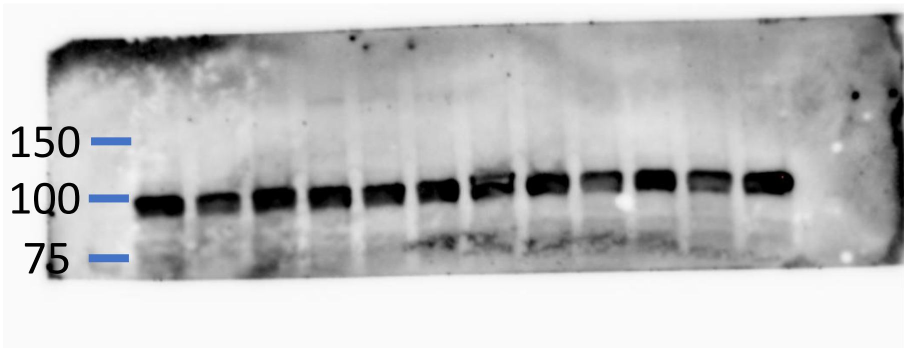

VINCULIN

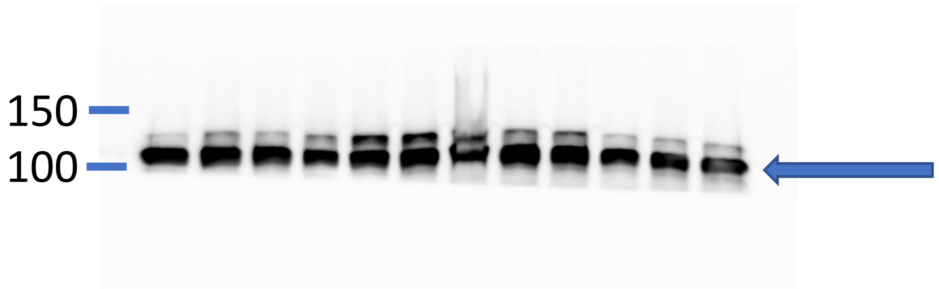

IL-1β

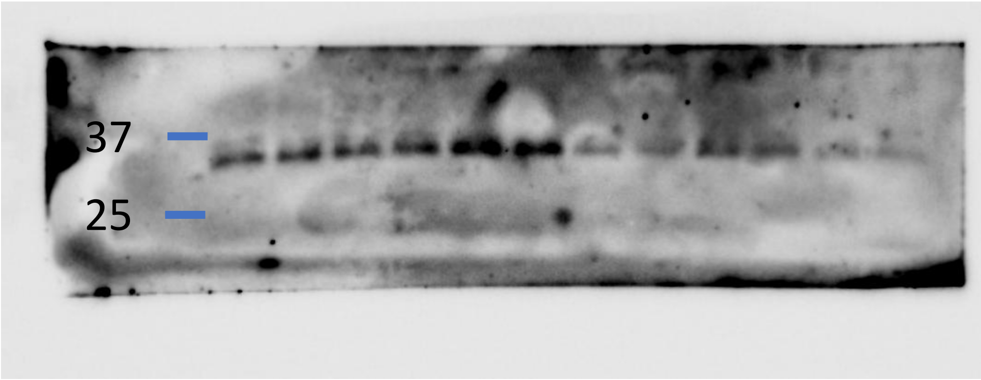

TNF-α

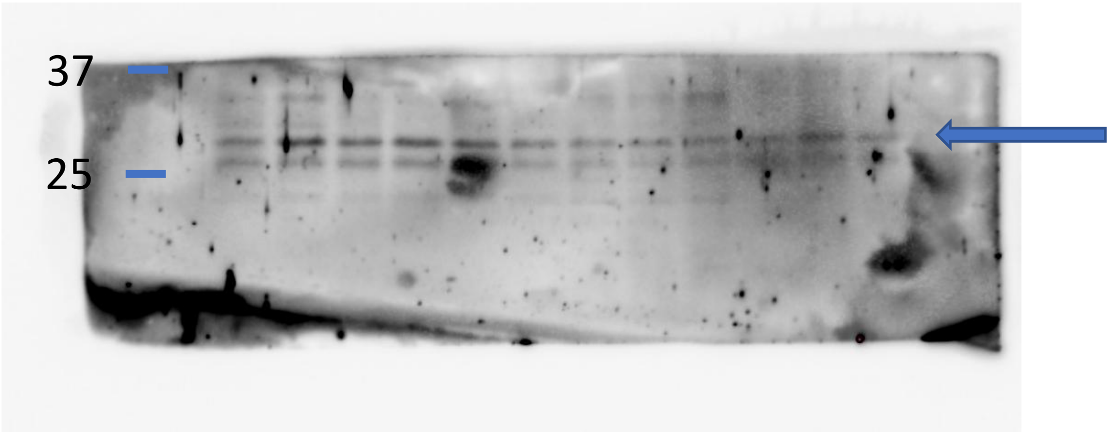

TGF-β

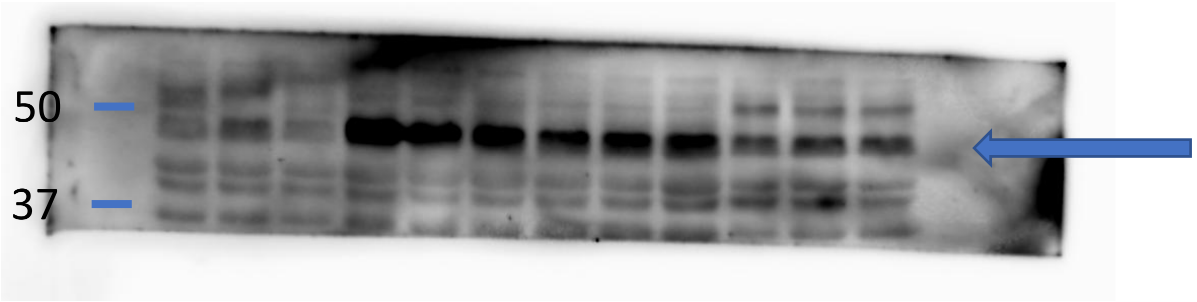

IL6

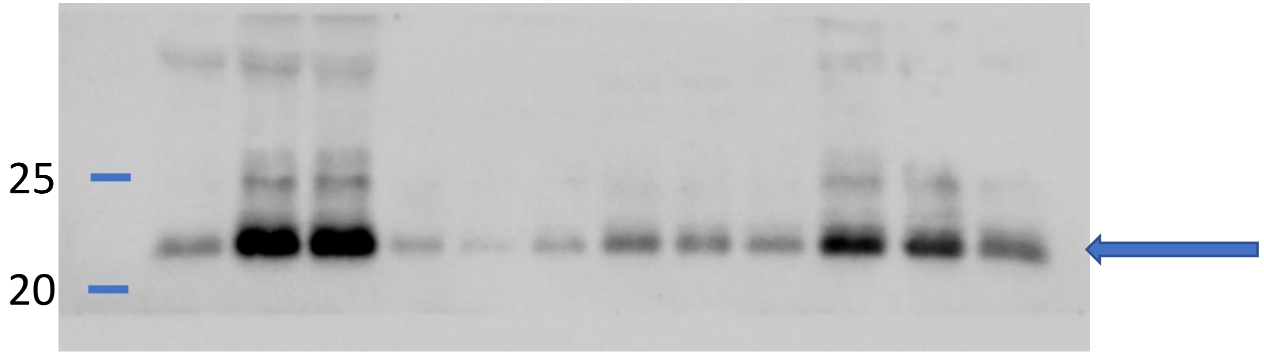

NFkB

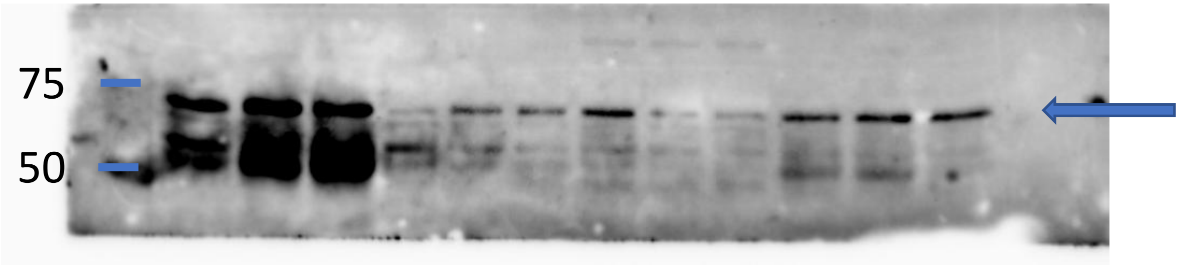

HMGB1

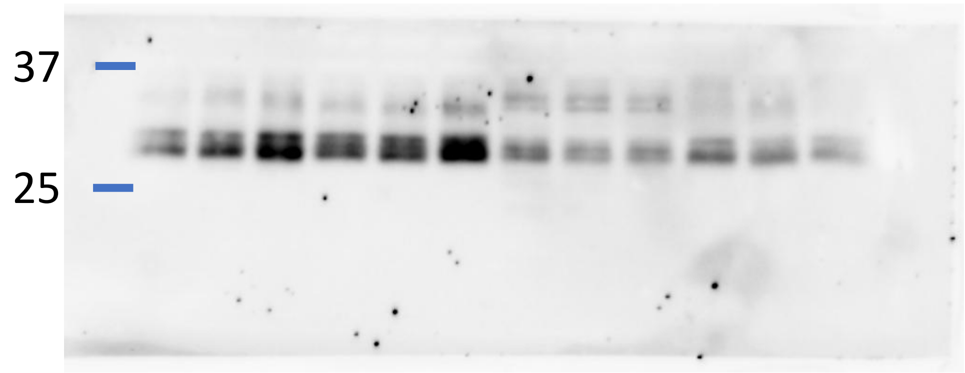

Vinculin

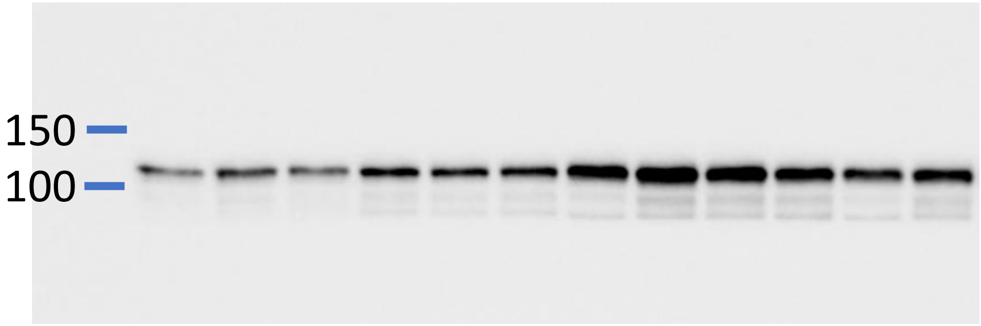

OXPHOS

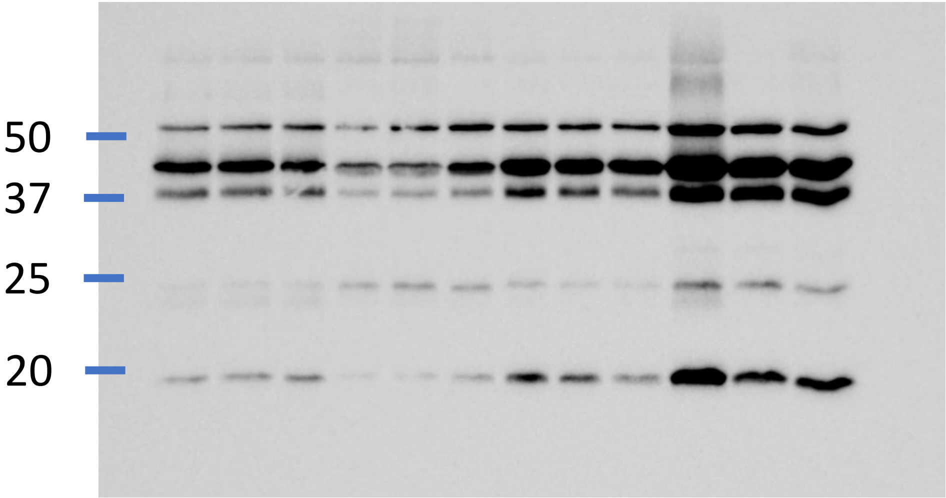

Supplement: Supplementary file 9 — Data S2: Supplementary information. [file JCSM-16-e70054-s001.pdf]
